# High-resolution XRF-CS/ICP-MS mineral element data calibration and potential applications in sub-Antarctic peat records

**DOI:** 10.1038/s41598-026-41047-8

**Published:** 2026-02-26

**Authors:** François De Vleeschouwer, Stephen J. Roberts, Gaël Le Roux, Thomas Bishop, Sarah J. Davies, Angela Gallego-Sala, Charlotte Green, Bianca Perren, Krystyna M. Saunders, Alex Whittle, Anjali L. Dhunna, Dominic A. Hodgson

**Affiliations:** 1 Dpto. de Ciencias de la Atmosfera y los Oceanos, FCEN, Instituto Franco-Argentino para el Estudio del Clima y sus Impactos (IRL IFAECI/CNRS-CONICET-IRD-UBA), Universidad de Buenos Aires, Intendente Guiraldes 2160, Ciudad Universitaria, Pabellon II - 2do. Piso (C1428EGA), Ciudad Autonoma de Buenos Aires, Argentina; 2 British Antarctic Survey (BAS), Natural Environmental Research Council (NERC), High Cross, Madingley Road, Cambridge, CB3 0ET UK; 3 Centre de Recherche sur la Biodiversité et l’Environnement (CRBE), CNRS, Université de Toulouse, IRD, Toulouse INP, Av. de l’Agrobiopôle, 31326 Toulouse, Auzeville-Tolosane France; 4 Department of Geography, Arthur Lewis Building, University of Manchester, Oxford Road, Manchester, M13 9PL UK; 5 Geography and Earth Sciences, Aberystwyth University, Llandinam Building, Penglais Campus, Aberystwyth, SY23 3DB UK; 6 Geography, Laver Building, University of Exeter, North Park Road, Exeter, EX4 4QE UK; 7 Institute for Antarctic and Marine Studies, University of Tasmania, Hobart, TAS 7004 Australia; 8 Department of Geography, Royal Holloway, University of London, Egham, Surrey TW20 0EX UK

**Keywords:** Peat records, Geochemistry, XRF-CS, ICP-MS, Calibration, Southern Hemisphere, Westerlies, Biogeochemistry, Climate sciences, Environmental sciences, Solid Earth sciences

## Abstract

Peatlands are important environmental archives and mineral dust trapped in peat cores from multiple sites can be used to track past changes in hemispheric and global wind circulation patterns. X-ray Fluorescence Core Scanning (XRF-CS) can rapidly geochemically characterise minerals deposited in peat at sub-millimetre-resolution, but calibration is needed to obtain quantitative data. Here, we present a unique calibration of > 14,000 contiguous mm-scale XRF-CS measurements depth-matched to 268 interval-based cm-scale Inductively Coupled Plasma Mass Spectrometry (ICP-MS) quantitative measurements from five peat records located on the west coasts of four sub-Antarctic islands impacted by the Southern Hemisphere Westerly Winds. Of eight calibration models tested, a four-element (Ca, Ti, Sr, Zr) multivariate partial least squares (PLS) model optimised for the widely used dust flux elements Ti and Zr accounts for covariance and provides the most reliable predicted XRF-CS concentrations for Ti (R^2^_CV_ = 0.76, RMSEP_boot_ = 2203 ± 705 mg kg^− 1^, R^2^_pred_. = 0.87, RMSE_pred_. = 2136 mg kg^− 1^, *P* < 0.0001). Predictions for Zr are indicative due to low Zr concentrations, but calibrated Ti and Zr XRF-CS concentrations align well with ICP-MS Ti and Zr concentrations for all five peatland sites. Our multivariate calibration protocol expands the scope of quantitative high-resolution palaeoenvironmental and geochemical research potentially to decadal–centennial timescales for peat records from, and beyond, the sub-Antarctic islands.

**Supplementary Information:**

The online version contains supplementary material available at 10.1038/s41598-026-41047-8.

## Introduction

Reconstructing mineral dust transport pathways from source regions to atmospheric peatland archives provides important information on past climatic and environmental conditions^1,2^. Peatlands act as atmospheric ‘traps’ because of their unique growth characteristics^3^. Unlike other atmospheric archives, such as ice cores^4,5^ or loess, peatlands are distributed globally, allowing past changes in atmospheric conditions to be reconstructed from numerous locations worldwide^6,7^. Cores from peatlands provide insightful data on the origin, transport, and cycling of mineral dust, that reflect past changes in hemispheric-global wind processes^8–10^.

The Southern Hemisphere Westerly Winds (SWW) that encircle Antarctica are the fastest time averaged winds on the planet. Their core belt is located over the Southern Ocean, influencing ocean mixing and the capacity of the ocean to sequester or release CO_2_^11^. Dust records can be used to infer past changes in wind strength, examine the climatic processes that drive dust deposition, and, ultimately, provide an invaluable tool to assess the influence of changing wind patterns on the Southern Ocean CO_2_ sink^7^.

**Fig. 1:**
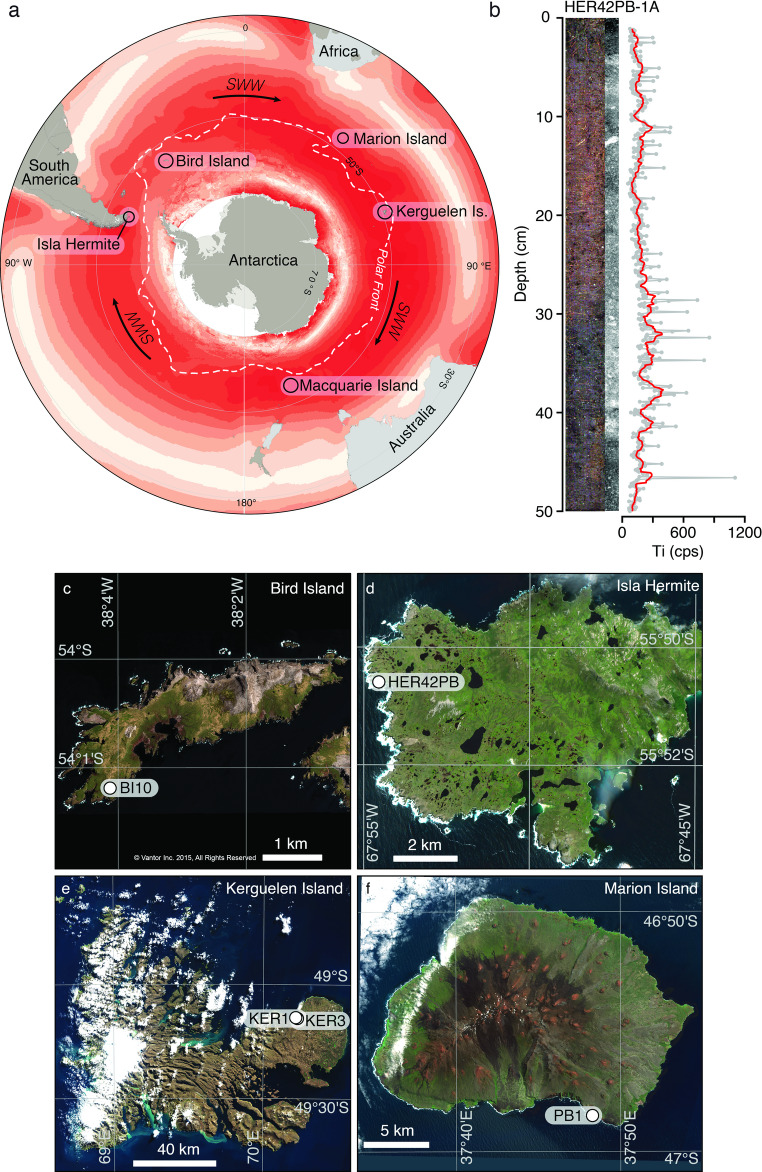
**a** Location of study sites within the core belt of the Southern Hemisphere Westerly Winds (SWW) and in relation to the mean annual position of the Polar Front (PF). **b** Itrax Optical image, negative X-ray image, and 1 mm Ti count per second (cps) XRF-CS data (grey), with 10-point running mean (1 cm) smoothing (red), for the upper 0–50 cm Russian peat core section from site HER42PB on Isla Hermite, Cape Horn, South America, illustrating a typical sub-Antarctic Island peat core matrix. Sub-cm scale higher density lithogenic deposits in the peat matrix are represented by light grey/white areas of the negative X-ray image. **c** Peat core site BI10 on Bird Island (white dot). **d** Peat core site HER42PB on Isla Hermite. **e** Peat core sites KER1 and KER3 on Îles Kerguelen. **f** Peat core site PB1 on Marion Island. Part (c) includes satellite imagery © 2026 Vantor. All rights reserved, used with permission under a NERC-BAS educational + display license, modified and reproduced as a static image at low resolution in ArcGIS Pro software version 3.2, under license conditions, and not included in the Creative Commons license for the article. For permission to reuse the image in part (c), please contact Vantor directly (https://vantor.com). Parts (d), (e), and (f) contain modified Copernicus Sentinel-2 data, 2024 (https://dataspace.copernicus.eu), processed using ArcGIS Pro software version 3.2, compatible with the Creative Commons (CC BY 4.0) license.

Over the last decade, we have visited several islands in the Southern Ocean surrounding Antarctica and extracted peat records to provide a better understanding of the impact that past changes in the SWW have had on high latitude environments of the Southern Hemisphere^7,11–20^. We focused our attention on peatlands in southernmost South America and on the sub-Antarctic islands as they are the only landmasses within the core belt of the SWW (Fig. 1). Peat cores extracted from these locations can be up to c. 18,500 years old^21^ and, together, they provide a detailed record of changes in mineral dust deposition across all sectors of the Southern Ocean. We have previously demonstrated the value of dust in peat records as a sub-Antarctic wind proxy^7^ and also developed independent proxies of past wind strength based on diatoms and testate amoebae responses to changes in sea salt aerosol input into lakes and peatlands^14–16,20^.

In this study, we quantify decadal to centennial changes in long range versus local mineral dust in peat records. Such analysis requires site-specific understanding of local processes such as volcanism, wind, or in some cases anthropogenic erosion^8^, and is traditionally based on quantitative geochemical provenance data obtained from acid digestion and mass spectrometry analyses^22^. Techniques such as Inductively Coupled Plasma – Mass Spectrometry (ICP-MS) produce quantitative analytical data but have a limited temporal resolution due to the physical constraints of subsampling. ICP-MS analyses are also costly, time consuming, destructive and require substantial quantities of peat material, consumables (e.g., high purity acids), and energy consumption. This is not only environmentally damaging, but also prevents very high-resolution datasets from being obtained, constraining the temporal resolution at which palaeoclimatic and palaeoenvironmental reconstructions can be made.

Developments in analytical techniques underpin, and have improved, our ability to investigate past changes in climate and the environment. It is now possible to examine geochemical changes in peat records at smaller measurement intervals, and, therefore, at higher temporal resolutions than previously possible using a variety of high resolution core scanning techniques. In particular, the continued development of non-destructive tools such as X-ray fluorescence core scanning (XRF-CS) provides an opportunity to acquire geochemical datasets from peat cores at (sub)millimetre-resolution^23,24^.

Non-destructive and low-cost XRF-CS scanning techniques are highly efficient, but they do not produce quantitative elemental concentration data required for mineral dust flux analysis. XRF-CS is a ‘semi-quantitative’ Energy Dispersive Spectrometry technique, which has limited application in peat geochemistry without sample-specific calibration. Raw data outputs from XRF-CS are in counts per second (cps), often making direct comparisons between records and instruments with different operational conditions challenging. Data transformation and calibration are therefore critical first steps for identifying the dominant elements contributing to the XRF-CS signal that can be reliably attributed to regional and/or long-range mineral dust inputs.

While calibration has been successfully applied to most mineral-dominated matrices, such as lake and marine sediments^24–29^, research on quantifying XRF-CS data from fresh organic-dominated matrices, such as peat records, is more sparse and challenging. Only a few studies have attempted to calibrate XRF-CS geochemical data from peat, using, for example, Inductively Coupled Plasma – Mass Spectrometry (ICP-MS) or Optical Emission Spectrometry (ICP-OES) analyses obtained by subsampling^23,25,30,31^.

Peat records have several attributes that need to be considered when attempting to independently calibrate XRF-CS to ICP-MS/OES data. Firstly, peat is formed by decaying organic matter from heterogeneous vegetation sources resulting in surface irregularities and changes in porosity. These can create artefacts along the ~ 1 cm wide scanned central part of the core that might not necessarily reflect real changes in the geochemistry^27^. Moreover, (wet) peat is mainly composed of light chemical elements (C, H, O), which cannot be measured by XRF-CS, leading to large coherent (elastic) and incoherent (inelastic) backscatter, often an order of magnitude greater than the background cps values. This feature makes it challenging to obtain quantitative XRF-CS data, particularly for heavy elements with minor or trace concentrations. Consideration also needs to be given to peat sections with relatively higher mineral contents (i.e., matrix effects), or waterlogging which may dilute or attenuate elemental signals^30,32^.

Calibration also needs to include a range of peat matrices from the region being studied, including both organic and high mineral content end members. Differences in instrument performance should be considered, including the condition of the X-ray tubes and detectors, the operating parameters (e.g., kV, mA, interval, time) and different data transformations, such as conversion to percentage cps (%cps) to assess closed sum effects, and incoherent scatter normalisation (inc. normalised), centred-log ratios (clr) and other log element ratios, have been proposed for producing reliable calibrations^24^.

Here, we present a unique calibration between XRF-CS and ICP-MS datasets from five peat cores collected from a restricted latitudinal range (45°– 55°S) on the western coasts of islands in the Southern Ocean (Fig. 1). The sub-Antarctic islands in this study are located within the core SWW belt, providing an ideal opportunity to test XRF-CS to ICP-MS calibration methods as they contain a range of atmospheric mineral inputs from local and long-range sources including dust, sea salt aerosols, and tephra. As we were primarily interested in long-range wind-derived inputs, atmospheric peat records from the western coasts were chosen to maximise the potential to identify minerogenic inputs (and dust) from the prevailing SWW, while limiting the supply of dust from local sources on each island.

We present data from peatlands located on four sub-Antarctic islands (Fig. 1; Table 1):

**Table 1:** Peat core locations and other key metadata. We analysed 279 ICP-MS samples in total in peatland records from five sites (BI10, HER42PB, KER1, KER3, PB1) shown in Fig. 1 (including overlaps), with 268 depth-matched ICP-MS and XRF samples present in their final composite depth records.

Island	Site ID	Latitude	Longitude	Dry Density range (g cm^− 3^)	Modelled mean basal age [cal. a BP ± 95% CI] (Total record depth in cm)	No. of ICP-MS analyses (No. in composite record)
Bird Island	BI10	−54.01980	−38.06876	0.92–1.00	8140 ± 610 (507)	72 (68)
Isla Hermite	HER42PB	−55.84321	−67.90920	0.14–1.01	5130 ± 230 (410)	70 (66)
Îles Kerguelen	KER1	−49.14987	70.22850	0.20–1.58	7960 ± 140 (254)	53 (52)
Îles Kerguelen	KER3	−49.14987	70.22813	0.07–1.08	11,040 ± 160 (204)	53 (48)
Marion Island	PB1	−46.97518	37.80475	0.02–1.07	4720 ± 208 (130)	31 (31)

1. Bird Island (BI10): Cores were collected in November 2017 from ~ 30 m above sea level at Morris Point, which is highly exposed to the westerly winds, and has the highest peatland surface conductivity recorded. A ~ 5 m peat record was extracted in a ‘saddle’ adjacent to a wandering albatross colony with a few birds and no seals close-by. Surrounding slopes are vegetated with tussock grass and the vegetation at the coring site is predominantly a stressed form of tussock grass, with an understory of mosses. The peatland at site BI10 was relatively dry compared to other sites on Bird Island.
2. Isla Hermite (HER42): Cores were collected in November 2015. A ~ 4 m deep record transitioning from lake to peat sediments adjacent to high cliffs on the western side of the island. The site is also very exposed to the westerly winds and wind-blown debris from nearby blow-outs^19^.
3. Kerguelen Archipelago (KER1 and KER3): Two peat cores of 2.6 m and 2.1 m respectively, both containing tephra, were collected in 2018 with triplicate Russian cores from two domed ombrotrophic peat bogs between a cabin and the beach at the Cataractes caboose on the volcanically active Kerguelen Island.
4. Marion Island (PB1): The Puisie Bog area immediately west of Cape Hooker on the volcanically active Marion Island was cored in 2018, recovering a ~ 1.3 m deep peat record containing tephras and volcanic debris from a landscape of recent black lava at the surface, and small peatlands and ponds occupying depressions. A brief survey of peat depths on the salt spray influenced coastal herb field showed the thicker deposits (1.5 m) were located towards the coast where burrowing petrels were nesting on cliff tops. Peatland in depressions and channels or drainage line mires were often underlain by water; hence, coring sites on the small-scale ridges and rises were chosen to avoid these saturated deposits.

Overall, peat cores from these five sites (BI10, HER42PB, KER1, KER3, PB1) were selected to achieve our goal of incorporating a range of material, from organic peat to peat with a more mineral-rich matrix, covering a broad range of carbon concentrations (1.18–58.65%; mean ± 1σ = 37.79 ± 12.75%), dry bulk density (0.02–1.58 g cm^− 3^; mean ± 1σ = 0.31 ± 0.19 g cm^− 3^), water content (21.99–94.75%; mean ± 1σ = 79.10 ± 10.12%), accumulation rate (0.01–1.28 cm yr^− 1^; mean ± 1σ = 0.07 ± 0.07 cm yr^− 1^), and peat dry mass accumulation rate (0.001–0.325 g cm^− 2^ yr^− 1^; mean ± 1σ = 0.019 ± 0.017 g cm^− 2^ yr^− 1^) (Supplementary Table S4).

This study uses the same coring methods, peat core sub-sampling and sample preparation techniques, and ICP-MS and XRF-CS analytical running conditions at all sites (see Methods for details). We undertook log and centred log ratio (clr) data transformations commonly applied to raw XRF-CS counts per second (cps) data, depth-matched raw and transformed XRF-CS datasets to the ICP-MS subsample data and then tested eight different calibration training models using 268 subsamples for ICP-MS from all five sites. We then applied each model to the log-space XRF-CS dataset and converted it to generate predicted concentrations (in mg kg^− 1^) of more than 14,000 individual spectra. Detailed examples and outputs from all eight models are presented in the Supplementary Results.

In the following sections, we summarise outputs from all eight calibration models and present downcore predictions from the most robust and reliable multivariate Partial Least Squares (PLS) calibration model (Figs. 3 and 4; see Supplementary Results for calibration and downcore prediction plots for all models). We compare predicted Ti and Zr XRF-CS concentrations with ICP-MS data and high-resolution multi-proxy datasets from a 5000-year-old peat core record from a key site on one of the islands (Isla Hermite, Cape Horn, Southern Chile) and compare predicted Ti and Zr XRF-CS concentrations with ICP-MS data for the other four sites. We then discuss the substantial multi-centennial to (potentially) sub-decadal timescale gain that can be achieved by XRF-CS analysis of mineral dust deposits in peat cores.

**Fig. 2:**
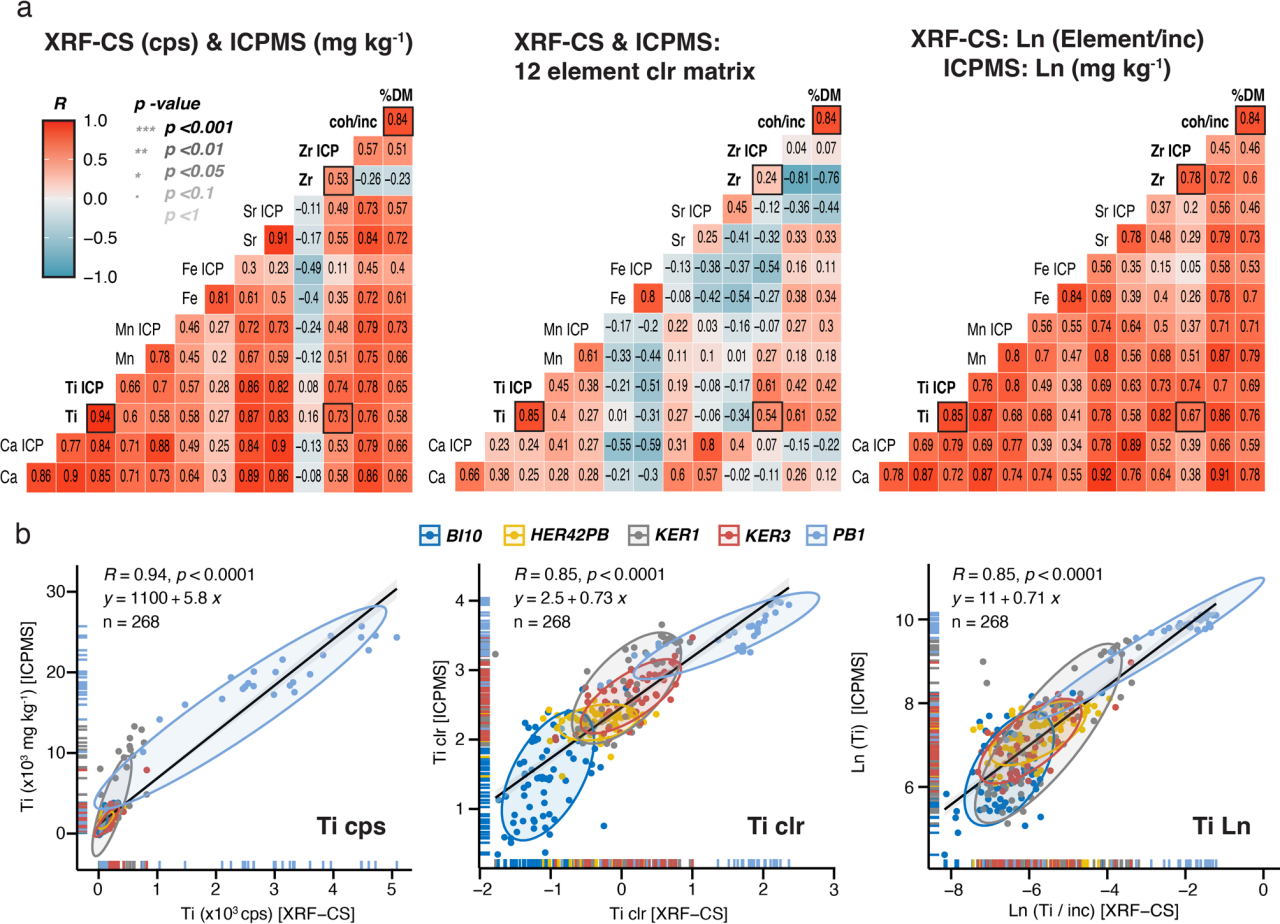
**a** Comparison of correlation results between XRF-CS and ICP-MS data for raw “as measured’’ data (cps vs ppm), centred log ratio (clr) transformed data for six key elements Ca, Ti, Mn, Fe, Sr, Zr (from 12 elements K, Ca, Ti, Mn, Fe, Co, Ni, Cu, Zn, Rb, Sr, Zr defined by applying an autocorrelation function to the XRF-CS dataset to determine elements with downcore variability that is different from noise), and log_n_ transformed element/incoherent scatter XRF-CS data vs log_n_ transformed ICP-MS data from all study sites (Supplementary Results for details). **b** Example correlation biplot for Ti for all site data for the three scenarios described in (a), with 68% confidence interval ellipses for each site (Supplementary Results and Supplementary Fig. S2 – S7 for details).

**Fig. 3:**
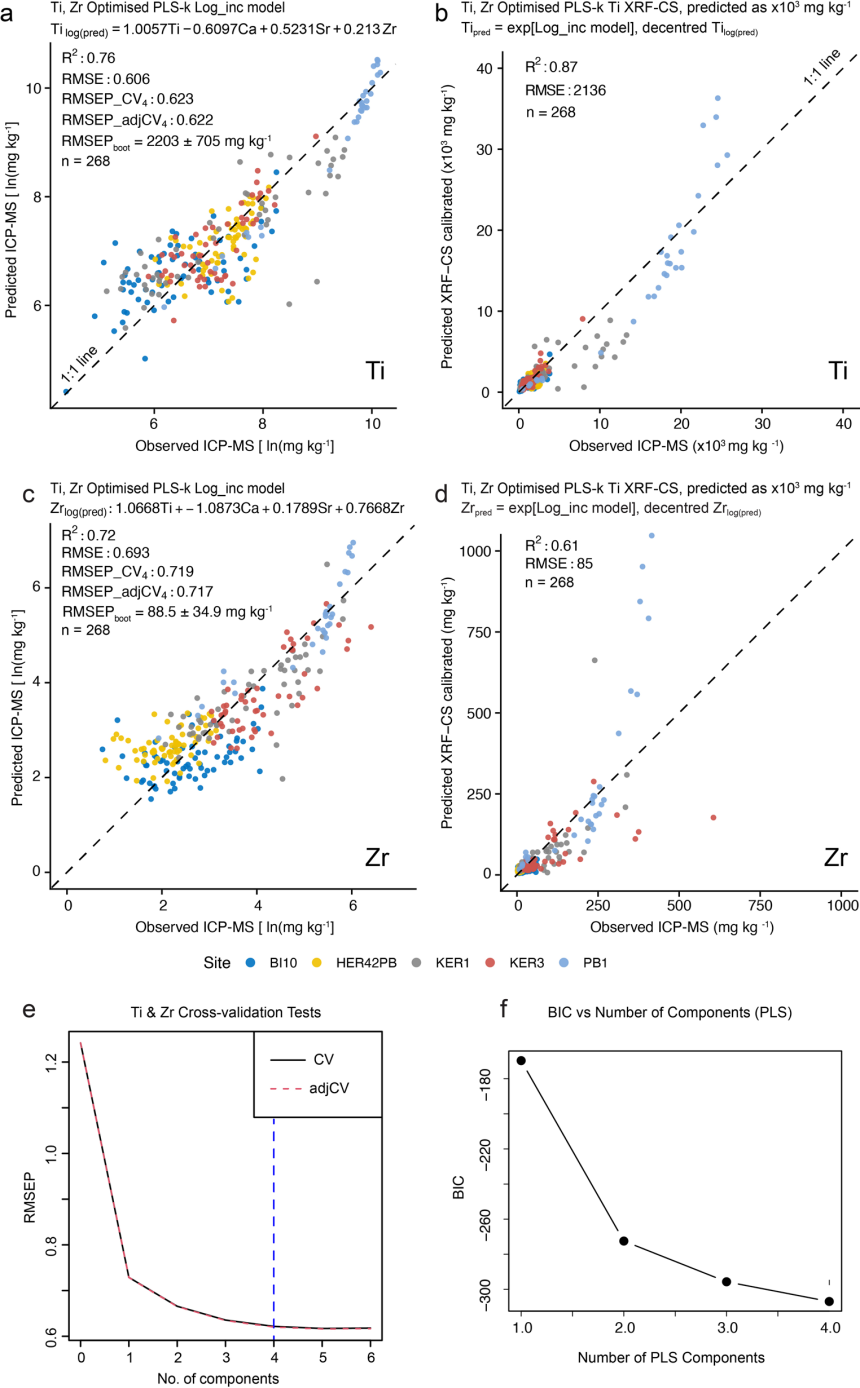
**a** Final multivariate Partial least squares (PLS) (four-component Ti, Ca, Sr, Zr; optimised for Ti and Zr) log-normalised calibration model for Ti based on the matched XRF-CS natural log (Ln) inc. normalised and natural log (Ln) ICP-MS dataset from all five sites; **b** Predicted Ti concentrations, in mg kg^− 1^ generated from the decentred and exponential-transformed PLS training model shown in (a). **c** As part (a), but for Zr; **d** As part (b) but for Zr. **e** Summary of the final 10 fold cross validated PLS calibration model goodness of fit and model complexity, showing four components are optimal for PLS calibration training model, i.e., where RMSE and RMSEP values stabilise and align; **f** Bayesian Information Criteria (BIC) results calculated from total residual variance across the final PLS training regression model run with four elements (Ti, Ca, Sr, Zr) (Methods, Supplementary Methods for details).

**Fig. 4:**
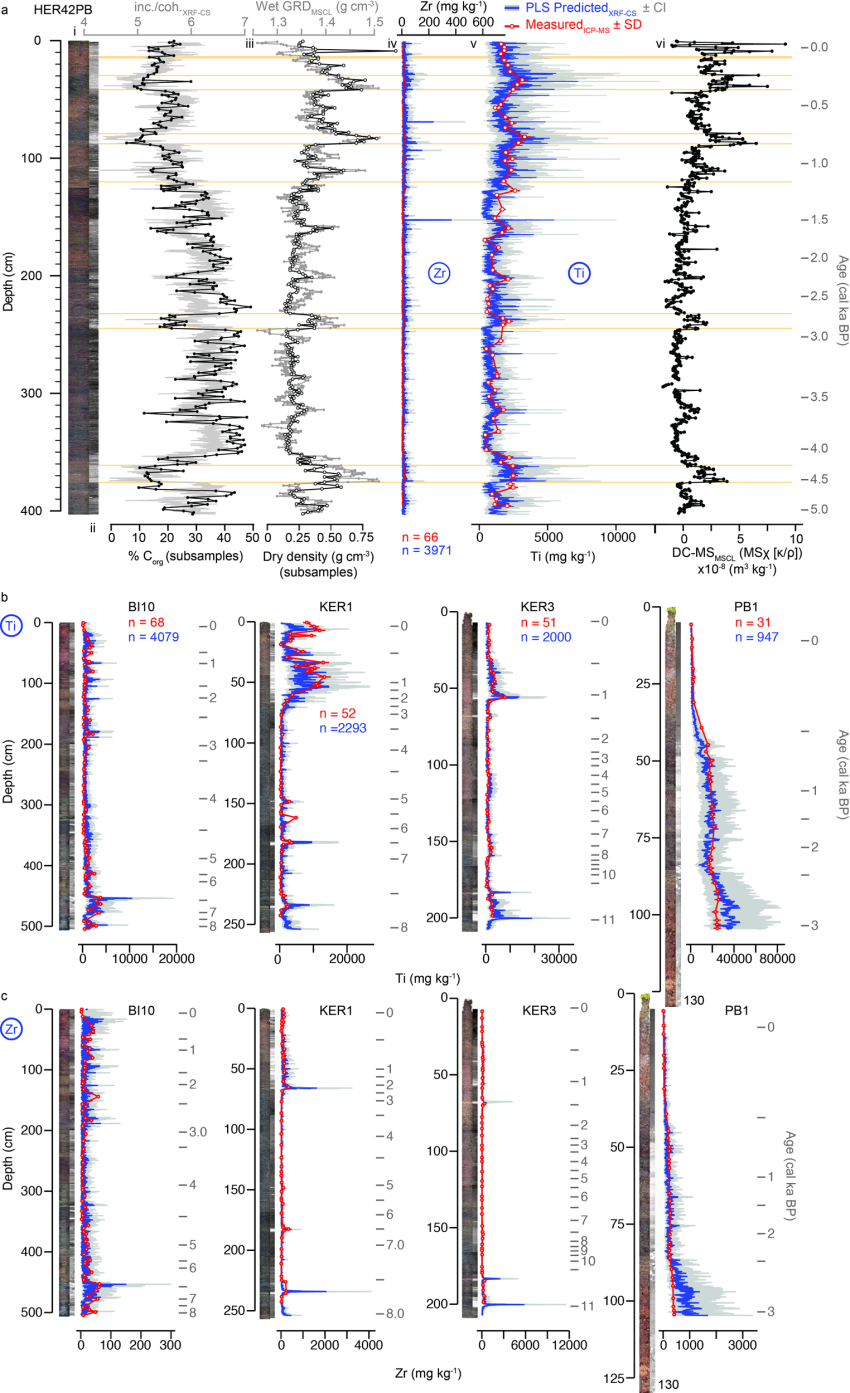
**a** Multi-proxy scanning and subsample downcore data for the HER42PB peat record from Isla Hermite, Cape Horn, South America. From left to right: (i) high-resolution negative X-ray image (lighter is higher density and more minerogenic); (ii) XRF-CS incoherent/coherent ratio (grey) and organic carbon (black) (calculated from loss-on-ignition data and the SOM/SOC ratio of peat composed primarily of vascular plants of 1.73 ± 0.09^46^; (iii) multi-sensor core logger (MSCL) wet gamma ray density (GRD) data measured at 5 mm intervals (grey) and subsample dry density (black); iv) and v) Measured Zr and Ti ICP-MS subsample data (red circles, line; at ~ 1 cm intervals) compared with 3971 predicted XRF-CS Ti concentrations (in mg kg^− 1^) generated by the final PLS calibration model for HER42PB (blue line, 95% confidence intervals grey lines); and (vi) Volume specific magnetic susceptibility measured on the MSCL. The Ti, MS and density parameters are proxies of minerogenic sediment in the peat matrix, illustrating the increased downcore precision and level of detail achievable by calibrating XRF-CS and ICP-MS data. Yellow solid horizontal lines (shown for site HER42PB only) are CONISS-defined constrained cluster analysis zones based on the 5 mm MSCL-GRD and MSCL-MS data only (cluster diagram not shown here for clarity). Age profiles are shown in grey as a secondary axis on the far right; **b** Quantitative predictions of Ti, in mg kg^− 1^, for peat profiles from sites BI10, KER1, KER3 and PB1; **c** Quantitative predictions of Zr, in mg kg^− 1^, for from sites BI10, KER1, KER3 and PB1. Age depth models for all records are shown in Supplementary Fig. S19^19^.

## Results

Prior to calibrating XRF-CS and ICP-MS datasets, we undertook the following preparatory steps:

(i) *XRF-CS spectra filtering*: This critical first step followed a robust quality control workflow in *itrax*.R v.1.12.2^33^ to remove potentially spurious spectra, reducing the number of reliable XRF-CS spectra across the primary calibration dataset from all peat cores collected from sub-Antarctic islands 14,579 to 14,513 spectra (> 99% retained) (see workflow summarised in Supplementary Fig. S1). At this stage, we removed any spectra with: (1) exceptionally elevated MSE values greater than its mean + 8σ, which represent a comparatively poor fit between measured and modelled spectra, often associated with air gaps or a matrix of non-sedimentary origin; (2) exceptionally low or high total cps greater or less than its mean ± 8σ, mainly due to gaps in the cores (low counts) or indicative of a matrix of non-sedimentary origin (i.e., high counts > 120,000 cps); and (3) surface slope outliers greater/less than its mean ± 2σ, indicating areas of the core that were not sufficiently flat for the detector to remain completely level during analysis. Overall, the remaining spectra had a mean ± σ MSE value of 1.35 ± 0.10, indicative of a reliable and robust spectra matching process (Supplementary Table S2). Measurement errors applied into ICP-MS and XRF-CS data are as detailed in the Methods.
(ii) *XRF-CS element filtering*: An autocorrelation-based function (*acf* in base R) filtering process was undertaken as part of the *itrax.R* quality control data processing procedure (see Methods for details). A conservative ‘minimum’ correlation threshold of 0.1 after a lag of 20 XRF-CS measurement intervals (equivalent to twice the ICP-MS subsample thickness of ~ 1 cm) left 19 elements from XRF-CS analysis whose downcore patterns were statistically different to noise (Si, S, Cl, K, Ca, Ti, V, Cr, Mn, Fe, Co, Ni, Cu, Zn, Br, Rb, Sr, Zr, Ba, along with incoherent (inc.) and coherent (coh.) scatter parameters). Of these 19 XRF-CS elements, 12 elements (K, Ca, Ti, Mn, Fe, Co, Ni, Cu, Zn, Rb, Sr, Zr) could be compared directly to elements in the ICP-MS dataset. Only six ‘key’ elements from this list (Ca, Ti, Mn, Fe, Sr, Zr) were above a more stringent correlation threshold of 0.5 after a lag 20. These six elements also performed best in preliminary univariate correlation and regression analysis (Fig. 2; Supplementary Fig. S2–S6) and were therefore considered most suitable for multivariate calibration.
(iii) *XRF-CS and ICP-MS depth-matching*: Using established composite records to avoid overlapping issues, the filtered XRF-CS spectra and elements were then matched to ICP-MS data across the subsample depth intervals for each record with the *itraxR::itrax_reduce()* function^33^, with mean ± σ XRF-CS cps values calculated for each of the 12 elements and for each ICP-MS data point (Supplementary Fig. S1).
(iv) *Optimising and transforming XRF-CS data for calibration*: As XRF-CS scans were carried out on natural wet peat samples with variable porosity, density, water and mineral content, a series of dataset transformations (%cps, natural log element/inc. normalised, and clr) were investigated. The aim of this was to minimise matrix effects and provide values as closely comparable to dry and organic free ICP-MS data as possible. Normalising XRF-CS data by incoherent scatter takes changes in density, water content and organic matter into account and substantially improved correlation coefficients for individual elements across the whole dataset (and for individual sites) (Fig. 2a).

Following initial dataset structure mapping (e.g., Fig. 2, Supplementary Fig. S2), preliminary covariance and correlation (Pearson) analysis (Supplementary Fig. S3–S5) and simple ordinary and weighted ordinary least squares (natural) log and clr univariate (OLS/WLS) linear regression models were assessed for each of the 12 elements at the five sites individually, and then for all the sites combined (Supplementary Fig. S6–S8; in these plots, ICP-MS data was the response, y, variable and XRF-CS was the predictor, x, variable). Comparing univariate raw cps XRF-CS element data distributions to ICP-MS data highlighted that some lithogenic elements (e.g., Zr) had low background concentrations close to detection limits displayed exponential rather than linear relationships, even after clr transformation (Fig. 2b; Supplementary Fig. S2d).

For XRF-CS analysis, measurements were made on raw (and wet) material, hence the influence of grain size would be more noticeable and important towards the upper ICP-MS limit, “bending” the data distribution towards infinity, as shown most clearly by Zr (Figs. 2b and c and 3, Supplementary Fig. S6a). ICP-MS concentrations have theoretical maximum concentrations for each element as well, but because measurements were made after complete sample digestion there is no grain size effect. At the lower end of the correlation, the principal limitation is the XRF-CS detection limit. In organic matrices in particular, elements such as Ni, Zn and Zr often have very low XRF-CS cps counts whereas ICP-MS data has a much lower detection limit, and those elements can be measured more accurately. For XRF-CS data, Ni, Zn and Zr tended ‘cluster’ around zero cps, but a zero-skewed dataset distribution was also noticeable for some other elements, such as Ca, Ti, Sr with higher cps (Supplementary Figs. S3–S6).

(v) *Preliminary and Final Calibration*: Using the matched natural log-spaced incoherent scatter normalised XRF-CS dataset and the log-spaced ICP-MS dataset, we evaluated eight calibration training models, consisting of the four previously described univariate correlation and regression models (OLS, WLS, weighted OLS, weighted WLS) and four multivariate models: a Bayesian Generalised Linear Model (Bayes glm), Random Forest (RF), Partial Least Squares (PLS) with 10-fold cross validation (CV), and PLS with leave-one-out (LOO), using six key elements (Ca, Ti, Fe, Mn, Sr, Zr) and four elements (Ca, Ti, Sr, Zr) individually for univariate models and simultaneously for multivariate models (Methods, Supplementary Methods for details).

Although calibration model performance metrics for all eight models were broadly similar (Supplementary Fig. S9, S10), the two PLS calibration models (CV and LOO) run with four elements (Ca, Ti, Sr, Zr) proved to be the most robust multivariate models for predicting quantitative concentrations of Ti (in mg kg-1) for the > 14k XRF-CS five-site dataset (R^2^_CV_ = 0.76, RMSEP_boot_ ± σ = 2203 ± 705 mg kg^− 1^, R^2^_pred_. = 0.87, RMSE_pred_. = 2136 mg kg^− 1^, *p* < 0.0001 cf. ICP-MS_mean_ ± σ (SE) = 3392 ± 5632 (344) mg kg^− 1^; Fig. 3, Supplementary Fig. S10–S17, Table S5). Reducing the number of model components (elements) from six (Ca, Fe, Mn, Sr, Ti, Zr) to four (Ca, Sr, Ti, Zr) to create a Ti- and Zr- optimised (dust-flux) PLS calibration model resulted in a marginal increase in R^2^_CV_ from 0.72 to 0.76 for Ti (Fig. 3, Supplementary Fig. S9a, c, Table S5a, c).

In our final four-element Ti and Zr optimised PLS model (Fig. 3), Ti, Ca, and Sr had the most significant influence on Ti predictions, but Sr had no significant influence on Zr predictions (Supplementary Box 2, 4). Signal-to-noise ratio (SNR) and SNR-confidence interval and smoothness parameters identified this PLS model was the best model for predicting Ti and Zr concentrations downcore, with a good fit to the ICP-MS data for all sites. PLS models were consistently better at downcore prediction than the Random Forest model, which tended to be dominated by excessive short-scale variability relative to signal amplitude (i.e., calibration-model generated noise, Supplementary Fig S13–S17). Predictions from the RF model often far exceeded measured ICP-MS values and errors as well, even when its performance indicators (R^2^, RMSE, RMSEP) outperformed the other multivariate models (PLS and Bayesian).

For Zr, predicted concentrations were consistently low, apart from, most notably, exceptionally elevated concentrations in some records associated with the presence of crypto-tephra. The six-element Random Forest calibration model performance metrics were better for Zr than the four-element PLS model (R^2^_CV_ = 0.76 _RF6_ vs. 0.72 _PLS4_, R^2^_pred_. = 0.93 _RF6_ vs. 0.61 _PLS4_). As for Ti, the four element PLS model predictions were significantly less ‘noisy’ and more stable, providing a better fit to the ICP-MS data for all sites (PLS-4 elements: RMSEP_boot−PLS4_ mean ± 2σ = 89 ± 70 mg kg^− 1^, RMSE_pred_. = 85 mg kg^− 1^ and RF-6 elements: RMSEP_boot−RF6_ mean ± 2σ = 48 ± 22 mg kg^− 1^, RMSE_pred−RF6_. = 27 mg kg^− 1^ compared to ICP-MS_mean_ ± 2σ (SE) = 66 ± 194 (6) mg kg^− 1^) (Fig. 3, Supplementary Fig. S10–S17, Supplementary Tables S4, S5a, c). However, measured elemental concentrations were persistently low for Zr and a six-element Random Forest calibration model might prove more reliable for Zr dust flux reconstruction for some peatland sites (Supplementary Fig. S13–S17). Additionally, the Zr ICP-MS concentrations are not currently certified for the Certified Reference Materials (CRM) standard used (Supplementary Table S2) and this aspect could also be improved in future studies. Therefore, the calibration for Zr presented here is indicative only and ICP-MS summary data and calibration model performance metrics above are reported with ± 2σ errors.

(vi) *Calibration model performance tests*: To test the reproducibility of all calibration models and predictions, we compared univariate and multivariate log-space outputs for all models with 12-, 6- and 4-element XRF-CS, and also tested the final PLS calibration model using centred log ratio (clr) matched matrix datasets (Supplementary Results for details). Normality distribution (Shapiro-Wilks), residual, and homoscedasticity analysis (Breusch-Pagan Test^34^ revealed that heteroscedasticity was commonplace for all univariate models, indicating residuals were not distributed equally (i.e., non-constant error variance existed). For univariate models, inverse weighting helped to reduce the effects of selection bias as well as issues associated with missing data, but, overall, in terms of performance metrics, univariate models were consistently outperformed by multivariate models in six and four element runs (Supplementary Table S5b, d; Supplementary Results for details).

In summary, our calibration model comparison exercise and performance tests showed that it is possible to combine sites with similar matrix characteristics, but also that no one calibration model is best at predicting robust and stable XRF-CS values for several elements simultaneously, even when they are covariant. Cross-validated four-element (Ca, Ti, Sr, Zr) PLS calibration models optimised for Ti- and Zr-prediction consistently produced the most stable, robust and reliable Ti and Zr predictions, avoiding overfitting and comparatively poor predictions of more complex models (e.g., tree-based probabilistic RF and machine-learning models) – most likely because PLS models are inherently designed for high covariance encountered in the XRF-CS (and ICP-MS) datasets. For these reasons, PLS was selected as our preferred calibration modelling approach.

## Discussion

Previous attempts at inter-site comparisons of mineral dust records from peat cores have been hampered by the variety of different analytical and data analysis techniques employed. Moreover, the low temporal resolution of most subsample-based measurements has limited both our capacity to investigate centennial to decadal-scale changes in dust fluxes, and, more generally, our ability to compare datasets between records from several sites precisely.

This study is a critical first step in producing consistent, reliable, and high-resolution quantitative inorganic geochemical data at from multiple peat records, allowing us to assess their potential as invaluable high-resolution dust flux records across the entire Southern Ocean at centennial to decadal timescales. Our approach to assessing prediction performance, using traditional metrics (cross validated and bootstrapped R^2^, RMSE, RMSEP), a simple signal-to-noise ratio (SNR) based assessment of calibration-generated noise^35^, and a model robustness classification scheme (Supplementary Table S5) to quantify XRF-CS predictions, is consistent with previous XRF-CS and palaeoenvironmental reconstruction studies that have treated potentially unstable prediction behaviour as analytical or calibration noise rather than being representative of geochemical (or other) changes in the matrix that are driven by environmental change^36–40^.

Our comprehensive analytical process and calibration workflow, from the input of raw spectra and concentration data through to a final multivariate PLS calibration model for the log incoherent normalised XRF – log ICP-MS dataset, can be used for high resolution prediction elsewhere (Supplementary Fig. 1). The main advantage of using a PLS calibration model is that it is a well-established multivariate method which compresses complex information into a small number of stable and significant latent components that explain the maximum covariance between predictor and response variables^24,41^ (Fig. 3, Supplementary Fig. S11c).

Quantitative predictions for Ti and Zr produced by four-element multivariate PLS (and Bayesian) models are well-aligned with measured ICP-MS downcore quantitative concentrations (in mg kg^− 1^) (Fig. 4, Supplementary Fig. S13–S17). This is most likely because we limited the number of variables used in the final models to the four elements that cross-validation and jackknife tests revealed had the most significant influence on Ti and Zr calibration. Additionally, our matched XRF-CS and ICP-MS dataset represents a broad range of geochemical compositions found in peat matrices from sub-Antarctic islands, covering organic-rich peat to minerogenic deposit end members, and all points in between (Supplementary Fig. 12, Table S4). The number of subsamples chosen per record, and across all sites, proved to be more than sufficient for calibration purposes^24^.

Overall, prediction metric outcomes for Ti and Zr from the Ti- and Zr-optimised PLS model are better, and the predictions themselves are more stable and robust, than simple univariate (WLS, OLS and weighted OLS, WLS) and multivariate RF models, irrespective of whether heteroscedasticity was present or not. Differences between predicted concentrations for three (Ti, Sr, Zr) of the four modelled elements across most models and all sites are not obvious, suggesting our multi-site dataset is sufficiently large, robust and reliable (Figs. 3 and 4; Supplementary Fig. S13–S17).

Elements such as Ca with multiple potential sources are well-measured and generally well-correlated with Ti (*R* ≥ 0.7 for cps and log normalised in Fig. 2a; Supplementary Fig. S3–S8), suggesting a predominantly minerogenic source for both elements. Fe, Mn are also well-measured by XRF-CS and also display consistently strong and significant linear correlations between XRF-CS and ICP-MS for log and clr datasets, but these elements have multiple sources, often reflecting redox changes in peat deposits (Fig. 3a, d, Supplementary Results, Supplementary Figs. S6, S8).

In contrast, Zr, which is commonly used as a lithogenic, conservative element, tended to cluster close to zero across all calibration models, reflecting generally low ICP-MS concentrations (Fig. 3; Supplementary Fig. S9, S10). This was particularly true for peat deposited on volcanically inactive islands, such as Bird Island (BI10) and Isla Hermite (HER42PB) (Supplementary Fig. S11a, b). Mineralogical and grain size effects affecting the XRF-CS data and closed-sum effects for both XRF-CS and ICP-MS datasets are reduced when using the incoherent-normalised XRF-CS log-space dataset, and this improved the overall correlation coefficients substantially compared to raw cps correlation analysis (Fig. 2).

However, towards its upper limit, Zr, and to some extent Ti, both have an apparently non-linear (exponential) relationship in log-space (Fig. 3d; Supplementary Fig. S6, S9, S10), likely driven by grain size differences, and/or increased concentrations of lithogenic elements with peat, basal lacustrine and/or volcanically derived sediments in some records (e.g., towards the base of the PB1 record from (volcanic) Marion Island; Fig. 4b, c; Supplementary Fig. S17), since Zr and Ti are generally contained in accessory minerals and are the harder to erode than most rock forming minerals. This is probably why some predicted quantitative Zr XRF-CS values far exceeded measured ICP-MS data (Fig. 4d; Supplementary Fig. S17). For this reason, the XRF-CS to ICP-MS calibration for Zr presented here is only indicative. In summary, the main issues were many near zero values from both ICP-MS and XRF-CS measurements, which suggest limited Zr, or that detection limits had been reached, while non-linear responses occurred across multiple models at elevated concentrations (Supplementary Fig. S10).

Principal Component Analysis (PCA) revealed that peat records from islands with active volcanoes (Marion Island and Kerguelen Island) are geochemically distinct from non-volcanic islands (Bird Island and Isla Hermite) (Supplementary Fig. S11). Records from volcanically active islands also contain a limited number of well-defined fine (i.e., sub-cm) volcanic ash (tephra) layers from local eruptions, sometimes visible in X-ray imagery (e.g. Fig, 1b). Following the initial detection of exceptionally elevated Zr peaks by XRF-analysis^42^, in, for example, KER1 and KER3 (Fig. 4c; Supplementary Fig. S15, S16), ongoing work concentrating and point counting volcanic glass shards under the microscope has revealed that well-defined, and potentially far-travelled, airfall and reworked crypto-tephra, invisible to the naked eye, exists in all five peatlands records in this study. It appears that reworked crypto-tephra forms part of the general background ‘lithogenic input’ signal in peat records from the sub-Antarctic islands, and, therefore, bulk XRF-CS and ICP-MS measurements impacted by (crypto-)tephra were retained as part of the calibration dataset.

In lake and marine core studies^24,29^, incoherent and coherent scattering ratios, coh./inc. and inc./coh. are often correlated, providing additional context and high resolution proxies for changes in dry mass, which reflects changes in mineral input (coh./inc.)^32^, and water and/or organic content (inc./coh.) (Fig. 4a). Our analysis supports previous evidence^30^ that shows incoherent and coherent scatter ratios are not as strongly correlated with changes in water and/or carbon content in peat records, as they are in more inorganic lake or marine sediments (Supplementary Fig. S12h). However, unlike previous studies of peat deposits where minerogenic deposits are largely absent, mineral input occurs at all sites within our combined dataset. Near constant lithogenic input at some sites underpins the significant correlation between dry mass and the coh./inc. ratio across all peatland sites (R^2^ >0.60; *P* < 0.0001; *n* = 1115), and in the ICP-MS composite depth-matched subsample dataset (R^2^ > 0.62; *P* < 0.0001; *n* = 268) (Supplementary Fig. S12a, b). Conversely, at all sites, bulk carbon content ranges from 1.18 to 58.65% (Supplementary Table S4) – sufficiently wide enough to produce a strong and significant correlation between organic carbon content and the XRF-CS inc./coh. ratio (WLS_wt_ R^2^ = 0.79, *n* = 1041; Supplementary Fig. S12h).

Across our peat records, the relationships between XRF-CS scatter ratios and dry mass or organic carbon appear to be controlled by variations in mineral input at individual sites rather than relative increases/decreases in organic content. At sites with only sporadic, or comparatively low, mineral input, such as BI10 on Bird Island, coh./inc. and dry mass are poorly correlated (R^2^ ≤ 0.46, *n* = 419; Supplementary Fig. S12c), but a higher correlation exists in the HER42PB record from Isla Hermite where minerogenic input is constant (R^2^ ≤ 0.63, *n* = 299; Fig. 4a; Supplementary Figs. S12d, S13, S14). The inclusion of basal sediments from three of the records, PB1, KER1, and KER3, in the calibration allowed us to assess the geochemical composition of the local bedrock input at three out of five sites. We found an exceptional strong and significant correlation between dry mass and the coh./inc. ratio at site PB1 on Marion Island (R^2^ >0.93, *n* = 74) where the widest range of depositional types are present, ranging from lithological sediments deposited in a ‘sub-aqueous’ lacustrine environment near the base of the record, through to ‘sub-aerial’ mineral-rich and mineral-poor organic peat that constitutes the rest of the record (e.g., Supplementary Figs. S12g, S17).

To summarise, our key finding from this study is that applying multivariate calibration models to combined XRF-CS and ICP-MS datasets from multiple sites offers a potentially substantial increase mineral element concentration data resolution, far beyond interval subsampling methods, down to, potentially, decadal-centennial timescales that were previously unattainable for individual peat cores or sites. The most promising minerogenic element target is Ti, an element that has been used widely in global dust flux calculations based on discrete ICP-MS datasets^1^.

Over the last two decades, we have collected high-resolution XRF-CS datasets from a wide range of different terrestrial archives, including peat cores from the western coastlines of several sub-Antarctic islands, and have compared them to novel environmental proxies, such as diatom-conductivity transfer functions^14,16,20^, seabird faecal matter^13^, and dust traps and fluxes^7^ to investigate past changes in the SWW strength, sea-ice, and oceanic CO_2_ exchange dynamics^3,7,14,20^.

Converting XRF-CS data from counts into “concentrations” now opens up numerous possibilities in peat palaeogeochemistry and for palaeowind reconstructions that were previously always limited, not so much by the peat accumulation rate itself, but because discrete analyses could only be performed on selected samples of, at best, ~ 1 cm thickness and usually at widely spaced sampling intervals. Across our whole dataset, the mean ± σ sampling interval for the XRF-CS dataset is 2.5 ± 2.7 years (based on 1 mm contiguous interval measurements) – i.e., ~ 126 times greater than the mean interval gap of 314 ± 225 years between successive ~ 1 cm thick ICP-MS sub-samples (Supplementary Table S8, S9). For peatlands on individual islands impacted by increased lithogenic inputs, XRF-CS provides far greater precision in defining lithological zones that possibly represent periods of increased SWW intensity (Fig. 4a).

Therefore, the calibration process described in this paper provides a step-change in the interpretative power of geochemical data drawn from peat cores. After calibration, XRF-CS analysis of peat opens possibilities of palaeoatmospheric reconstructions at an extremely high resolution for this globally distributed archive. As samples in this study display substantial variation in the matrix composition, from almost ‘pure’ organic peat to mineral-rich peat, the calibration protocol developed in this paper is potentially applicable to other peat records from within and beyond the sub-Antarctic region. We encourage open sharing of similar datasets to test this hypothesis further.

## Methods

All cores were collected using a 50 cm-long, 5 cm-wide Russian corer^43^ from the centre of each peatland area. Cores were protected in PVC tubes, wrapped in plastic film, frozen and vacuum-packed (when frozen) for transport and storage.

### Non-destructive scanning analyses

Intact half cores were slowly defrosted while vacuum-packed to minimise shrinkage and analysed with a non-destructive GEOTEK multi-sensor core logger (MSCL)^44^ to obtain gamma-ray wet density (ɣ-density; GRD), resistivity and magnetic susceptibility (MSκ; SIx10^− 5^) data (Bartington Instruments: MS2E point sensor, 2–5 mm interval; 10 s measurement time) and volume specific (density-corrected) MSχ (κ/ρ; kg m^− 3^) using Geotek MSCL v. 7.9 processing software installed with the machine (https://www.geotek.co.uk). Digital X-radiographs were obtained using a Cox Analytical Systems Itrax X-ray fluorescence Core Scanner (XRF-CS) at Aberystwyth University fitted with a Molybdenum (Mo) anode X-ray tube (settings: 45 kV, 50 mA.ms, 200 ms, 60–200 μm measurement interval). Contiguous and non-destructive downcore XRF-CS was then undertaken using the same core scanner at Aberystwyth University (settings: 30 kV, 50 mA, count time 10 s per 1 mm distance travelled by the detector) (Supplementary Fig. S1). As surface water pooling is known to increase scatter, excess surface water was allowed to evaporate prior to scanning. Cores were then covered in a geochemically inert, ultra-thin (2 μm) film to prevent drying out during analysis. XRF-CS performance checks were undertaken on synthetic reference glass (CS41) and (informally) using XRF fused glass discs from sediment cores extracted from Ardley Lake and Yanou Lake on the maritime Antarctic South Shetland Islands that cover a range of inorganic deposits to organic sediments similar to peat^13^.

The Itrax XRF Core Scanner has a beam size of ~ 13 mm x 0.1 mm, collimated sharply using a capillary waveguide, with the silicone-drift detector nozzle positioned a very short distance from the core surface at 45 degrees to the beam. The detector set up is mechanised to keep the geometry consistent across the topography of a core, and the deviation of each measurement from the nominal geometry is calculated from the laser rangefinder derived topographic data and measurements excluded where they exceed a small threshold^45^. Penetration depth is variable depending on the atomic number of the analyte and the average atomic number of the matrix, which is why raw count per second (cps) measurements have been normalised as described below, and why tube excitation voltage was kept consistent across the study^45^.

Count per second (cps) XRF-CS data were produced for a large range of elements using Cox Analytical Systems Q-Spec software v8.6.0 installed with the machine (https://www.coxsys.se), aiming for as low mean square errors (MSE) as possible, indicating an optimal fit between ‘as measured’ and modelled spectra. Cores were collected over a period of four years between 2015 and 2019 from each of the field sites, and XRF-CS measurements were made as soon as possible after core had returned to the UK. In that time, a new detector with higher count rates and new Mo-tubes were fitted to the Aberystwyth Itrax core scanner in 2016. To account for this, as well as count rate decline associated with tube ageing, and other internal differences in count rates caused by downcore changes in matrix density, water and organic content (assessed using loss-on-ignition at 550 °C for 4 hours^46,47^), the raw cps data were normalised by incoherent scatter^24^, converted to percentages of total element and scatter cps sum (% cps), and centred log ratio (clr) transformed. Covariance, correlation and closed-sum effects were assessed on an individual site basis and then across all sites combined to investigate the non-stationarity (equifinality) of geochemical responses during deposition and through time^26^.

Elements in the (raw) cps dataset XRF-CS dataset were filtered, retaining only those elements with downcore signals that were statistically different from noise. Noisy elements were determined using two methods; first, a simple signal-to-noise ratios (SNR = µ/σ) threshold of > 2 (equivalent to > 95% significance) was calculated for each element on a site-by-site basis and then across the combined dataset. Signal-to-noise ratio (SNR) element filtering performed relatively well for individual sites, identifying elements with downcore profiles different from noise, but poorly across the combined dataset, with only Ni, Cu, Br, Zr, and incoherent and coherent scatter returning SNR values > 2. This is probably due to the large variability in counts produced from the peat matrix (with very low elemental counts) compared to the sporadic nature of mineral deposition (with high to very high elemental counts). Therefore, we applied an additional autocorrelation filtering process, based on the acf function in R and as part of the *itrax.R*^33^ quality control data processing procedure. A conservative ‘minimum’ correlation threshold of 0.1 and a more stringent elevated ‘maximum’ correlation threshold of 0.5 were both tested, using a conservative lag of 20 XRF-CS measurements (i.e., ~ 2 cm interval lag) that corresponded to twice the mean ICP-MS subsample thickness of ~ 1 cm; Supplementary Table S8). We assessed the measurement errors for XRF-CS using a combination of repeat measurements from duplicate scans of a similar sub-Antarctic peat matrix, producing mean measurement errors of < 5% for Ca, Ti, Fe, Sr and consistently high count rates for the six ‘key’ elements (Ca, Ti, Mn, Fe, Sr, Zr) used in multivariate analysis (Supplementary Fig. S2, Supplementary Table S3).

### Subsampling and discrete analyses

Following XRF-CS, cores were frozen and sliced at ~ 1 cm intervals using a stainless-steel band saw, divided into three sub-samples (for density/geochemistry, dating/macrofossil and archive) using ceramic knives and Teflon boards, and stored frozen in plastic Ziplock bags^48^. Water content and dry bulk density were calculated by weighing samples before and after freeze-drying, and by measuring the dimensions of each fresh density/geochemistry sample using a vernier calliper^48^.

Subsamples for ICP-MS were prepared following the method in Le Roux and De Vleeschouwer^8^. Dry samples were homogenised in 15–50 ml falcon digitubes with eight glass beads of 4 mm using a FastPrep^®^−24 – (3 × 20 s at 6 m s^− 1^) and then digested in a class 100 clean room at CRBE (Toulouse, France) following an established protocol whereby 100 mg of bulk sample was digested in Teflon vials on hot plates using an ultrapure HNO_3_-HF mixture (Optima™ ThermoFisher) at 110 °C for 48 h followed by evaporation steps (50 °C) and re-digestion using an HNO_3_-HCl mixture if required^9^. Samples were then evaporated, 2 ml of HNO_3_ were added and following proper dilutions for ICP-MS, measurements were performed using ultrapure water (18.2MΩ-cmELGA LabWater, Veolia). Measurements were either done on a quadrupole ICP-MS equipped with a collision cell (Agilent Technologies 7500ce, Toulouse, France) or a Triple Quadrupole ICP-MS (iCap TQ, Thermoscientific*)* at *Observatoire Midi Pyrénées* (Toulouse, France). The ICP-MS instruments were calibrated using a synthetic multi-element standard while an InRe solution was used as an internal standard. Procedural blanks were regularly measured and showed negligible values.

Blanks and several organic Certified Reference Materials (CRM) were run to assess accuracy and reproducibility for each element. The CRM were selected to match the composition and concentration range from pure organic material towards a mix between organic and mineral matrix: NIST-1547a (peach leaves), NIST-1515 (apple leaves), GBW-07603 (bush branches and leaves), IPE-176 (*Phragmites communis*) NJV-94-1 (peat). The accuracy represents how close the measured value is from the CRM value (i.e., the smaller the value the more accurate), and is calculated using the following formula:

$$\:Accuracy\:\left(\%\right)=\:\frac{{({C}_{CRM}-{C}_{meas})}^{2}}{{C}_{CRM}}\times\:100$$

where C_CRM_ is the concentration in the CRM, and C_meas_ is the average concentration on repeated measurements of the CRM.

Reproducibility represents how close *n* measurements are from each other (i.e., the larger the value, the more reproducible) and is calculated using the following formula:

$$\:Reproducibility\:\left(\%\right)=\frac{{C}_{meas}-{Stdev}_{meas}}{{Stdev}_{meas}}\times\:100$$

where Stdev is the standard deviation on repeated measurements of the CRM, and C_meas_ is the average concentration on repeated measurements of the CRM.

Overall accuracies range between 2% and 19%, except for Ni which shows a significantly higher value (54%) (Supplementary Table S1). Overall reproducibility was good, varying between 74% and 84% except for Ni and Zn which displayed reproducibility values of 64% and 65%, respectively. It is noticeable that the measured CRM does not report any value for Zr. Nevertheless, Zr was kept in the intercalibration exercise as it is an important element for possible dust flux reconstructions.

### Dataset matching

Depth matching was undertaken in itrax.R using the cps, log-normalised and clr datasets. The matched dataset represents the mean ± 1σ XRF-CS value across the minimum to maximum depth range of the ICP-MS subsample, with errors were assessed using a standard ANOVA (Analysis of Variance). The sporadic nature of (wind-driven) mineral deposition at our study sites and the process of matching mm-scale XRF-CS spectra to cm-scale ICP-MS subsample data for the XRF-CS dataset can create significant variability and large standard deviation values in the matched-dataset for XRF-CS. Even so, matched XRF-CS standard error values for the six ‘key elements’ were small (< 10%) due to high count rates for these elements and because the composite depth-matched sampling populations are large (i.e., n = 268, each comprising at least 10 XRF-CS spectra measurements) (Supplementary Table S2).

### Calibration screening and univariate regression

Pearson correlation analysis was first applied to identify elements exhibiting systematic covariance between XRF-CS and ICP-MS datasets (Fig. 2; Supplementary Fig. S2). Univariate calibration was then performed using OLS and WLS regression, including residual-weighted and inverse-variance weighted variants. Analytical uncertainty was propagated using measured standard deviations for ICP-MS where available and depth-matching. Standard model diagnostics (Shapiro-Wilk test, Cook’s distance and leverage) were assessed using the *performance.R* package^49^, with rejection thresholds set at twice the mean influence distance. Model prediction performance was evaluated using R², adjusted R², RMSE, RMSEP, Akaike and Bayesian Information Criteria (AIC, BIC for assessing overfitting^50^, and Breusch–Pagan tests for heteroscedasticity (i.e., non-constant or non-linear variance that requires weighting in univariate regression analysis). Of the original 12 matched-elements, only six elements (Ca, Ti, Fe, Mn, Sr, and Zr) that were consistently covariant with Ti and Zr were retained for multivariate analysis (Supplementary Methods for details).

### Multivariate calibration models

Four univariate models (OLS, inverse-variance weighted OLS, residual-weighted OLS, OLS_wt_, and inverse-variance WLS, WLS_wt_) were compared with four multivariate calibration approaches: (1) Bayesian generalised linear model (glm) using the *bayesglm* function in the *arm.R* package with default Gaussian priors and MAP (maximum posterior distributions, rather than the more time- and computationally intensive MCMC sampling)^51^; (2) Random Forest (RF) regression using the *randomForest.R* package using the default number of trees (ntree = 300) and default out-of-bag (OOB) diagnostics to assess regression model performance and generate errors^52^; (3) and (4) Two Partial Least Squares (PLS) regression models using *Leave-One-Out* (LOO) and 10-fold cross-validation (CV) using the *pls.R* package and its *predict* function for back-conversion from log-space to concentration-space^53^(Supplementary Table S10 for details).

Ten-fold cross-validation was applied to all models (except PLS-LOO) and R², adjusted R², RMSE, RMSEP (cross-validated and bootstrapped), residual normality (Shapiro–Wilk), and heteroscedasticity (Breusch–Pagan) were assessed where applicable. As for univariate models, influence and component tests (Cook’s distance, leverage, and standardised residuals, AIC and BIC) were used to identify influential observations that may affect model stability, including overfitting. AIC and BIC were not used in model ranking or classification schemes because they are not available for all model types (e.g., RF and PLS) (Supplementary Table S5, S6).

### Bootstrap validation and prediction uncertainty

Model robustness was evaluated using bootstrap resampling of the calibration dataset (2000 iterations, with RF capped at 500 for computational speed and stability and random 70:30 training–test splits). Bootstrap distributions and variance of R², RMSE, and RMSEP were initially used to assess model stability and prediction performance (Supplementary Methods for details). Predictions were generated for both calibration-matched (*n* = 268) and the XRF-CS (n = > 14k) datasets, de-centred where applicable and back-transformed into concentration units (mg kg⁻¹). Prediction uncertainty was quantified using three complementary approaches: (i) log-space 95% prediction intervals propagated to concentration space, (ii) log-normal multiplicative confidence bands derived from residual variance, and (iii) bootstrap predictive intervals from repeated refitting and prediction.

### Robustness and calibration model classification

Mirroring approaches adopted by previous geochemical, palaeoclimate, and palaeo-reconstruction studies to assess downcore predictions^35,36,40^, we applied a simple Signal-to-noise ratios (SNR)-based assessment, similar to XRF-CS element filtering. Signal-to-noise ratios (SNR) (mean prediction value/RMSE value, in concentration space), SNR prediction uncertainty (SNR_CI_), and downcore smoothness (SNR_smooth_), i.e., the difference between adjacent predicted 95% confidence interval widths, were calculated and rescaled as within element unit indices (i.e., 0–1) (Supplementary Eqs. 1–4 for details). To rank the predictive performance of the calibration models, these parameters were combined with R^2^ values into weighted robustness scores (within element unit indices) across all sites combined (globally) and per site (Supplementary Eqs. 5, 6, Supplementary Methods for details). Robustness scores and upper/lower quartile distribution thresholds were then used to classify within element model predictions into one of four comparative SNR signal types for each element as: Signal-dominated (> 0.75), Balanced (0.5–0.75), Uncertainty-dominated (0.3–0.5), and Noise-dominated (< 0.25) (Supplementary Eqs. 7–10).

Overall, we used a combination of calibration performance based metrics (R^2^, RMSE, RMSEP_CV_, RMSEP_boot_ ± σ) and robustness scores generated from SNR-based predictions metrics (SNR, SNR_CI_, SNR_smooth_) alongside these prediction classifications to assess which calibration model provided the best fit to ICP-MS data and minimises downcore (and temporal) prediction variability best (Fig. 4, Supplementary Fig. S13–S17, Supplementary Table S5). By combining established cross validation and bootstrapping statistical results (RMSEP mean ± σ; Supplementary Methods for details) with quantified element-specific prediction assessments, it was possible to distinguish between calibration methods even when differences in traditional calibration model performance metrics (i.e., R^2^, RMSE, RMSEP) were marginal. This also allowed us to identify models whose predictions did not appear to be visually reliable reconstructions of palaeoenvironmentally-driven change preserved in peat (e.g., RF for Ti and Zr at most sites in 6- and 4-element model runs; Supplementary Fig. S13-17).

The four-element (Ca, Ti, Sr, Zr) multivariate calibration models consistently outperformed univariate linear regression models, reflecting their ability to deal with covariance and non-constant variance, with PLS models providing the most stable agreement with measured ICP-MS concentrations, despite occasionally lower R² or higher RMSE/RMSEP values than Bayesian or RF models (Fig. 3; Supplementary Table S5b, d).

### Final calibration model

Following these calibration model tests, we developed bespoke cross-validated PLS calibration models, optimised for Ti- and Zr-prediction using the *pls.R* package^44^. This final calibration model was mean-centred and constrained to an optimum number of components (ncomp = 4; Ca, Ti, Sr, Zr) to minimise overfitting, with the four elements included selected by further cross-validation and jackknifing tests (Supplementary Methods, Supplementary Boxes 1–4). Predictive uncertainty was again quantified using 10-fold and LOO cross-validation and bootstrap resampling, and additional tests undertaken as previously described above. Overall, the final Ti- and Zr-prediction optimised PLS model improved R²_CV_ and reduced RMSE and RMSEP_cv/boot_ relative to univariate and multivariate calibration models with six components (Fig. 3; Supplementary Boxes 1–4, Figs. S13–S17, Table S5; Supplementary Methods, Results for details).

## Supplementary Information

Below is the link to the electronic supplementary material.

Supplementary Material 1

## Data Availability

The original contributions presented in the study are included in the article/Supplementary Information. Geochemical datasets for each site have been deposited with the NERC UK Polar Data Centre (UK-PDC) [https://data.bas.ac.uk](https:/data.bas.ac.uk) – see Supplementary Information for individual dataset doi’s. All R packages used, datafiles imported, and links to R code are referenced in the Supplementary Information and Supplementary Table S10.
