# Supplementary material for: High-resolution XRF-CS/ICP-MS mineral element data calibration and potential applications in sub-Antarctic peat records
Source: Sci Rep. 2026 Feb 26;16:8909. doi: 10.1038/s41598-026-41047-8 (PMC12988183; doi:10.1038/s41598-026-41047-8)
Supplement: Supplementary file 1 — Supplementary Material 1 [file 41598_2026_41047_MOESM1_ESM.pdf]

Supplementary Information

High-resolution XRF-CS / ICP-MS mineral element data calibration and potential applications in sub-Antarctic peat records

François De Vleeschouwer[1], Stephen J. Roberts[2*], Gaël Le Roux[3], Thomas Bishop[4], Sarah J. Davies[5], Angela Gallego-Sala[6], Charlotte Green[2], Bianca Perren[2], Krystyna M. Saunders[7], Alex Whittle[2,6], Anjali L. Dhunna[2,8], and Dominic A. Hodgson[2]

[1]Instituto Franco-Argentino para el Estudio del Clima y sus Impactos (IRL IFAECI/CNRS-CONICET-IRD-UBA), Dpto. de Ciencias de la Atmosfera y los Oceanos, FCEN, Universidad de Buenos Aires, Intendente Guiraldes 2160, Ciudad Universitaria, Pabellon II - 2do. Piso (C1428EGA) Ciudad Autonoma de Buenos Aires, Argentina.
[2]British Antarctic Survey (BAS), Natural Environmental Research Council (NERC), High Cross, Madingley Road, Cambridge, CB3 0ET, UK.
[3]Centre de Recherche sur la Biodiversité et l'Environnement (CRBE), CNRS, Université de Toulouse, IRD, Toulouse INP, Toulouse, Av. de l'Agrobiopôle, 31326 Auzeville-Tolosane, France.
[4]Department of Geography, Arthur Lewis Building, University of Manchester, Oxford Road, Manchester M13 9PL, UK.
[5]Geography and Earth Sciences, Llandinam Building, Penglais Campus, Aberystwyth University, SY23 3DB, UK.
[6]Geography, Laver Building, University of Exeter, North Park Road, Exeter, EX4 4QE, UK
[7]Institute for Antarctic and Marine Studies, University of Tasmania, Hobart, Tasmania, 7004, Australia.
[8]Department of Geography, Royal Holloway, University of London, Egham, Surrey, TW20 0EX, UK.

* Corresponding author: sjro@bas.ac.uk

Abstract

Peatlands are important environmental archives and mineral dust trapped in peat cores from multiple sites can be used to track past changes in hemispheric and global wind circulation patterns. X-ray Fluorescence Core Scanning (XRF-CS) can rapidly geochemically characterise minerals deposited in peat at sub-millimetre-resolution, but calibration is needed to obtain quantitative data. Here, we present a unique calibration of >14,000 contiguous mm-scale XRF-CS measurements depth-matched to 268 interval-based cm-scale Inductively Coupled Plasma Mass Spectrometry (ICP-MS) quantitative measurements from five peat records located on the west coasts of four sub-Antarctic Islands impacted by the Southern Hemisphere Westerly Winds. Of eight calibration models tested, a four-element (Ca, Ti, Sr, Zr) multivariate partial least squares (PLS) model optimised for the widely used dust flux elements Ti and Zr accounts for covariance and provides the most reliable predicted XRF-CS concentrations for Ti ($R^2_{CV} = 0.76$, $RMSEP_{boot} = 2203 \pm 705$ mg kg^{-1}, $R^2_{pred.} = 0.87$, $RMSE_{pred.} = 2136$ mg kg^{-1}, $P < 0.0001$). Predictions for Zr are indicative due to low Zr concentrations, but calibrated Ti and Zr XRF-CS concentrations align well with ICP-MS Ti and Zr concentrations for all five peatland sites. Our multivariate calibration protocol expands the scope of quantitative high-resolution palaeoenvironmental and geochemical research potentially to decadal–centennial timescales for peat records from, and beyond, the sub-Antarctic Islands.

Supplementary Methods

Bootstrap Validation and Prediction Uncertainty

Model robustness was evaluated using 2000 bootstrap resamples of the calibration dataset (except for RF models which were resampled 500 times to reduce computational complexity and prevent runaway bootstraps across tree structures). A 70:30 training–test dataset split was used for each element–model combination. Out-of-sample R^2 distributions were used to assess stability, with no models flagged as bootstrap-unstable (i.e., where neither the interquartile nor 5th-95th percentile range of R^2 values spanned zero), indicating consistent uncertainty-dominated predictive behaviour in all models. Bootstrapped resampling methods were used to derive means, standard deviations, and percentile-based confidence intervals, following established resampling and model validation and prediction methods[1–3].

Model performance was evaluated and ranked using a 10-fold cross-validated coefficient of determination (sum-of-squares R^2_{CV}) (except for the PLS-LOO, *Leave-One-Out*, model) and adjusted R^2 (sum-of-squares $R^2_{adj.}$) values (for linear models, higher is better), alongside mean squared error (MSE), RMSE, and overall prediction RMSEP values derived from 10-fold cross-validation ($RMSE_{cv}$), where possible, and bootstrap resampling methods ($RMSEP_{\beta}$), for all models (lower is better). These parameters allowed us to: (i) compare the proportion of variance in the ICP-MS reference data explained by each model; (ii) assess whether increasing the calibration model complexity improves its predictive ability; (e.g., univariate vs. multivariate models, PLS vs. RF models); (iii) assess overfitting (e.g., for univariate where $R^2_{adj} >> R^2_{CV}$). Together, these metrics provide an unbiased estimate of which models are most generally applicable to each downcore ICP-MS data profile for the five sites examined.

External validation was performed by applying calibrated models to two independent XRF-CS datasets: 1) depth-matched with the ICP-MS subsample dataset (n = 268) and 2) the n = >14k XRF-CS dataset, with predictions decentred and back transformed to concentrations (in mg kg^{-1}) to allow better interpretation. Predicted versus observed relationships were assessed using biplots, with horizontal error bars representing analytical uncertainty in observed ICP-MS concentrations and vertical error bars representing model-derived 95% prediction interval as shown in Supplementary Figures S9, S10 only.

Predictions were generated by inputting the XRF-CS element data and using exponential back-conversion and decentering functions embedded within each package listed or within R, using defaults to produce quantitative concentrations (in mg kg^{-1}) and 95% confidence intervals (95% CI) for all eight models (Supplementary Fig. S9b, S10b). Prediction uncertainty and confidence intervals were quantified in three ways using: (i) conventional log-space 95% prediction confidence intervals transformed to concentration errors and RMSEP values and uncertainties; (ii) log-normal multiplicative confidence intervals derived from calibration residual variance, expressed as percent uncertainty, then applied to new predictions; (iii) bootstrap predictive confidence intervals obtained from repeated model refitting and prediction resampling. Output prediction datasets are available via the Github data link shown in Supplementary Table S10.

Ten-fold cross validation calibration model and prediction performance and signal-to-noise ratio (SNR) prediction stability tests were used in combination with bootstrapped prediction errors to determine the homogeneity and distributions of overall variance (RMSE, $RMSE_{boot}$, RMSEP) and for 95% confidence intervals for each of the >14k predicted XRF-CS concentrations. Predictive uncertainty was quantified using the root-mean-square error in concentration space, estimated by cross-validated log-space prediction errors where available, but otherwise by bootstrap resampling (2000 iterations, except RF, which had 500 iterations to reduce computation run time; 70:30 training:test dataset). The $RMSEP_{boot}$ mean ± standard deviation was calculated for summarising

and comparing prediction performance between calibration models. Downcore predictions and the variance of the predicted XRF-CS concentrations were compared to the downcore ICP-MS profiles and each calibration model's performance (robustness) was then ranked, overall and for each site, using a simple signal-to-noise ratio based assessment (Supplementary Table S5).

Signal to Noise Ratio (SNR) Stability and Robustness Model Ranking

Performance metrics, prediction outputs, and model rankings produced by the 6-element (Ca, Ti, Fe, Mn, Sr, Zr) calibration model runs were compared with 4-element (Ca, Ti, Sr, Zr) runs. Four-element runs were optimised from dust flux elements Ti and Zr based on the improved performance observed in the PLS-LOO and PLS-CV models run with additional jackknifing component significance tests when only Ca, Ti, Sr, Zr tests. In summary, the initial 6-element model comparison exercise suggested that PLS models provided the best fit of predicted XRF-CS to measured ICP-MS data for Ti and Zr, despite sometimes having slightly lower R^2 and higher RMSE/RMSEP values than Bayesian and Random Forest models. Overall, multivariate methods (Bayes, RF, PLS k-fold, PLS-LOO) were ranked consistently better than ordinary least squares univariate methods (OLS, WLS and weighted OLS, WLS), reflecting better handling of covariance and heteroscedasticity for multivariate geochemical datasets.

The overall performance of each calibration model performance was then evaluated for 6-elements (Ca, Ti, Fe, Mn, Sr, Zr) and 4-elements (Ca, Ti, Sr, Zr) using a composite 'Robustness score', derived from a unit scaled (0-1) signal-to-noise ratio (SNR) and SNR confidence-based confidence interval indices of predicted XRF-CS downcore profiles (Supplementary Equations S1–S3). The prediction SNR (mean prediction value / RMSE in concentration space) (Equation S1) and prediction uncertainty (σ) (i.e., standard deviation of the predicted values) was calculated for each element, for all sites (global) and for each site. Prediction confidence interval based signal-to-noise ratios (SNR_{CI}) were then calculated for each XRF-CS calibrated prediction to quantify the variability in the predicted signal relative to its mean predicted uncertainty (i.e., its mean downcore 95% CI width, $U_{95} - L_{95}$ in Supplementary Equation S2). Within element relative downcore coherence of the predictions was then assessed using SNR smoothness (SNR_{smooth}), a signal-to-noise ratio derived from the inverse standard deviation of first-order differences in adjacent downcore XRF-CS predictions. This parameter represents the difference between adjacent predicted SNR confidence intervals downcore. Then SNR_{CI} , SNR_{smooth}, and R^2_{CV} were linearly rescaled (i.e., indexed from 0 to 1) to form SNR_{CI_sc} , SNR_{smooth_sc}, and $R^2_{CV\_sc}$. Values (Supplementary Equation S4, Supplementary Table S5)

Equation S1:

$$\mathrm{SNR_{model}} = \frac{\overline{\text{Mean Pred}(y)_{\text{ conc.}}}}{\mathrm{RMSE_{conc.}}}$$

Equation S2:

$$\mathrm{SNR_{CI}} = \frac{\sigma\left(\widehat{Mean\ Pred.}\right)}{\overline{(U_{95} - L_{95})}}$$

Equation S3:

$$\mathrm{SNR_{smooth}} = \frac{\mathrm{sd}(\hat{y})}{\mathrm{sd}(\Delta\hat{y})}$$

Equation S4:

$$Index\ scaled\ (\_sc)\ x^* = \frac{x - \min(x)}{\max(x) - \min(x)}$$

Prediction SNR smoothness (SNR_{smooth}) and SNR confidence intervals (SNR_{CI}) were combined with R^2 values into scaled indices (0-1), to ensure comparability across models for the same element for all sites and at each site.

Equation S5:

$$Robustness_{global} = 0.4\,\widetilde{\mathrm{SNR}_{\mathrm{CI\_sc}}} + 0.3\,\widetilde{\mathrm{SNR}_{\mathrm{smooth\_sc}}} + 0.3\,\widetilde{R^2}$$

Scaled indices were then used to calculate a weighted overall calibration model robustness score for all sites combined (Supplementary Equation S5). Splitting the weights as 0.4, 0.3, 0.3 favours predictive signal performance over statistical explanatory power, avoiding dominance by any single metric. The R^2 values remain meaningful and influence the overall calibration model rankings but they cannot dominate the Robustness score alone because they are always <50% of the total Robustness score – meaning that R^2 can be outranked by two SNR based parameters combined (up to 70% of total). This was set up in this manner because these two parameters relate more to predictive ability and reliability for the new >14k XRF-CS dataset rather than simply relying on calibration model performance.

For each site individually, $R^2_{CV\_sc}$ was omitted from the Robustness index because the calibration model is only applicable across all sites when combined (Supplementary Equation S6).

Equation S6:

$$Robustness_{per\ site} = rank\ (0.6 \cdot \mathrm{SNR}_{\mathrm{CI,sc}} + 0.4 \cdot \mathrm{SNR}_{\mathrm{smooth\_sc}})$$

Global Robustness scores for all sites are defined from predictive reliability and, therefore, this was the primary constraint placed on our Calibration Model Ranking order (Supplementary Equation S7). $RMSEP_{boot}$ was used in the ranking scheme because not all univariate models produced single $RMSEP_{CV}$ values.

Equation S7:

$$\text{Calibration Model Ranking} = rank\left(\text{Robustness}_{global}, R^2, \text{RMSEP}_{\text{boot}}\right)$$

Model Classification Schemes

Assessing signal stability is a widely used concept in XRF-CS analysis, and recommended for palaeoclimate reconstruction and time series studies[4–8]. Instead of relying on calibration performance based metrics alone, the robustness, stability, signal type and confidence classification schemes outlined below provide a more diagnostic evaluation of uncertainty and downcore/temporal structure and a robust framework for calibration model selection and interpretation.

Two classifications, Stability and Signal Type, were derived from the SNR-based Global Robustness scores to assess overall model stability and whether downcore signals were comparatively 'noisy' for each element (Supplementary Equations S8, S9). These classification schemes are not an absolute measure of prediction stability for each site and each element, instead they provide a comparative tool for assessing which of the eight calibration models produced predictions that were most 'stable' and the least 'noisy' (Supplementary Table S5).

(a) Stability Classification

Models were classified for within element relative stability by applying upper and lower quartile-based thresholds of 0.75 and 0.25 set to represent statistically meaningful cut off points for variability within each element across all eight models at each site (Supplementary Equation S8).

We confirmed our results visually (Supplementary Figures S13–S17). To summarise, sites with Global Robustness scores above the 0.75 threshold tended to be smoother and had a more coherent stratigraphic structure (Supplementary Fig. S13–S17). Any models classified as 'Unstable' had downcore structure within the lower quartile of variability (and marginally above) and tended to have visually more 'noisy' predictions than 'Stable'. This can have a number of causes, including: (i) calibration model-generated overfitting (e.g., producing excessively noisy Random Forest downcore predicted Ti values for at BI10, HER42PB and KER1 in Supplementary Fig. S13, S14), and (ii) excessive prediction noise amplification, and/or poor calibration model uncertainty propagation. Models exhibiting unstable downcore behaviour were excluded from further Robustness Classifications.

Equation S8:

Stability classification

$$\text{Robustness}_{site} >= 0.75 \sim \text{Stable}$$
$$\text{Robustness}_{site} >= 0.50 \sim \text{Acceptable}$$
$$\text{Robustness}_{site} >= 0.30 \sim \text{Marginal}$$
$$\text{Robustness}_{site} <= 0.25 \sim \text{Unstable}$$

(b) Signal Type Classification

We used an additional assessment to distinguish between:

(i) 'Signal-dominated' with a Robustness_{site} index >0.75, i.e., above the upper quartile range value of 0.75 reflecting high SNR_{CI} and SNR_{smooth} indices. These models produced a coherent downcore signal, constrained uncertainty existed and bootstrap resampling indicated stable calibration.

(ii) 'Balanced' predictions occurred where neither Signal nor Uncertainty predictions were dominant, defined by a Robustness_{site} index between >0.5 and <0.75.

(iii) 'Uncertainty-dominated' predictions, with low signal-to-noise in either the SNR_{CI} or SNR_{smooth} parameters. These signals dominate downcore profiles that have predictions with visually higher variability and a Robustness_{site} index between 0.3 and 0.5, i.e., marginally above the lower quartile range threshold.

(iv) 'Noisy' predictions have very low signal-to-noise ratios and either SNR_{CI} or SNR_{smooth} parameters. Models classified as 'Noisy' have a Robustness_{site} index within the lower quartile range, scoring <0.25.

Equation S9:

Signal Type classification

$$\text{Signal-dominated} >= 0.75 \ \ (\text{Stable})$$
$$\text{Balanced} >= 0.50 <= 0.75 \ \ (\text{Acceptable})$$
$$\text{Uncertainty-dominated} >= 0.30 <= 0.50 \ (\text{Marginal})$$
$$\text{Noise} <= 0.25 \ (\text{Unstable})$$

(c) Confidence Classification

Our Confidence Classification scheme is similar to the Signal Type Classification scheme but uses wider threshold bands to provide a quick overall assessment confidence in the calibration model.

Equation S10:

$$\textbf{Confidence Classification}$$
$$\text{High} >= 0.67 \text{ Signal Dominated}$$
$$Medium > 0.33 \text{ Balanced \& Uncertainty Dominated}$$
$$\text{Low} <= 0.33 \text{ Uncertainty \& Noise Dominated}$$

Together, SNR-based Robustness scores and the classification scheme approach outlined above is a relatively quick and simple method for assessing whether machine learning and/or probabilistic calibration models (e.g., Random Forest) generated high-frequency variability in their predictions, which could be considered equivalent to analytical noise. Excessively 'noisy' predictions more likely reflect model artefacts and/or overfitting rather than being a genuine response to environmental change. The optimum smoothing interval can also be found using this approach and our suggested optimal sample interval for quantified XRF-CS predictions in peat records in this study is 0.5 cm.

Supplementary Results

Optimising the number of components for multivariate calibration

In Figure 2, and in Supplementary Figures S3–S5, comparisons between the cps, centred log ratio, where $clr_{element} = log_n$(element / Geometric mean of all 12 elements), i.e., K, Ca, Ti, Mn, Fe, Co, Ni, Cu, Zn, Rb, Sr, Zr), and log-space incoherence normalised, i.e., log_n(element cps / inc. cps), datasets highlight how the cps dataset does not account for changes density and water content in the XRF-CS data. In the cps matrix (Fig. 2a, Supplementary Fig. S3) the strong and positive relationships between Ti, Ca, Fe, Mn, and Sr XRF-CS and with their ICP-MS equivalents (labelled ICP) are potentially misleading. The centred-log-ratio (clr) matrix is fundamentally different to the cps (and log-normalised) matrix because it examines inter-element log-ratios against the mean of all the elements, rather than absolute abundance (of counts, in the case of cps). Therefore, clr transformation accounts for differences in water content (density) in the closed sum XRF-CS cps dataset, and when comparing XRF-CS to ICP-MS data; hence, clr can identify more subtle environmental shifts and geochemical influences that alter relationships between elements, for example, the presence of carbonates (Ca, Sr), guano input (Fe, Cu, Zn), redox-sensitivity (Mn, Fe), and grain size variations (Ti, Fe, Zr).

In the clr matrix (Fig. 2a, Supplementary Fig. S4), the relationships between Ca-Sr ICP (0.8), Ca-Sr XRF-CS (0.6) are stronger and more positive than Ti-Ca ICP (0.24), Ti-Ca XRF-CS (0.38) and Ti-Sr ICP (-0.08), Ti-Sr XRF-CS (0.27), suggesting a secondary, potentially carbonate source for Ca and Sr (e.g., wind-blown shells). Additionally, Mn and Fe, which can be indicators of changing redox-sensitivity and can be mobile in peat, are weakly or negatively correlated to practically everything else, except for their own ICP-MS and XRF-CS correlations. Ti, Fe, Zr often track fine to coarse terrigenous and/or dust inputs. However, Zr is weakly or negatively correlated to almost everything, including Ti XRF-CS and Fe ICP and XRF-CS, except for Ti-Zr ICP (0.61) and Ti XRF-CS-Zr ICP (0.54). This suggests, primarily, that Zr is tracking changes in grain size (across the dataset as a whole), while the mostly negative relationship between Ti and Fe (0.01 to -0.51), and between Fe and Ca, Sr for ICP-MS and XRF-CS (-0.38 to -0.59) implies other sources of (biogenic) Fe (e.g., guano), unrelated to fine grain-terrigenous and/or carbonate inputs, at least for some sites, e.g. BI10 on Bird Island (Fig. 2).

The log-space, incoherent normalised matrix (Fig. 2a, Supplementary Fig. S3) accounts for changes in water content, but not density or grain size-related artefacts in the cps dataset. While this

transformation restores the mostly positive correlations of the cps matrix, it potentially masks environmentally driven changes in secondary sources for Ca, Sr, Mn and Fe. Nevertheless, reductions in the strength of the correlations between Ti and Ca, Sr and Ti and Fe, Mn are consistent with changes in correlation between the cps and clr matrices, also suggesting a secondary source for Ca, Sr, Mn and Fe. The simple correlation results highlighted the need for a multivariate calibration model optimised for terrigenous and/or dust elements Ti and Zr.

To develop the final PLS model optimised for Ti and Zr prediction, we used Variable Importance in Projection (VIP) scores on the log-space calibration training set (n = 268) for six key elements (Ti, Ca, Mn, Fe, Sr, and Zr) and four elements (Ti, Ca, Sr, Zr). We also used jackknifing, a standard and widely used statistical resampling method, to assess the stability of the final PLS model, to determine which predictor variables have the most significant influence on the PLS calibration model's outcomes. Variable Importance in Projection scores are first-look diagnostic test used in PLS regression used to quantify how important each predictor variable is in explaining the response across the latent components (or elements) in the model. VIP scores combines information about how strongly the variable loads onto each PLS component, showing how much variance in the response variable each component explains. This helped identify the optimum number of components for the PLS calibration model.

VIP values for ncomp = 6

Ti	Ca	Fe	Mn	Sr	Zr
2.344	0.471	0.399	0.256	0.145	0.195

VIP values ncomp = 4

Ti	Ca	Sr	Zr
1.936	0.431	0.116	0.226

VIP > 1: Important — strong contribution to the model
0.8 < VIP < 1: Moderate contribution
VIP < 0.8: Unimportant — likely not useful for prediction

The VIP scores for Ti were inconclusive because they only highlight Ti as a strong influence for Ti predcitions. They show that univariate models likely perform similar to a multivariate PLS model for 6 or 4 components (elements), which is also shown by the similarity of R^2 performance metrics for univariate and multivariate models in our calibration model tests.

Cross validation with jackknifing tests provided a more robust way to determine which components (elements) have the most influence on predictions produced for each element. In contrast to the VIP results, our jackknifing results showed that Ti, Ca, Sr and Zr consistently had the most significant influence on Ti predictions (Boxes 1 – 4) and that Ti, Ca, and Zr had most significant influence on Zr predictions.

In six component tests (Box 1, 3), Fe also had a very a significant influence on Ti, as it was highly covariant with Ti, but had minimal influence on Zr, with no covariance. These findings match the results of our initial correlation and co-variance tests (Figure 2) and is the main reason why Fe and Mn were left out of the final 4-element PLS calibration model optimised for Ti and Zr (dust-flux) prediction. Fe and Mn have multiple sources in peat aside from minerogenic deposits, including non-minerogenic redox reactions – and, as such, both these elements have been shown to have limited interpretation potential in peat. Retaining Fe and Mn when reconstructing 'dust' fluxes potentially destabilises Ti- and Zr- predictions even though the impact on calibration model performance metrics (i.e., R^2, RMSE) was not obvious or particularly significant.

This is, perhaps, one reason why the 4-element models produced predictions with a better fit to measured Ti ICP-MS values, and why it also had reduced uncertainty and a smaller 95% confidence interval width than some of the 6-element multivariate calibration models (Supplementary Figures S13–S17, Supplementary Table 5).

Overall, the two preliminary PLS calibration models run with four elements (Ca, Ti, Sr, Zr) proved to be the most robust multivariate models for predicting quantitative concentrations (in mg kg^{-1}) for Ti and Zr from more than 14,000 sampling points in the quality-controlled XRF-CS dataset across all five peatlands sites (8-model, 4-element runs: Ti: $R^2_{CV/LOO}$ (Sum of Squares) = 0.76, R^2_{pred} = 0.87, $RMSEP_{boot}$ = 2203 ± 705 mg kg^{-1}, P<0.0001; Zr: $R^2_{CV/LOO}$ = 0.72, R^2_{pred} = 0.60, RMSEP = 88.5 ± 34.9 mg kg^{-1}, P<0.0001; Supplementary Methods, Boxes 1–4 for details, Fig. S9–S17, Table S5). In the final four-element PLS model optimised Ti- and Zr-prediction (Fig. 3), Ti, Ca, and Sr were highly significant contributors at the p<0.001 level for Ti and Zr, but Sr had no significant influence on Zr (Supplementary Box 2, 4). Fe had a very significant or significant influence on Ti and Zr prediction (p<0.001 or p<0.01). Fe destabilised Ti and Zr predictions marginally, increasing the value of their downcore predictions and variability overall (PLS-kfold: 6-element Mean predicted value ± Mean 95% CI $width_{all-sites}$ Ti = 2282 ± 7490 mg kg^{-1} and Zr 49 ± 192 mg kg^{-1}; 4-element Ti = 2139 ± 6388 mg kg^{-1} and Zr = 44 ± 161 mg kg^{-1}), and creating a marginally poorer fit to measured ICP-MS data (Supplementary Fig. S14a, b, Supplementary Table 5a, c).

To summarise, we left Mn and Fe out of the final four element PLS calibration model because our correlation analysis suggested there were potential sources of Fe other than fine-grained terrigenous inputs in peat, including redox-sensitive reactions unrelated to minerogenic deposition. Fe also has XRF-CS counts on the Itrax XRF Core Scanner that are an order of magnitude higher than all other elements, and this appeared to destabilise the calibration. Nevertheless, there is a good fit between predicted and measured Fe and Mn values in the 6-element calibration model (Supplementary Fig. 10a) and between downcore predicted values and measured ICP-MS values for Fe and Mn across all sites (Supplementary Figs. S13a, S14a, S15a, S16a, S17a). Our analysis shows that optimising a PLS calibration model for Fe and Mn is possible if the aim of a follow-up study was, for example, examining high-resolution redox changes in peat records from XRF-CS data.

More fundamentally, the preliminary four-element multivariate calibration models produced predictions with higher SNR_{CI} values and (smoother) downcore (i.e., higher SNR_{smooth} values) – meaning they were (marginally) more stable and reliable overall (Supplementary Fig. S9, 10, Table S5b, d). Consequently, the 4-element calibration models also have better Global Robustness scores than the 6-element model runs and are, therefore, considered more reliable for predicting Ti and Zr. Predictions from RF models often far exceeded measured ICP-MS values and errors, even when their calibration model performance indicators outperformed multivariate PLS models. Yet, because measured and predicted concentrations were persistently low for Zr, a six-element Random Forest calibration model might ultimately prove to be more reliable for Zr dust flux reconstruction at some sites (Supplementary Fig. S13–S17). Boxes 1-4 (overpage) show summary k-fold and LOO cross validation (k=10), jackknifing, and variance explained validation tests performed on PLS models prior to reducing the number of elements included in multivariate models from six to four.

Boxes 1–4: Significance values

≤ p-value	Description
0.001	Highly significant (i.e., strong evidence against the null hypothesis that an element has no influence)
0.01	Very significant
0.05	Significant
0.1	Marginally significant
1	Not statistically significant

Box 1: 6-element Jackknife Optimum Component Influence Test for Ti

LOO CV leave-one-out segments; log-space; n= 268

ncomp_max:	(LOO)	6	
Optimal ncomp	(LOO)	5	
Min RMSEP	(LOO)	0.6137	log-space
VALIDATION:	RMSEP		

Cross-validated Jackknife test using 268 leave-one-out segments.

	(Intercept)	1	2	3	4	5	6
CV	1.2350	0.7215	0.6595	0.6328	0.6190	0.6137	0.6154
adjCV	1.2350	0.7215	0.6595	0.6328	0.6190	0.6136	0.6153

TRAINING: % variance explained

X	1	2	3	4	5	6
Y_test	77.94	89.17	92.96	96.39	98.07	100.00
NULL	66.16	72.26	75.40	76.84	76.90	76.91

Response

Y_test (5 comps)	Estimate	Std. Err.	Df	t-value	Pr(>|t|)	Signf.(p≤)
Ti	1.0550	0.1573	267	6.7062	0.0000	0.001
Ca	-0.5146	0.1577	267	-3.2639	0.0012	0.01
Mn	0.1273	0.1634	267	0.7793	0.4365	1
Fe	-0.1928	0.0701	267	-2.7500	0.0064	0.01
Sr	0.4679	0.1232	267	3.7987	0.0002	0.001
Zr	0.1298	0.0732	267	1.7726	0.0774	0.1

k-fold CV 10 segment, type: random; log space; n= 268

ncomp_max:	(k-fold)	6	
Optimal ncomp	(k-fold)	5	
Min RMSEP	(k-fold)	0.6110	log-space
VALIDATION:	RMSEP		

Cross-validated Jackknife test using 10 random segments.

	(Intercept)	1	2	3	4	5	6
CV	1.2350	0.7242	0.6615	0.6327	0.6183	0.6110	0.6125
adjCV	1.2350	0.7238	0.6609	0.6316	0.6166	0.6100	0.6114

TRAINING k-fold: % variance explained for Ti 6-element model (Ca, Ti, Fe, Mn, Sr, Zr)

	1	2	3	4	5	6
X	77.94	89.17	92.96	96.39	98.07	100.00
Y_test	66.16	72.26	75.40	76.84	76.90	76.91

Response

Y_test (5 comps)	Estimate	Std. Err.	Df	t-value	Pr(>|t|)	Signf.p≤
Ti	1.0550	0.1724	9	6.1208	0.0002	0.001
Ca	-0.5146	0.1372	9	-3.7500	0.0046	0.01
Mn	0.1273	0.1593	9	0.7992	0.4448	1
Fe	-0.1928	0.0343	9	-5.6268	0.0003	0.001
Sr	0.4679	0.0576	9	8.1279	0.0000	0.001
Zr	0.1298	0.0651	9	1.9943	0.0773	0.1

Summary Interpretation – only Sr, Ca, and Fe have a significant influence on Ti

- **Ti** has a very significant influence on Ti prediction ($p<0.001$) – **retained as a dust flux element**
- **Sr** has a very significant influence on Ti prediction ($p<0.001$) – **retained**
- **Ca** has a significant influence on Ti prediction ($p<0.01$) – **retained**
- **Zr** has a marginally significant influence on Ti prediction ($p<0.1$) - **retained as a dust flux element**
- **Fe** has a very significant or significant influence on Ti prediction ($p<0.001$ or $p<0.01$), but multiple sources in peat & high XRF-CS counts destabilised Ti calibration model predictions in tests – **not retained for Ti, Zr prediction**
- **Mn** has no statistically significant influence on Ti prediction, $p<1$ – **not retained**

Box 2: 4-element Jackknife Optimum Component Influence Test for Ti

LOO CV leave-one-out segments; log-space; n= 268

ncomp_max:	(LOO)	4	
Optimal ncomp	(LOO)	4	
Min RMSEP	(LOO)	0.6165	log-space
VALIDATION:	RMSEP		

Cross-validated Jackknife test using 268 leave-one-out segments.

	(Intercept)	1	2	3	4
CV	1.2350	0.6900	0.6627	0.6184	0.6165
adjCV	1.2350	0.6900	0.6627	0.6184	0.6165

TRAINING: % variance explained

X	1	2	3	4
Y_test	81.73	95.65	98.21	100.00
NULL	69.04	71.71	75.62	75.78

Response

Y_test (4 comps)	Estimate	Std. Err.	Df	t-value	Pr(>|t|)	Signf.(p≤)
Ti	1.0057	0.1309	267	7.6849	0.0000	0.001
Ca	-0.6097	0.1207	267	-5.0498	0.0000	0.001
Sr	0.5231	0.1102	267	4.7449	0.0000	0.001
Zr	0.2130	0.0745	267	2.8575	0.0046	0.01

k-fold CV 10 segment, type: random; log-space; n= 268

ncomp_max:	(k-fold)	4	
Optimal ncomp	(k-fold)	4	
Min RMSEP	(k-fold)	0.6163	log-space
VALIDATION:	RMSEP		

Cross-validated Jackknife test using 10 random segments.

	(Intercept)	1	2	3	4
CV	1.2350	0.6919	0.6656	0.6188	0.6163
adjCV	1.2350	0.6916	0.6651	0.6182	0.6157

TRAINING k-fold: % variance explained for Ti 6-element model (Ca, Ti, Fe, Mn, Sr, Zr)

	1	2	3	4
X	81.73	95.65	98.21	100.00
Y_test	69.04	71.71	75.62	75.78

Response

Y_test (4 comps)	Estimate	Std. Err.	Df	t-value	Pr(>|t|)	Signf.p≤
Ti	1.0057	0.1504	9	6.6860	0.0001	0.001
Ca	-0.6097	0.1173	9	-5.1990	0.0006	0.001
Sr	0.5231	0.0559	9	9.3570	0.0000	0.001
Zr	0.2130	0.0682	9	3.1251	0.0122	0.01

Summary Interpretation – all elements now have a significant influence on Ti

- **Sr** still has a very significant influence on Ti prediction ($p<0.001$) - **retained**
- **Ca** now has a very significant influence on Ti prediction ($p<0.001$) - **retained**
- **Zr** now has a significant influence on Ti prediction ($p<0.01$) - **retained as a dust flux element**

Box 3: 6-element Jackknife Optimum Component Influence Test for Zr

LOO CV leave-one-out segments; log-space; n= 268

ncomp_max:	(LOO)	6	
Optimal ncomp	(LOO)	5	
Min RMSEP	(LOO)	0.7234	log-space
VALIDATION:	RMSEP		

Cross-validated Jackknife test using 268 leave-one-out segments.

	(Intercept)	1	2	3	4	5	6
CV	1.3190	1.0280	0.7731	0.7525	0.7350	0.7234	0.7242
adjCV	1.3190	1.0280	0.7730	0.7525	0.7348	0.7233	0.7242

TRAINING: % variance explained

X	1	2	3	4	5	6
Y_test	76.39	89.39	94.10	96.31	98.03	100.00
NULL	39.64	66.69	69.73	72.24	72.34	72.35

Response

Y_test (5 comps)	Estimate	Std. Err.	Df	t-value	Pr(>|t|)	Signf.(p≤)
Ti	1.0562	0.1885	267	5.6041	0.0000	0.001
Ca	-1.0867	0.2088	267	-5.2056	0.0000	0.001
Mn	0.0850	0.1876	267	0.4530	0.6509	1
Fe	-0.0333	0.0826	267	-0.4035	0.6869	1
Sr	0.1528	0.2368	267	0.6453	0.5193	1
Zr	0.7438	0.1148	267	6.4770	0.0000	0.001

k-fold CV 10 segment, type: random; log space

ncomp_max:	(k-fold)	6	
Optimal ncomp	(k-fold)	5	
Min RMSEP	(k-fold)	0.7194	log-space
VALIDATION:	RMSEP		

Cross-validated Jackknife test using 10 random segments.

	(Intercept)	1	2	3	4	5	6
CV	1.3190	1.0270	0.7693	0.7463	0.7293	0.7194	0.7200
adjCV	1.3190	1.0270	0.7688	0.7456	0.7268	0.7178	0.7184

TRAINING k-fold: % variance explained for Ti 6-element model (Ca, Ti, Fe, Mn, Sr, Zr)

	1	2	3	4	5	6
X	76.39	89.39	94.10	96.31	98.03	100.00
Y_test	39.64	66.69	69.73	72.24	72.34	72.35

Response

Y_test (5 comps)	Estimate	Std. Err.	Df	t-value	Pr(>|t|)	Signf.p≤
Ti	1.0562	0.1850	9	5.7082	0.0003	0.001
Ca	-1.0867	0.2268	9	-4.7911	0.0010	0.001
Mn	0.0850	0.1646	9	0.5161	0.6182	1
Fe	-0.0333	0.0973	9	-0.3425	0.7398	1
Sr	0.1528	0.2032	9	0.7520	0.4713	1
Zr	0.7438	0.0940	9	7.9163	0.0000	0.001

Summary Interpretation – only Ti, Ca and Zr have a significant influence on Zr

- **Ti** has a very significant influence on Zr prediction ($p<0.001$) – **retained for Ti prediction**
- **Ca** has a very significant influence on Zr prediction ($p<0.001$) – **retained**
- **Zr** has a very significant influence on Zr prediction ($p<0.001$) - **retained as a dust flux element**
- **Mn, Fe, Sr** have no statistically significant influence on Zr prediction, $p<1$ – **Fe, Mn not retained, Sr retained for Ti**

Box 4: 4-element Jackknife Optimum Component Influence Test for Zr

LOO CV leave-one-out segments; log-space; n= 268

ncomp_max:	(LOO)	4	
Optimal ncomp	(LOO)	4	
Min RMSEP	(LOO)	0.7097	log-space
VALIDATION:	RMSEP		

Cross-validated Jackknife test using 268 leave-one-out segments.

	(Intercept)	1	2	3	4
CV	1.3190	0.9821	0.7649	0.7136	0.7097
adjCV	1.3190	0.9821	0.7649	0.7135	0.7097

TRAINING: % variance explained

X	1	2	3	4
Y_test	79.79	96.35	98.10	100.00
NULL	44.89	67.24	72.04	72.19

Response

Y_test (4 comps)	Estimate	Std. Err.	Df	t-value	Pr(>|t|)	Signf.(p≤)
Ti	1.0668	0.1385	267	7.7011	0.0000	0.001
Ca	-1.0873	0.1756	267	-6.1901	0.0000	0.001
Sr	0.1789	0.1899	267	0.9423	0.3469	1
Zr	0.7668	0.0963	267	7.9645	0.0000	0.001

k-fold CV 10 segment, type: random; log-space; n= 268

ncomp_max:	(k-fold)	4	
Optimal ncomp	(k-fold)	4	
Min RMSEP	(k-fold)	0.7170	log-space
VALIDATION:	RMSEP		

Cross-validated Jackknife test using 10 random segments.

	(Intercept)	1	2	3	4
CV	1.3190	0.9823	0.7728	0.7201	0.7170
adjCV	1.3190	0.9823	0.7717	0.7186	0.7157

TRAINING k-fold: % variance explained for Ti 6-element model (Ca, Ti, Fe, Mn, Sr, Zr)

	1	2	3	4
X	79.79	96.35	98.10	100.00
Y_test	44.89	67.24	72.04	72.19

Response

Y_test (4 comps)	Estimate	Std. Err.	Df	t-value	Pr(>|t|)	Signf.p≤
Ti	1.0668	0.1100	9	9.6953	0.0000	0.001
Ca	-1.0873	0.2222	9	-4.8920	0.0009	0.001
Sr	0.1789	0.2104	9	0.8504	0.4171	1
Zr	0.7668	0.0840	9	9.1259	0.0000	0.001

Summary Interpretation – only Ti, Ca and Zr have a significant influence on Zr

- **Ti** still has a very significant influence on Zr prediction (p<0.001) – **retained for Ti prediction**
- **Ca** still has a very significant influence on Zr prediction (p<0.001) – **retained**
- **Zr** still has a very significant influence on Zr prediction (p<0.001) - **retained as a dust flux element**
- **Sr** has no statistically significant influence on Zr prediction, p<1 – **retained for Ti prediction**

Calibration of clr datasets

For closed-sum geochemical datasets, multivariate models and clr data transformations are preferred for comparison of downcore elemental data because they account for covariability between elements[9,10]. Converting data to clr can highlight matrix particularities, but it is not considered suitable for calibration[9] because fully quantitative predictions require a mean composition for each new sample to be known a priori within the prediction dataset.

We tested both PLS calibration models using the clr ICP-MS – XRF-CS matched datasets, also with model predictive uncertainty quantified using 10-fold cross-validation, LOO and bootstrap resampling (n=1000) by splitting the whole dataset randomly 60:40 into new training and test datasets (Supplementary Table S8). For the clr PLS model, the *invclr* function in the *compositions.R* package[11] was used to generate the predicted clr matrix. To back-convert the de-centred clr PLS output matrix into Ti, Ca, Sr, and Zr concentrations (in mg kg^{-1}), each new sample point was multiplied by the mean total ICP-MS composition for each element. This process assumes that overall mean (geochemical) composition of samples in the new test dataset is similar to the training dataset. Mismatches between log-normalised PLS and clr PLS model downcore predictions would be expected if this clr back-conversion assumption was not valid and would suggest that the overall mean composition of the peat matrix at a site is heterogenous.

A consistently poor correlation and a lack of co-variance was clearly evident in the clr dataset between some elements such as Sr and Zr, (Fig. 2a, Supplementary Fig. S8). Nevertheless, four element PLS clr model predictions for Ti, Sr, Zr were generally within 5/95% confidence limits across all sites (except PB1). Our experimental calibration model runs using the clr dataset show that where the assumption that the matrix is 'homogenous' is even broadly met, concentrations of Ti calculated using the total mean composition, derived from the calibration training by a back-conversion process, were similar to those generated by log-normalised univariate and multivariate regression models (Supplementary Fig. S13).

Overall, XRF-CS predictions produced using the clr dataset from multivariate PLS calibration modelling implied that, overall, the mean geochemical composition of the peat matrix from BI10, HER42PB, KER1 and KER3 was similar to the mean composition of the XRF – ICP-MS training dataset used in PLS regression (Supplementary Table S4). The obvious exception was site PB1, which has a dense and highly minerogenic basal unit. Here, clr predictions of Ti significantly underestimated measured Ti concentrations (Supplementary Fig. S18n). This illustrates a limitation of our current dataset when applied to deposits with a higher mineral content (i.e., deposits that are technically not peat deposits).

Data and code availability

Data has been deposited in the NERC EDS UK Polar Data Centre (PDC) and can be accessed from the following doi links:

Roberts, S., De Vleeschouwer, F., Le Roux, G., Bishop, T., Davies, S., Gallego-Sala, A., Green, C., Perren, B., Saunders, K., Whittle, A., & Hodgson, D. (2025). Geochemical data from peat records collected from sub-Antarctic Isla Hermite, Site HER42PB_ (Version 1.0) [Data set]. NERC EDS UK Polar Data Centre. https://doi.org/10.5285/a0d0df71-f361-4967-a1dd-8dffdbf287b3

Roberts, S., De Vleeschouwer, F., Le Roux, G., Bishop, T., Davies, S., Gallego-Sala, A., Green, C., Perren, B., Saunders, K., Whittle, A., & Hodgson, D. (2025). Geochemical data from peat records collected from sub-Antarctic Bird Island, South Georgia, Site BI10_ (Version 1.0) [Data set]. NERC EDS UK Polar Data Centre. https://doi.org/10.5285/85ee0438-731e-4e51-8d77-53d0ec30a26f

Roberts, S., De Vleeschouwer, F., Le Roux, G., Bishop, T., Davies, S., Gallego-Sala, A., Green, C., Perren, B., Saunders, K., Whittle, A., & Hodgson, D. (2025). Geochemical data from peat records collected from sub-Antarctic Marion Island, Site PB1_ (Version 1.0) [Data set]. NERC EDS UK Polar Data Centre. https://doi.org/10.5285/931c6618-2755-4243-a0d6-f0d18f8ca9c6

Roberts, S., De Vleeschouwer, F., Le Roux, G., Bishop, T., Davies, S., Gallego-Sala, A., Green, C., Perren, B., Saunders, K., Whittle, A., & Hodgson, D. (2025). Geochemical data from peat records collected from sub-Antarctic Kerguelen Island, Site KER1_ (Version 1.0) [Data set]. NERC EDS UK Polar Data Centre. https://doi.org/10.5285/d712633e-a534-4605-aa34-cc4a8fe16107

Roberts, S., De Vleeschouwer, F., Le Roux, G., Bishop, T., Davies, S., Gallego-Sala, A., Green, C., Perren, B., Saunders, K., Whittle, A., & Hodgson, D. (2025). Geochemical data from peat records collected from sub-Antarctic Kerguelen Island, Site KER3_ (Version 1.0) [Data set]. NERC EDS UK Polar Data Centre. https://doi.org/10.5285/dc11db5d-7a00-4239-8d8e-858b833698ef

Datasets and R code used to analyse and plot all data in this study can be accessed here: https://github.com/stever60/ACE_peat_calibration

Itrax XRF-CS datafiles and spectra file outputs are available here: https://github.com/stever60/ITRAX_data

Packages used and specific files and folders for the calibration exercise and final PLS calibration model analysis are listed in Supplementary Table S10.

Supplementary Figure S1. Summary data analysis workflow and decision tree.

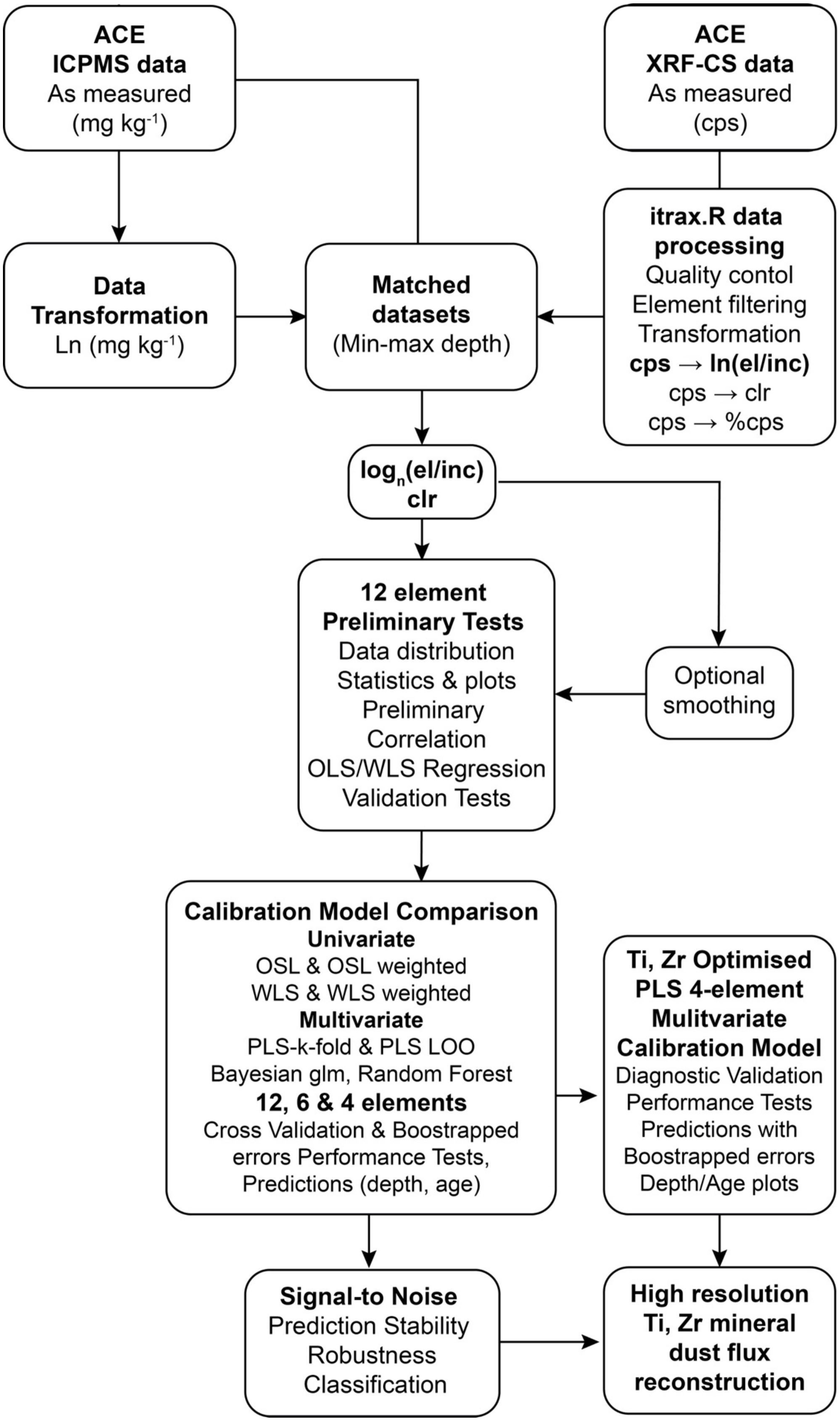

Supplementary Figure S2. Data distribution plots for the Ti in the matched XRF-CS and ICP-MS ACE dataset. **(a)** As measured cps XRF-CS data violin plots; **(b)** As measured ICP-MS (mg kg^{-1}) violin plots; **(c)** As measured ICP-MS Ti (mg kg^{-1}) and XRF-CS Ti cps biplot; **(d)** ICP-MS Ti and XRF-CS Ti centred log ratio (clr) biplot (12 element clr); **(e)** Repeat measurement error analysis of a 5 cm section from a 50 cm peat core (10% of total depth) from Marion Island highlighting typical reproducibility errors for the six key elements and scatter parameters from a similar peat matrix, highlighting that measurement errors in XRF-CS depend on count rate (n = 1150).

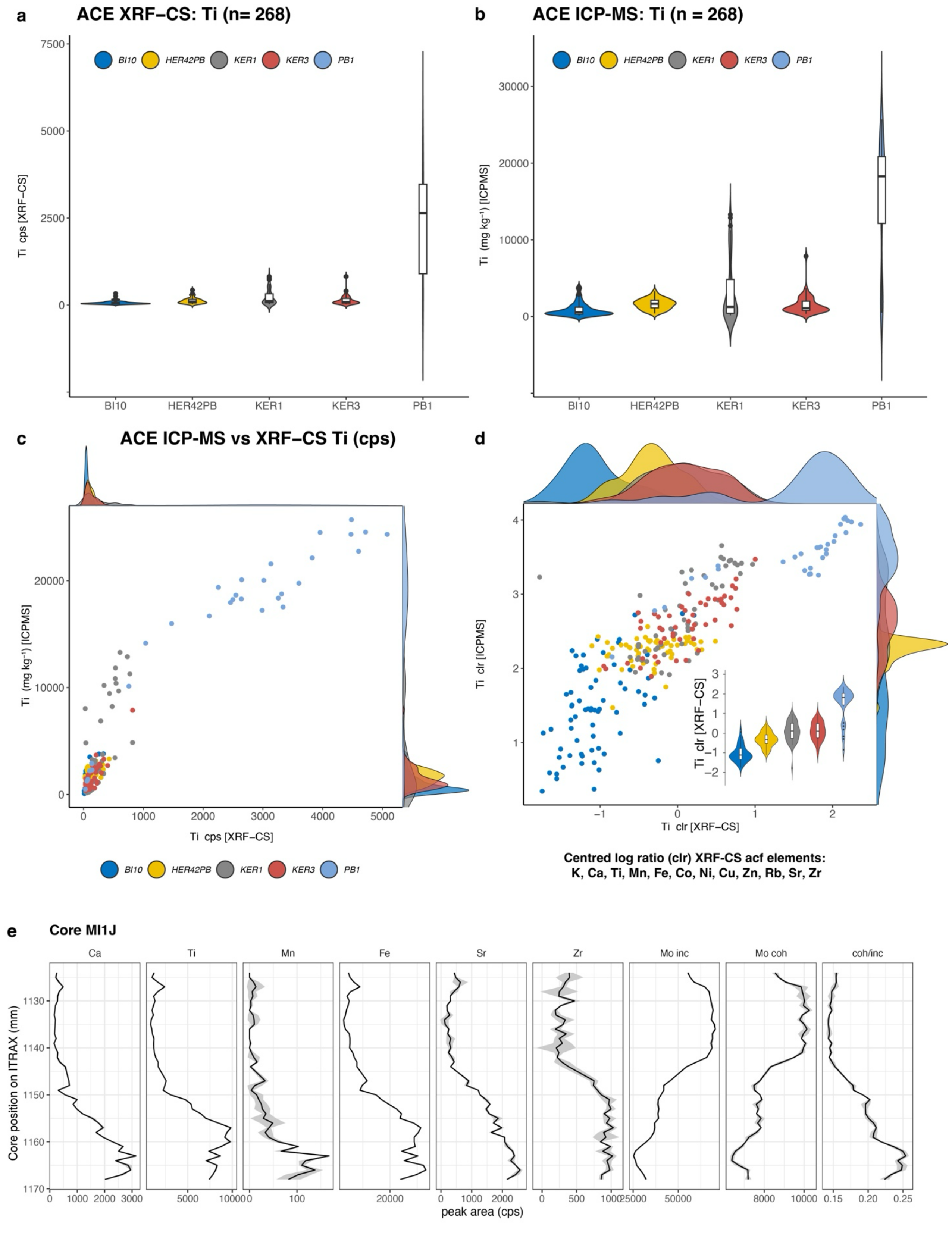

Supplementary Figure S3. (a) Correlation and density matrix comparing depth matched ICP-MS (in mg kg^{-1}) and XRF-CS data (in cps) for the six key elements from the five investigated peat core sites (n = 268). Correlations between the same ICP-MS and XRF-CS elements in red. * = 5% significance, ** = 1%; *** = 0.1%; (b) Individual site correlations for Ti cps data in Figure 2.

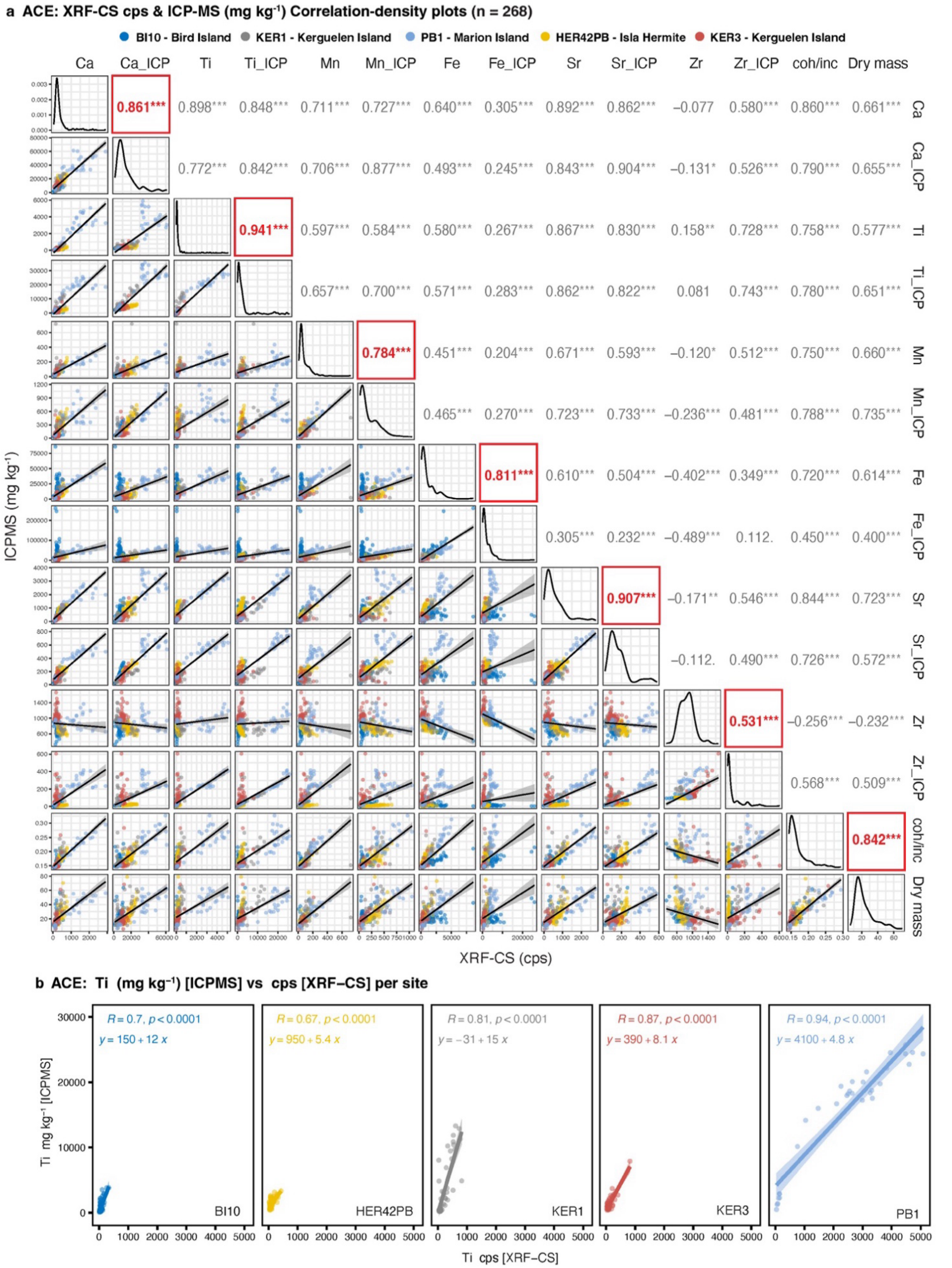

Supplementary Figure S4. Correlation and density matrix comparing centred-log ratios (clr) for the six key elements shown in Figure 2; clr calculations are from the 12 primary elements listed in the text. Correlations between the same ICP-MS and XRF-CS elements are highlighted in red. * = 5% significance, ** = 1% significance, *** = 0.1% significance; (b) Site correlations for Ti clr.

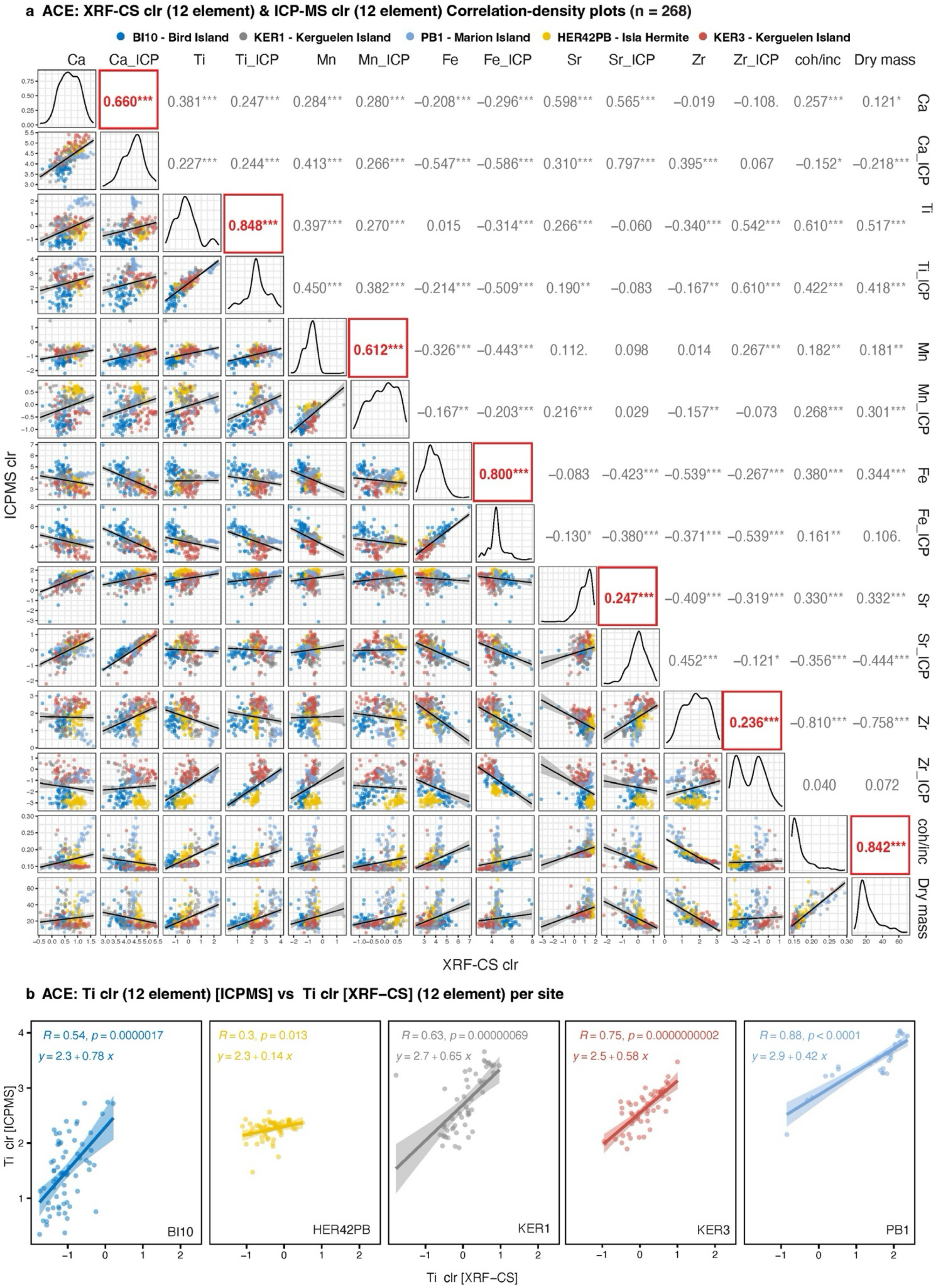

Supplementary Figure S5. (a) Correlation and density matrix of the depth-matched log-space ICP-MS and XRF-CS incoherent normalised data for the six key elements from the five peatland sites (n = 268). Correlations between the same ICP-MS and XRF-CS elements are highlighted in red. * = 5% significance , ** = 1%; *** = 0.1%; (b) Site correlations for log-space Ti data.

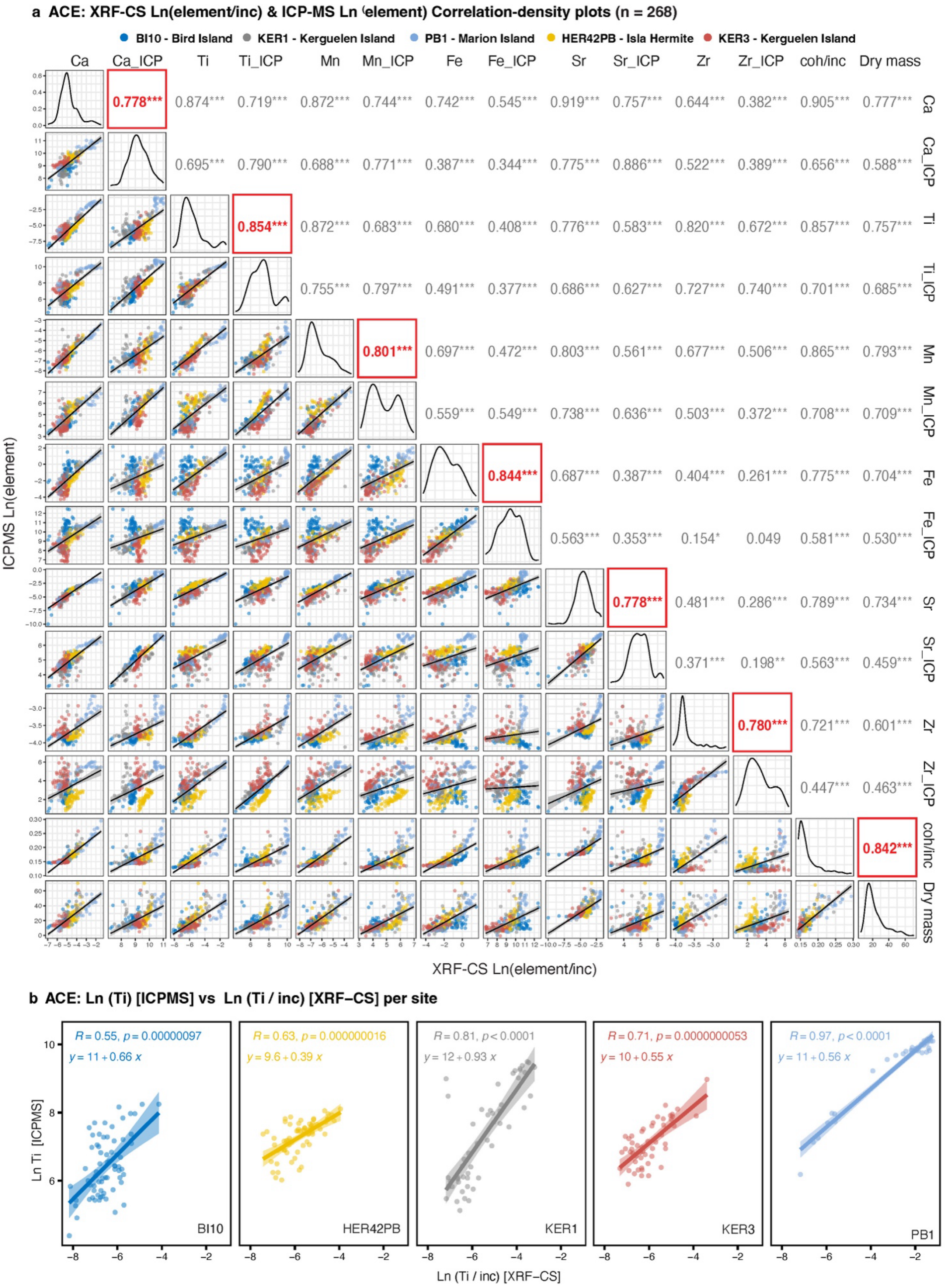

Supplementary Figure S6. Preliminary matched ICP-MS vs XRF-CS OLS and WLS univariate linear regression models undertaken on all 12 elements in the matched natural incoherent normalised XRF-CS and ICP-MS log-space dataset. **(a)** Six key minerogenic elements shown in Fig. 2 individually. **(b)** Six secondary elements (defined as acf threshold >0.1 but <0.5 after an XRF-CS interval lag of 20 datapoints (2 cm).

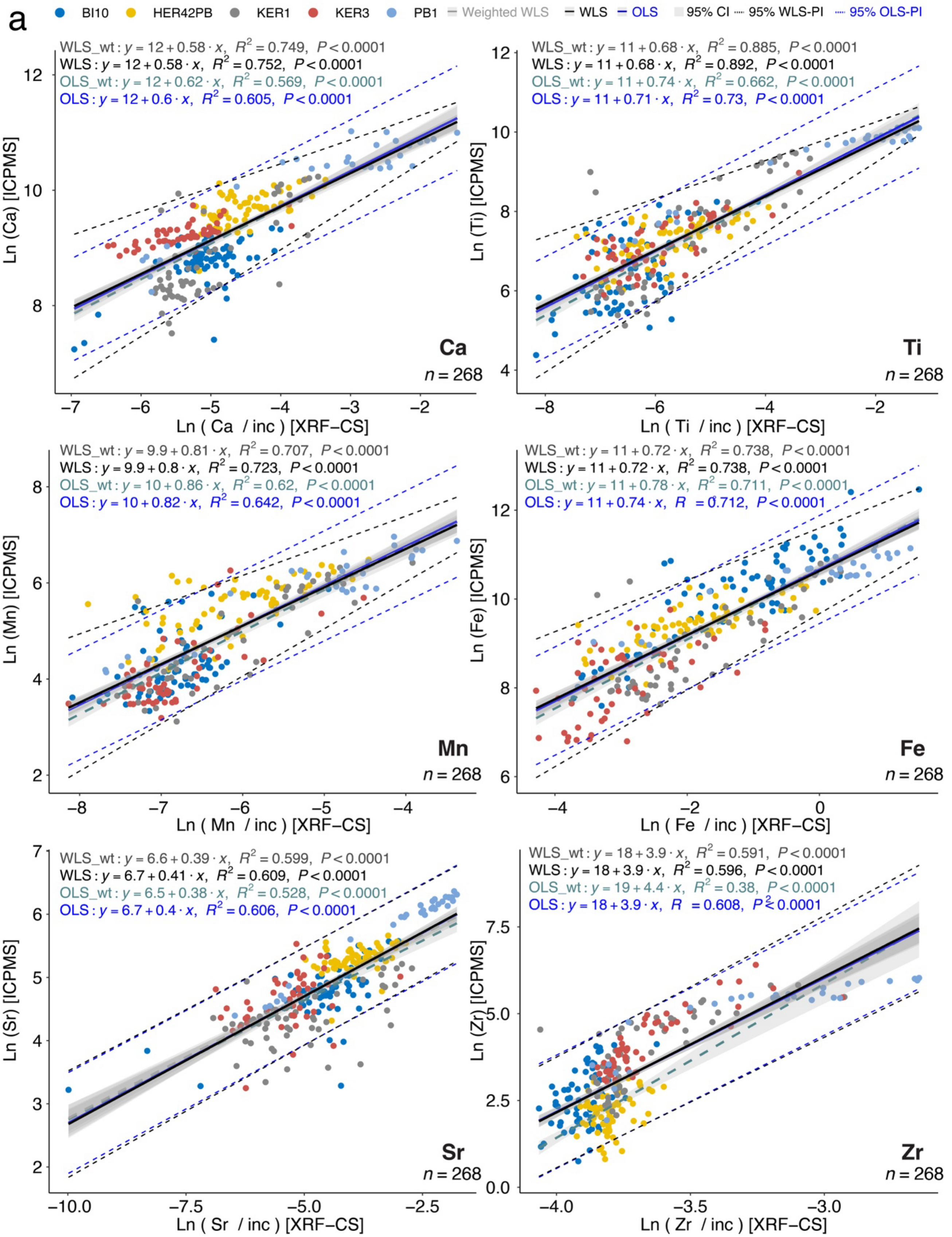

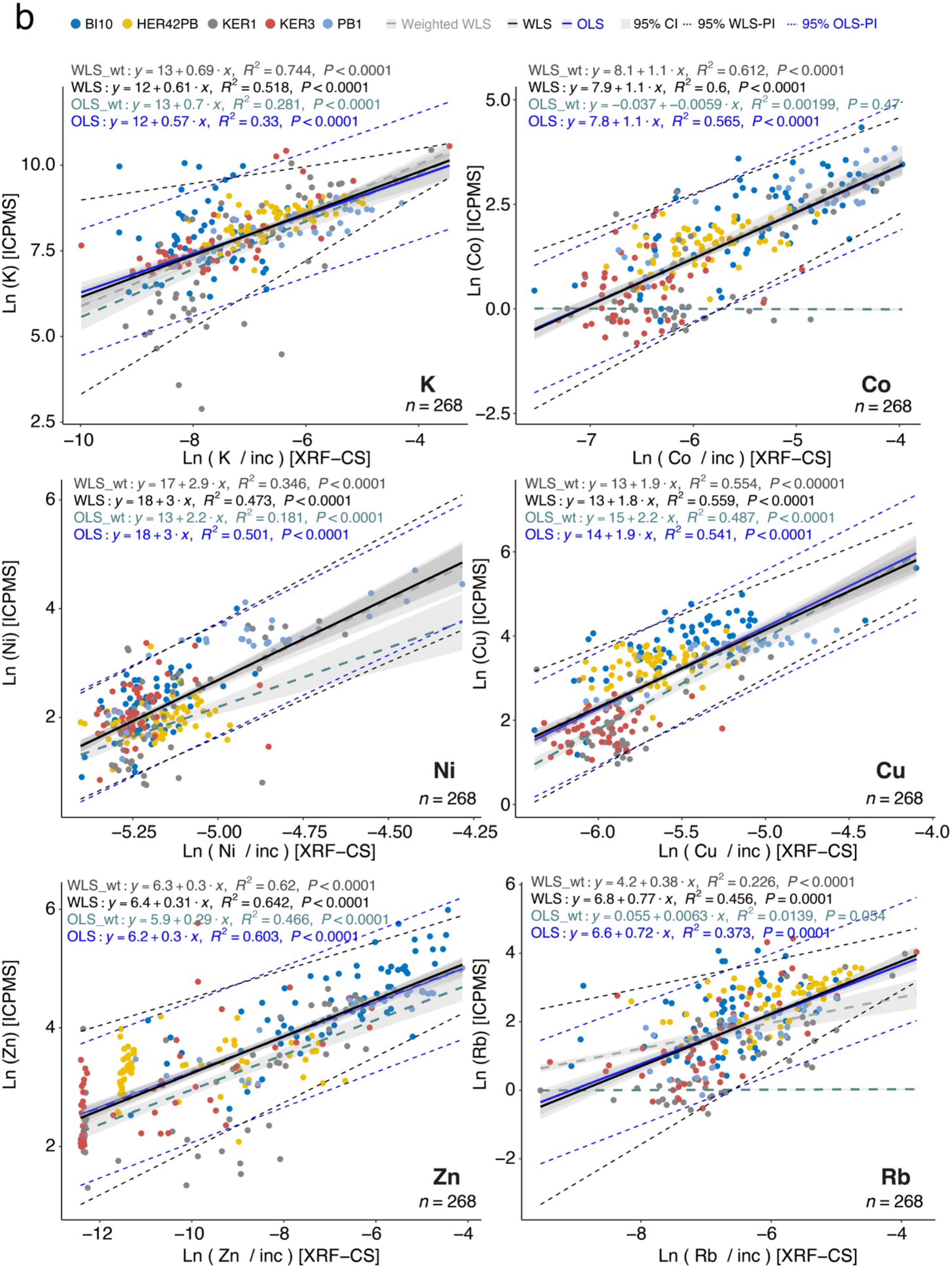
b
BI10 HER42PB KER1 KER3 PB1 Weighted WLS WLS OLS 95% CI 95% WLS-PI 95% OLS-PI
WLS_wt : y = 13 + 0.69 · x, R^2 = 0.744, P < 0.0001
WLS : y = 12 + 0.61 · x, R^2 = 0.518, P < 0.0001
OLS_wt : y = 13 + 0.7 · x, R^2 = 0.281, P < 0.0001
OLS : y = 12 + 0.57 · x, R^2 = 0.33, P < 0.0001
Ln (K) [ICPMS]
Ln (K / inc) [XRF−CS]
K
n = 268
WLS_wt : y = 8.1 + 1.1 · x, R^2 = 0.612, P < 0.0001
WLS : y = 7.9 + 1.1 · x, R^2 = 0.6, P < 0.0001
OLS_wt : y = −0.037 + −0.0059 · x, R^2 = 0.00199, P = 0.47
OLS : y = 7.8 + 1.1 · x, R^2 = 0.565, P < 0.0001
Ln (Co) [ICPMS]
Ln (Co / inc) [XRF−CS]
Co
n = 268
WLS_wt : y = 17 + 2.9 · x, R^2 = 0.346, P < 0.0001
WLS : y = 18 + 3 · x, R^2 = 0.473, P < 0.0001
OLS_wt : y = 13 + 2.2 · x, R^2 = 0.181, P < 0.0001
OLS : y = 18 + 3 · x, R^2 = 0.501, P < 0.0001
Ln (Ni) [ICPMS]
Ln (Ni / inc) [XRF−CS]
Ni
n = 268
WLS_wt : y = 13 + 1.9 · x, R^2 = 0.554, P < 0.00001
WLS : y = 13 + 1.8 · x, R^2 = 0.559, P < 0.0001
OLS_wt : y = 15 + 2.2 · x, R^2 = 0.487, P < 0.0001
OLS : y = 14 + 1.9 · x, R^2 = 0.541, P < 0.0001
Ln (Cu) [ICPMS]
Ln (Cu / inc) [XRF−CS]
Cu
n = 268
WLS_wt : y = 6.3 + 0.3 · x, R^2 = 0.62, P < 0.0001
WLS : y = 6.4 + 0.31 · x, R^2 = 0.642, P < 0.0001
OLS_wt : y = 5.9 + 0.29 · x, R^2 = 0.466, P < 0.0001
OLS : y = 6.2 + 0.3 · x, R^2 = 0.603, P < 0.0001
Ln (Zn) [ICPMS]
Ln (Zn / inc) [XRF−CS]
Zn
n = 268
WLS_wt : y = 4.2 + 0.38 · x, R^2 = 0.226, P < 0.0001
WLS : y = 6.8 + 0.77 · x, R^2 = 0.456, P = 0.0001
OLS_wt : y = 0.055 + 0.0063 · x, R^2 = 0.0139, P = 0.054
OLS : y = 6.6 + 0.72 · x, R^2 = 0.373, P = 0.0001
Ln (Rb) [ICPMS]
Ln (Rb / inc) [XRF−CS]
Rb
n = 268

Supplementary Figure S7. Standard performance[12] tests undertaken on each element in the univariate residual weighted least squares (WLS) regression plots shown in Figure S6, run for all elements but using Ti as an example here.

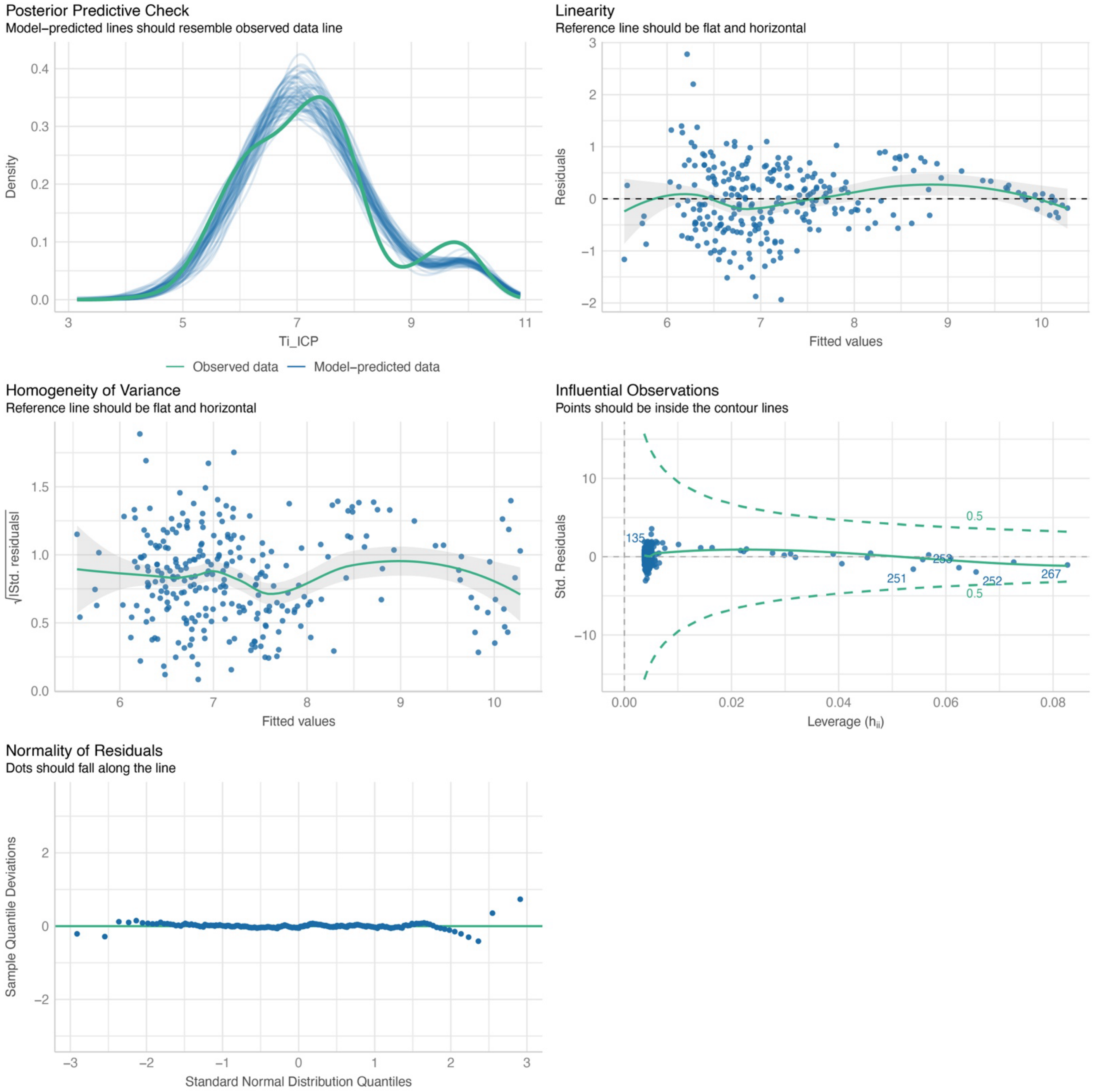

Supplementary Figure S8. Preliminary ICP-MS vs XRF-CS OLS and WLS linear regression models for six key elements (Ca, Ti, Fe, Mn, Sr, Zr) identified by autocorrelation from the XRF-CS dataset from the five investigated peat core sites. Plots shown centred-log ratio (clr) XRF-CS composite depth-matched XRF-CS cps and ICP-MS concentration datasets with clr calculations applied to 12 elements (K, Ca, Ti, Mn, Fe, Co, Ni, Cu, Zn, Rb, Sr, Zr) that were defined by applying an autocorrelation function to the XRF-CS dataset to determine elements with downcore variability that is different from noise. (p-values <0.0001 unless stated).

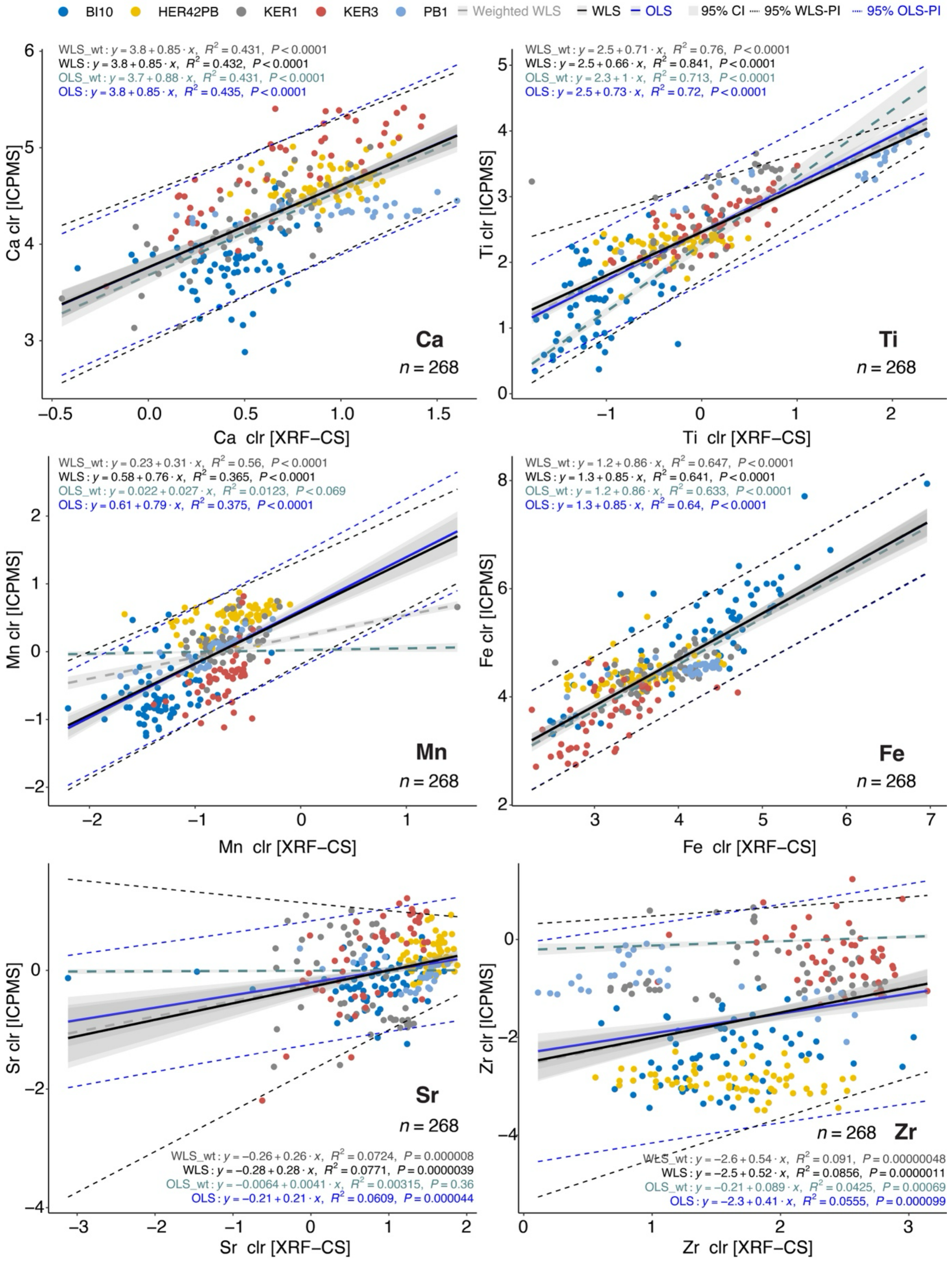

Supplementary Figure S9 (overpage). Summary predicted vs observed (measured) ICP-MS log-space plots for Ti using **(a)** Six-elements (Ca, Ti, Fe, Mn, Sr, Zr), and **(b)** four-elements (Ca, Ti, Sr, Zr) as components in four univariate (OLS, WLS, and weighted OLS, WLS) and four multivariate (Bayesian glm, Bayes, Random Forest, RF, PLS k-fold, and PLS-LOO) calibration models tested, with response variable y = ln ICP-MS and predictors x = ln (XRF-CS/inc.) and harmonised 10-fold cross validation. Elements were assessed individually for univariate models and simultaneously for multivariate models (n = 268) Calibration plots are shown in log space, with model performance R^2, RMSE (log- and concentration space in mg kg^{-1}), and 10-fold cross validation $RMSEP_{cv}$ and/or bootstrapped $RMSEP_b$ in concentration space in mg kg^{-1}. Grey bars are ±2×ICP-MS analytical uncertainty (horizontal) and predicted 95% CI (vertical); solid black line is the 1:1 line.

a
6-elements
(Ca, Ti, Fe, Mn, Sr , Zr)

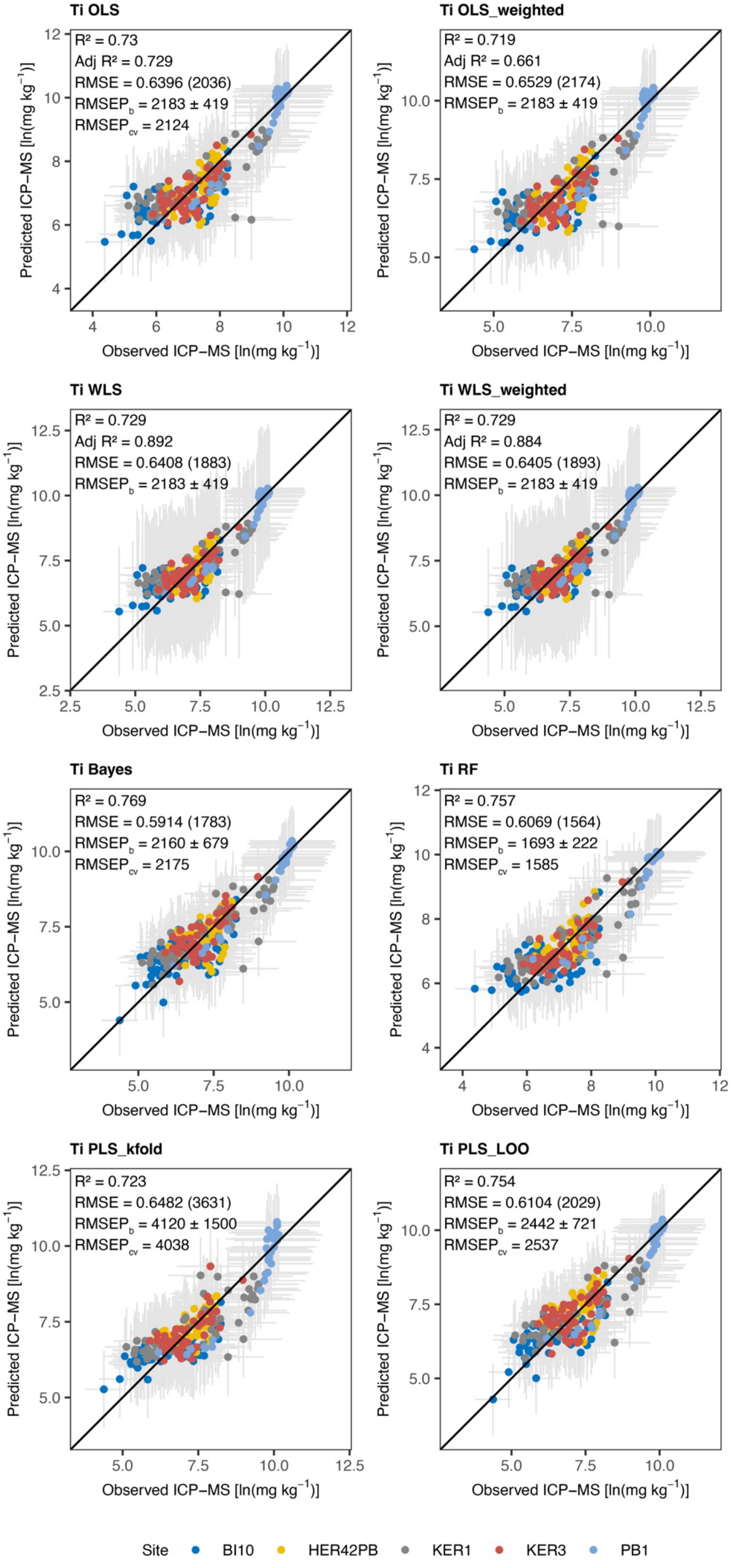

b

4-elements

(Ca, Ti, Sr , Zr)

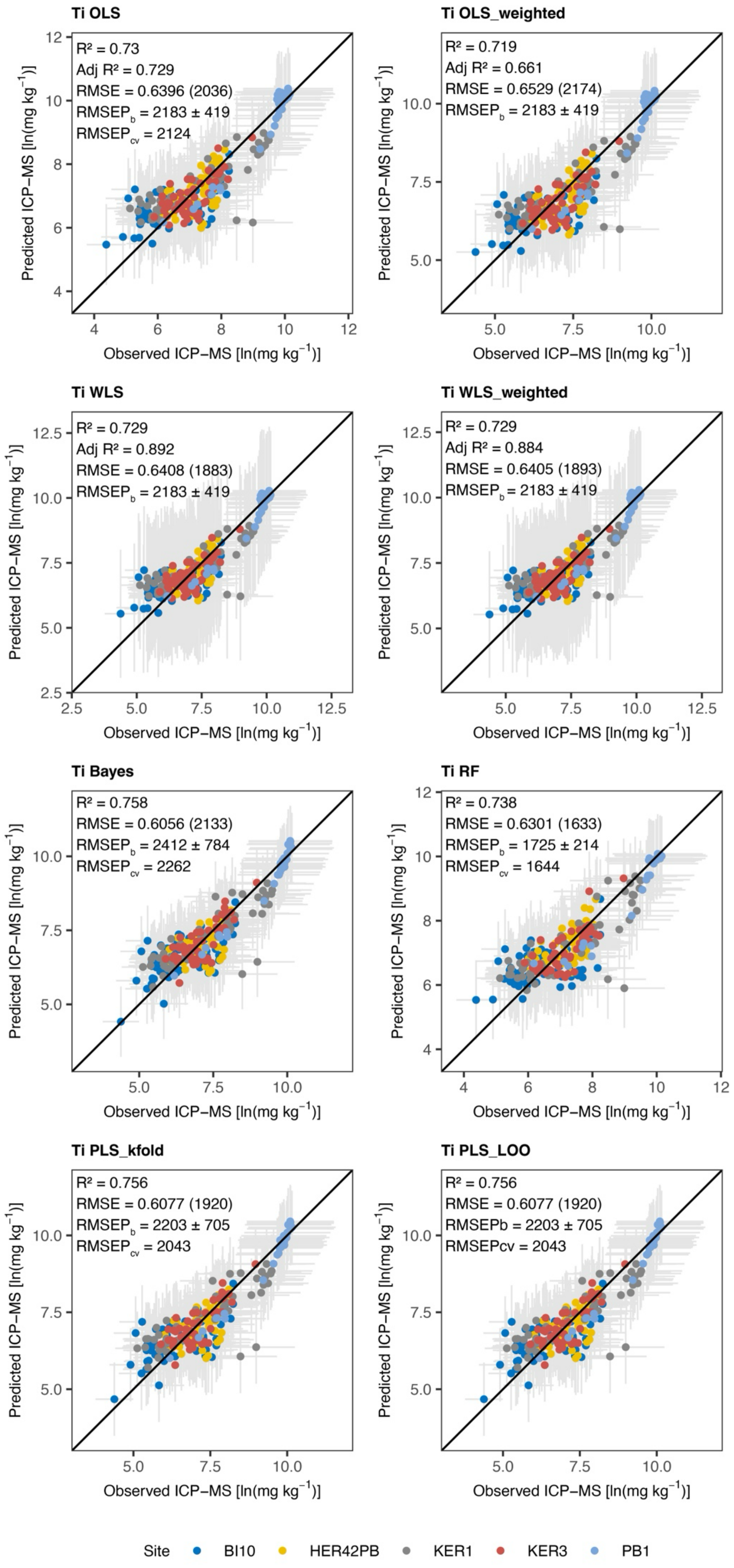

Supplementary Figure S10 (overpage) Summary predicted vs observed (measured) ICP-MS log-space plots for Zr using **(a)** Six-elements (Ca, Ti, Fe, Mn, Sr, Zr), and **(b)** Four-elements (Ca, Ti, Sr, Zr) as components in four univariate (OLS, WLS, and weighted OLS, WLS) and four multivariate (Bayesian glm, Bayes, Random Forest, RF, PLS k-fold, and PLS-LOO) calibration models tested, with response variable y = ln ICP-MS and predictors x = ln (XRF-CS/inc.) and harmonised 10-fold cross validation. Elements were assessed individually for univariate models and simultaneously for multivariate models (n = 268). Calibration plots are shown in log space, with model performance R^2, RMSE (log- and concentration space in mg kg^{-1}), and 10-fold cross validation $RMSEP_{cv}$ and/or bootstrapped $RMSEP_b$ in concentration space in mg kg^{-1}. Grey bars are ±2×ICP-MS analytical uncertainty (horizontal) and predicted 95% CI (vertical); solid black line is the 1:1 line.

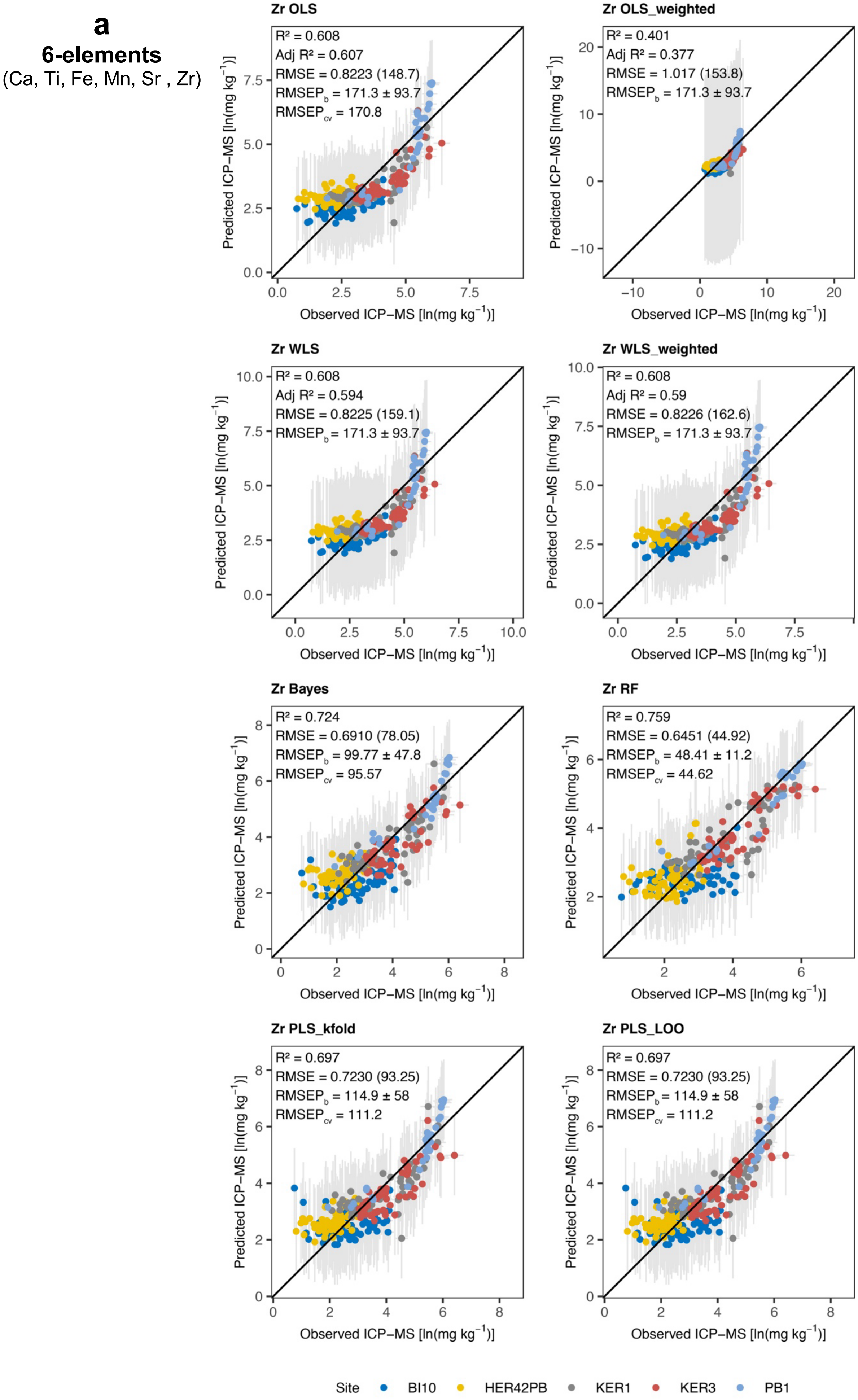
a
6-elements
(Ca, Ti, Fe, Mn, Sr , Zr)
Zr OLS
R² = 0.608
Adj R² = 0.607
RMSE = 0.8223 (148.7)
RMSEP$_b$ = 171.3 ± 93.7
RMSEP$_{cv}$ = 170.8
Zr OLS_weighted
R² = 0.401
Adj R² = 0.377
RMSE = 1.017 (153.8)
RMSEP$_b$ = 171.3 ± 93.7
Zr WLS
R² = 0.608
Adj R² = 0.594
RMSE = 0.8225 (159.1)
RMSEP$_b$ = 171.3 ± 93.7
Zr WLS_weighted
R² = 0.608
Adj R² = 0.59
RMSE = 0.8226 (162.6)
RMSEP$_b$ = 171.3 ± 93.7
Zr Bayes
R² = 0.724
RMSE = 0.6910 (78.05)
RMSEP$_b$ = 99.77 ± 47.8
RMSEP$_{cv}$ = 95.57
Zr RF
R² = 0.759
RMSE = 0.6451 (44.92)
RMSEP$_b$ = 48.41 ± 11.2
RMSEP$_{cv}$ = 44.62
Zr PLS_kfold
R² = 0.697
RMSE = 0.7230 (93.25)
RMSEP$_b$ = 114.9 ± 58
RMSEP$_{cv}$ = 111.2
Zr PLS_LOO
R² = 0.697
RMSE = 0.7230 (93.25)
RMSEP$_b$ = 114.9 ± 58
RMSEP$_{cv}$ = 111.2
Predicted ICP−MS [ln(mg kg^{-1})]
Observed ICP−MS [ln(mg kg^{-1})]
Site BI10 HER42PB KER1 KER3 PB1

10b
4-elements
(Ca, Ti, Sr , Zr)

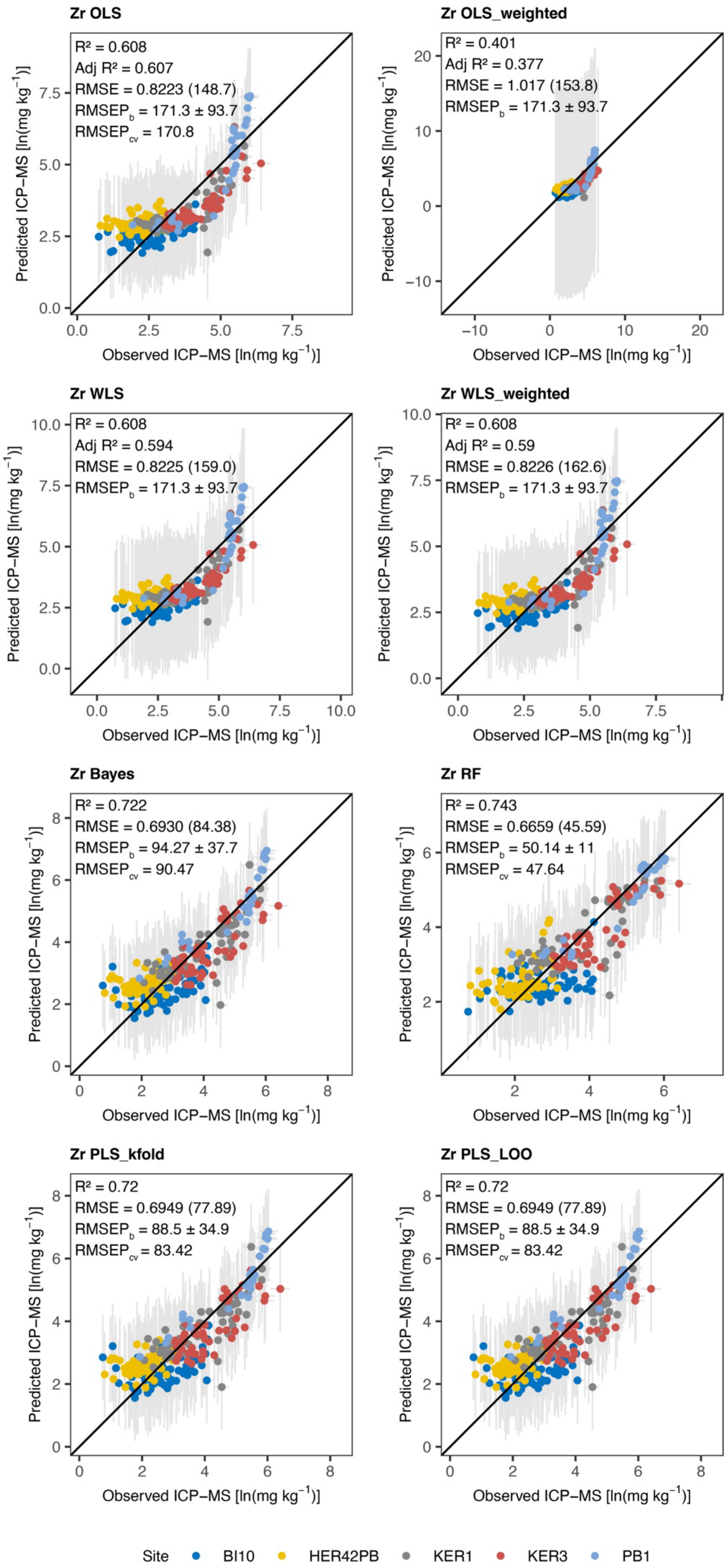

Supplementary Figure S11 (overpage). Principal component analysis (PCA) and Partial Least Squares (PLS) plots summarising components and overall correlations for the centred log ratio (clr) matched dataset for all sites. **(a)** PCA of all composite depth-matched XRF-CS and ICP-MS dataset elements defined by autocorrelation (acf) analysis, scatter parameters, dry mass and percentage organic carbon; **(b)** PCA of key elements from (a), which represent, on average, 95.8 ± 1.6 % of the original XRF-CS cps composite depth-matched XRF-CS and ICP-MS dataset, dry mass and carbon content (%C). A clear distinction can be made between non-volcanic islands (Bird Island and Isla Hermite) on the left (broadly negative PC values) and volcanic islands (positive PC values) is apparent, and likely due to the greater organic content of the non-volcanic peat cores and/or the more minerogenic nature of peat records from volcanic sub-Antarctic islands; **(c)** PCA of key elements from (a) only, highlighting a similar division between volcanic and non-volcanic islands; **(d)** PLS component 1 and 2 bi-plot for all sites for the four-component log inc. model with elements Ti, Ca, Sr, Zr from the matched log element/inc. XRF-CS and log ICP-MS dataset, divided in proportion 0.6:0.4 training: test dataset for bootstrapping (n = 1000); **(e)** Histogram of predicted Ti RMSE values for the log inc. model in (d). Overall, Ti ICP-MS and Ti XRF-CS are well correlated, particularly so at KER1 and PB1. Zr is not always well correlated but reflects the composition of the minerogenic component of KER1 and KER3 more than other sites. Dry mass and coh./inc. scatter are generally well correlated but with components split between PC axes 1 and 2, highlighting that, overall, the peat matrix composition from all sites is a mixture of organic and inorganic deposition. Data was analysed using the *pls*.R and *compositions*.R packages[1,11].

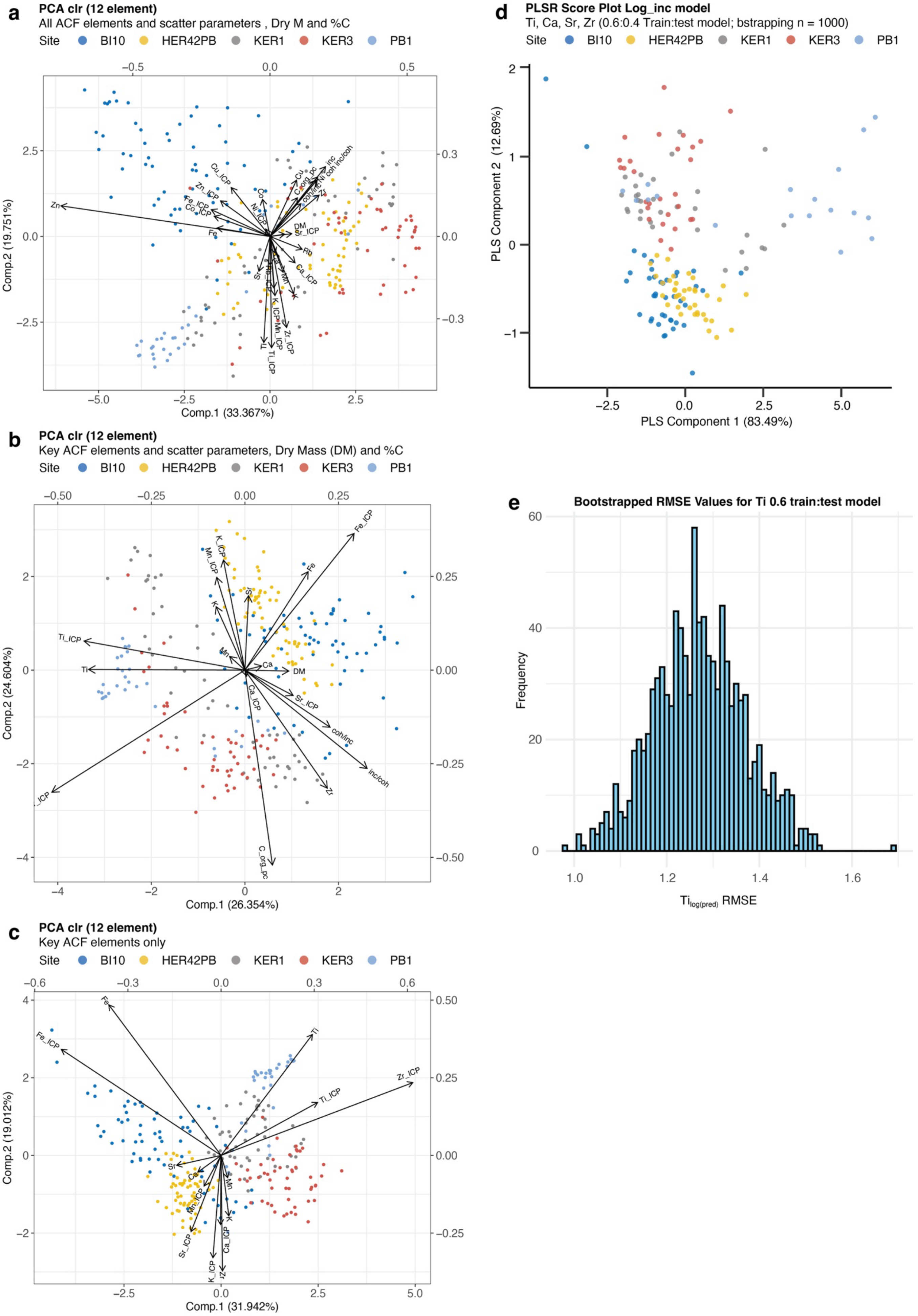
a
PCA clr (12 element)
All ACF elements and scatter parameters , Dry M and %C
Site BI10 HER42PB KER1 KER3 PB1
Comp.1 (33.367%)
Comp.2 (19.751%)
b
PCA clr (12 element)
Key ACF elements and scatter parameters, Dry Mass (DM) and %C
Site BI10 HER42PB KER1 KER3 PB1
Comp.1 (26.354%)
Comp.2 (24.604%)
c
PCA clr (12 element)
Key ACF elements only
Site BI10 HER42PB KER1 KER3 PB1
Comp.1 (31.942%)
Comp.2 (19.012%)
d
PLSR Score Plot Log_inc model
Ti, Ca, Sr, Zr (0.6:0.4 Train:test model; bstrapping n = 1000)
Site BI10 HER42PB KER1 KER3 PB1
PLS Component 1 (83.49%)
PLS Component 2 (12.69%)
e
Bootstrapped RMSE Values for Ti 0.6 train:test model
Frequency
$Ti_{log(pred)}$ RMSE

Supplementary Figure S12. Comparison of sub-sample dry mass and depth-matched XRF-CS coherent/incoherent (coh./inc.$_{Mo}$)[13] ratio datasets for: **(a)** all peatland sites in the ACE Subsample – XRF-CS composite depth-matched dataset (n = 1111); **(b)** the ACE Subsample – ICP-MS – XRF-CS composite depth-matched dataset (n = 268); **(c)** BI10, Bird Island (n = 419); **(d)** HER42PB, Isla Hermite (n = 299); **(e)** KER1, Kerguelen Island (n = 170); **(f)** KER3, Kerguelen Island (n = 153); **(g)** PB1, Marion Island (n = 74); **(h)** Comparison of organic carbon content measured in subsamples (derived from loss-on-ignition data and the SOM/SOC ratio of peat composed primarily of vascular plants of 1.73 ± 0.09[14]) and depth-matched XRF-CS incoherent/coherent (coh./inc.$_{Mo}$)[15] ratios for the ACE matched Subsample–XRF-CS dataset (n = 1115).

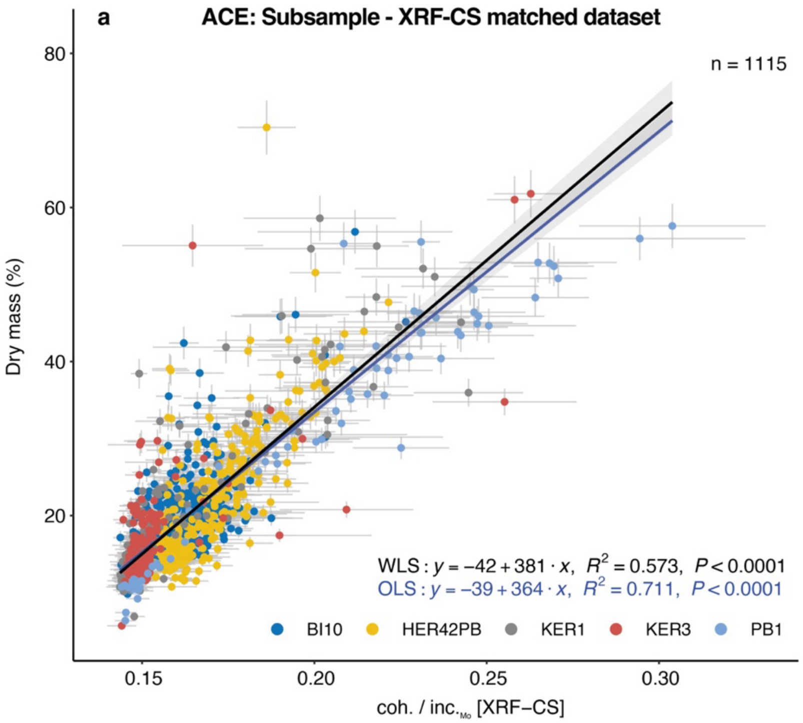

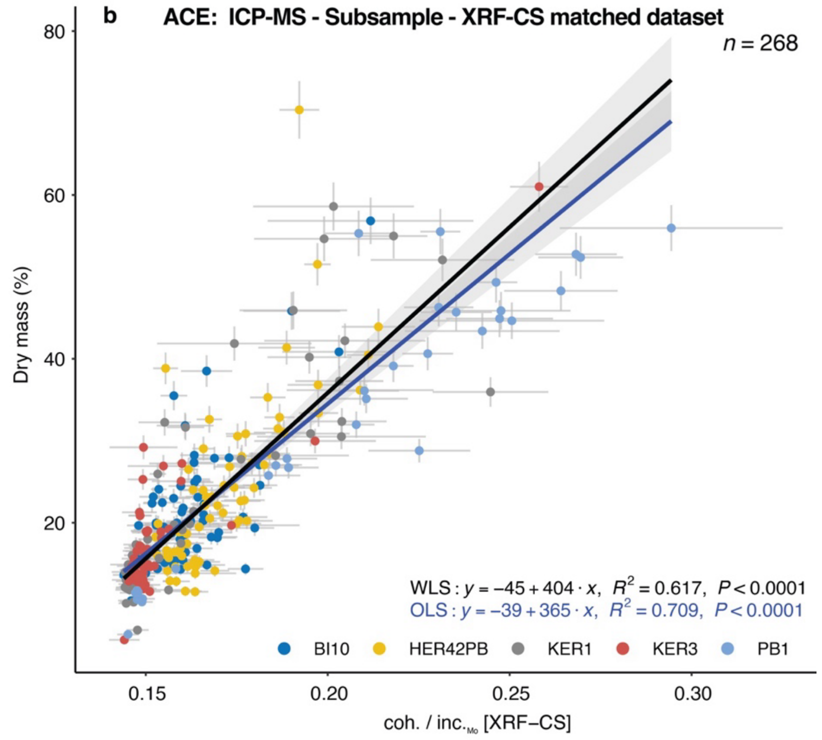

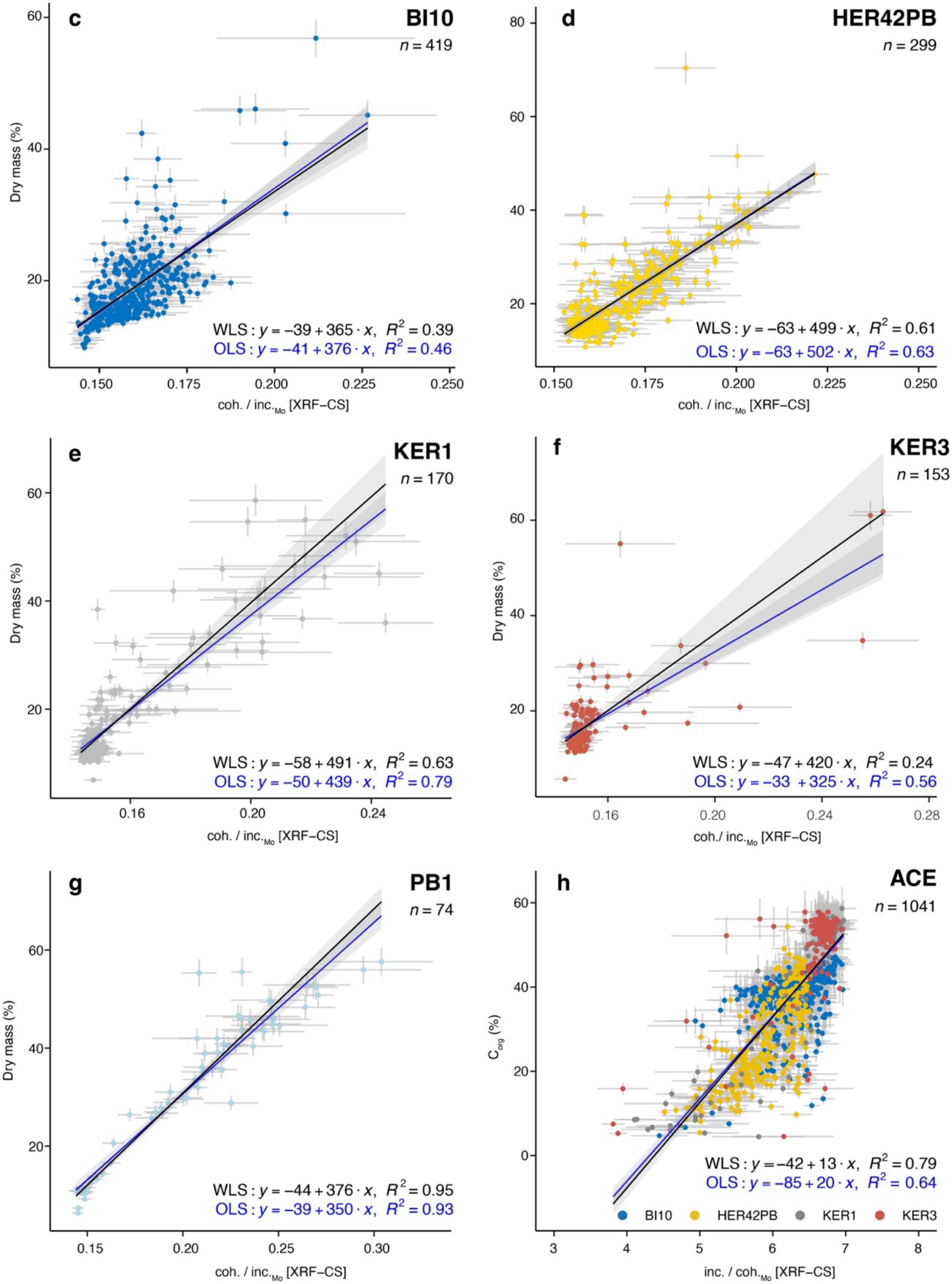

c
BI10
n = 419
Dry mass (%)
WLS : y = −39 + 365 · x, R² = 0.39
OLS : y = −41 + 376 · x, R² = 0.46
coh. / inc.Mo [XRF−CS]
d
HER42PB
n = 299
WLS : y = −63 + 499 · x, R² = 0.61
OLS : y = −63 + 502 · x, R² = 0.63
coh. / inc.Mo [XRF−CS]
e
KER1
n = 170
Dry mass (%)
WLS : y = −58 + 491 · x, R² = 0.63
OLS : y = −50 + 439 · x, R² = 0.79
coh. / inc.Mo [XRF−CS]
f
KER3
n = 153
Dry mass (%)
WLS : y = −47 + 420 · x, R² = 0.24
OLS : y = −33 + 325 · x, R² = 0.56
coh./ inc.Mo [XRF−CS]
g
PB1
n = 74
Dry mass (%)
WLS : y = −44 + 376 · x, R² = 0.95
OLS : y = −39 + 350 · x, R² = 0.93
coh. / inc.Mo [XRF−CS]
h
ACE
n = 1041
Corg (%)
WLS : y = −42 + 13 · x, R² = 0.79
OLS : y = −85 + 20 · x, R² = 0.64
BI10
HER42PB
KER1
KER3
inc. / coh.Mo [XRF−CS]

Supplementary Figure S13 Peatland record BI10 predicted downcore predicted concentration plots generated by **(a)** 6-element (Ca, Ti, Fe, Mn, Sr, Zr) and for **(b)** 4-element (Ca, Ti, Sr, Zr) univariate (OLS, WLS) and multivariate (Bayes glm, RF, PLS-LOO, PLS-kfold) calibration model runs compared with measured ICP-MS data (blue points/lines).

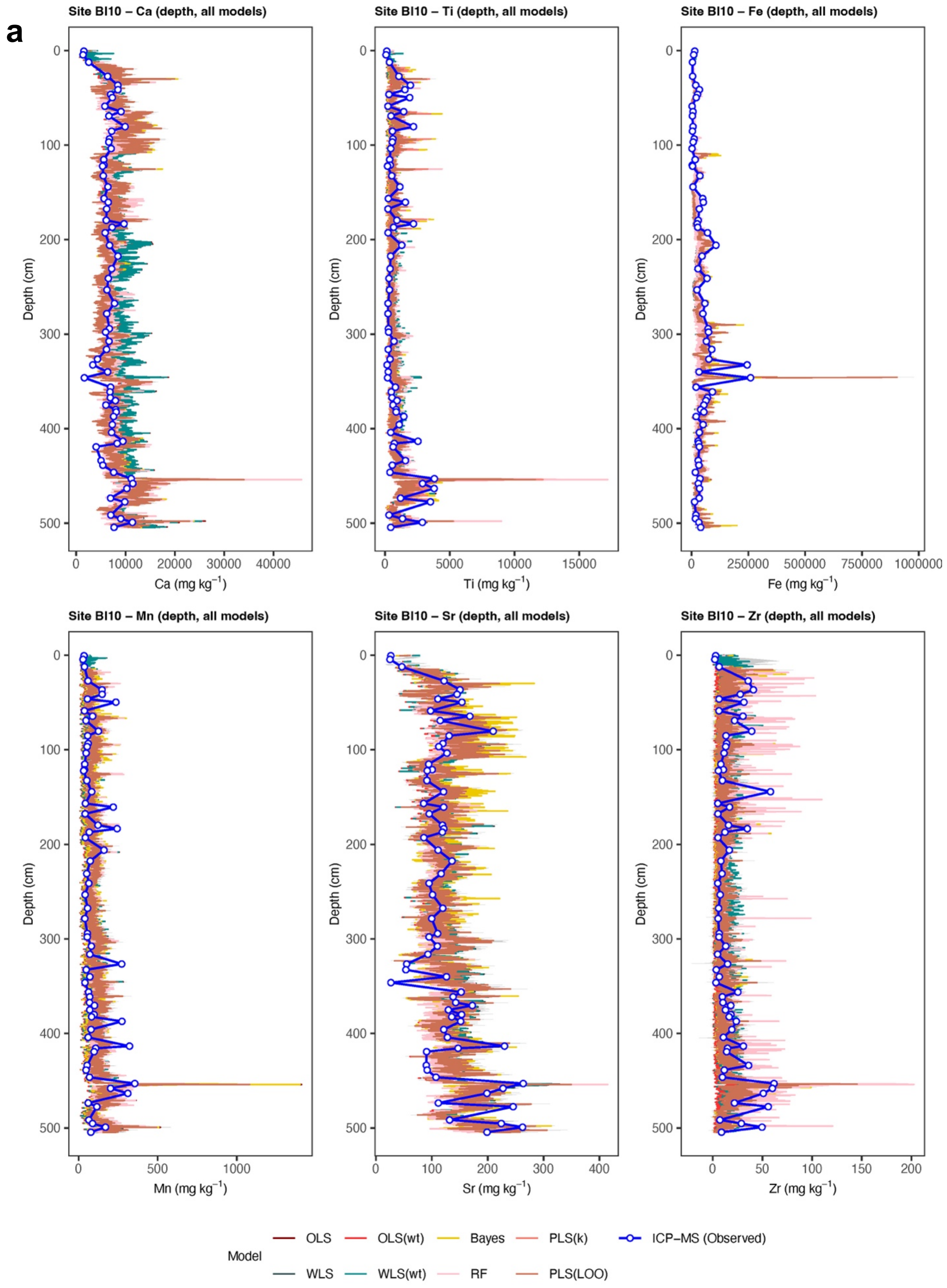

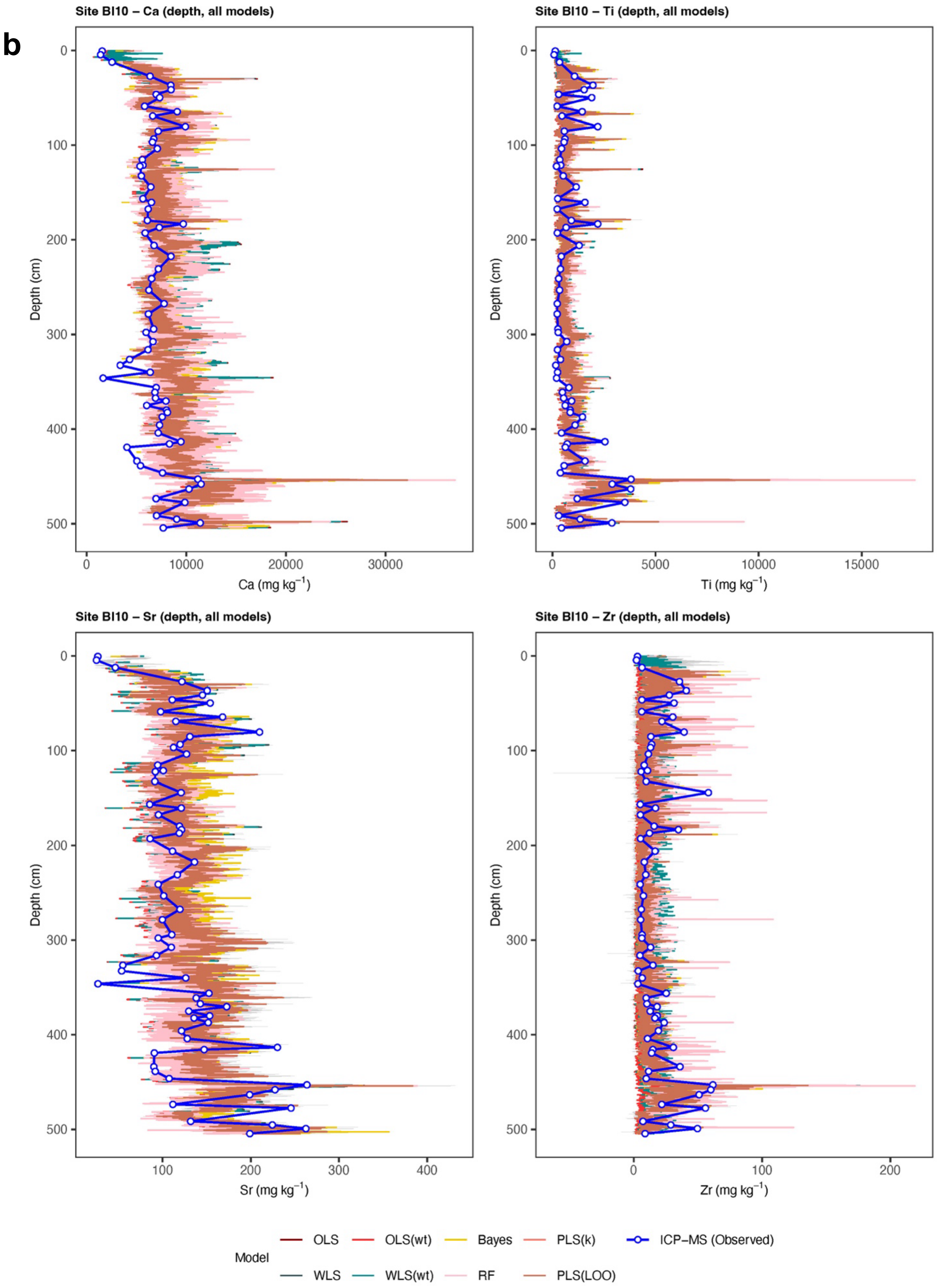

b
Site BI10 – Ca (depth, all models)
Site BI10 – Ti (depth, all models)
Site BI10 – Sr (depth, all models)
Site BI10 – Zr (depth, all models)
Depth (cm)
Ca (mg kg^{-1})
Ti (mg kg^{-1})
Sr (mg kg^{-1})
Zr (mg kg^{-1})
Model
OLS
OLS(wt)
Bayes
PLS(k)
ICP−MS (Observed)
WLS
WLS(wt)
RF
PLS(LOO)

Supplementary Figure S14 Peatland record HER42PB predicted downcore predicted concentration plots generated by **(a)** 6-element (Ca, Ti, Fe, Mn, Sr, Zr) and for **(b)** 4-element (Ca, Ti, Sr, Zr) univariate (OLS, WLS) and multivariate (Bayes glm, RF, PLS-LOO, PLS-kfold) calibration model runs compared with measured ICP-MS data (blue points/lines).

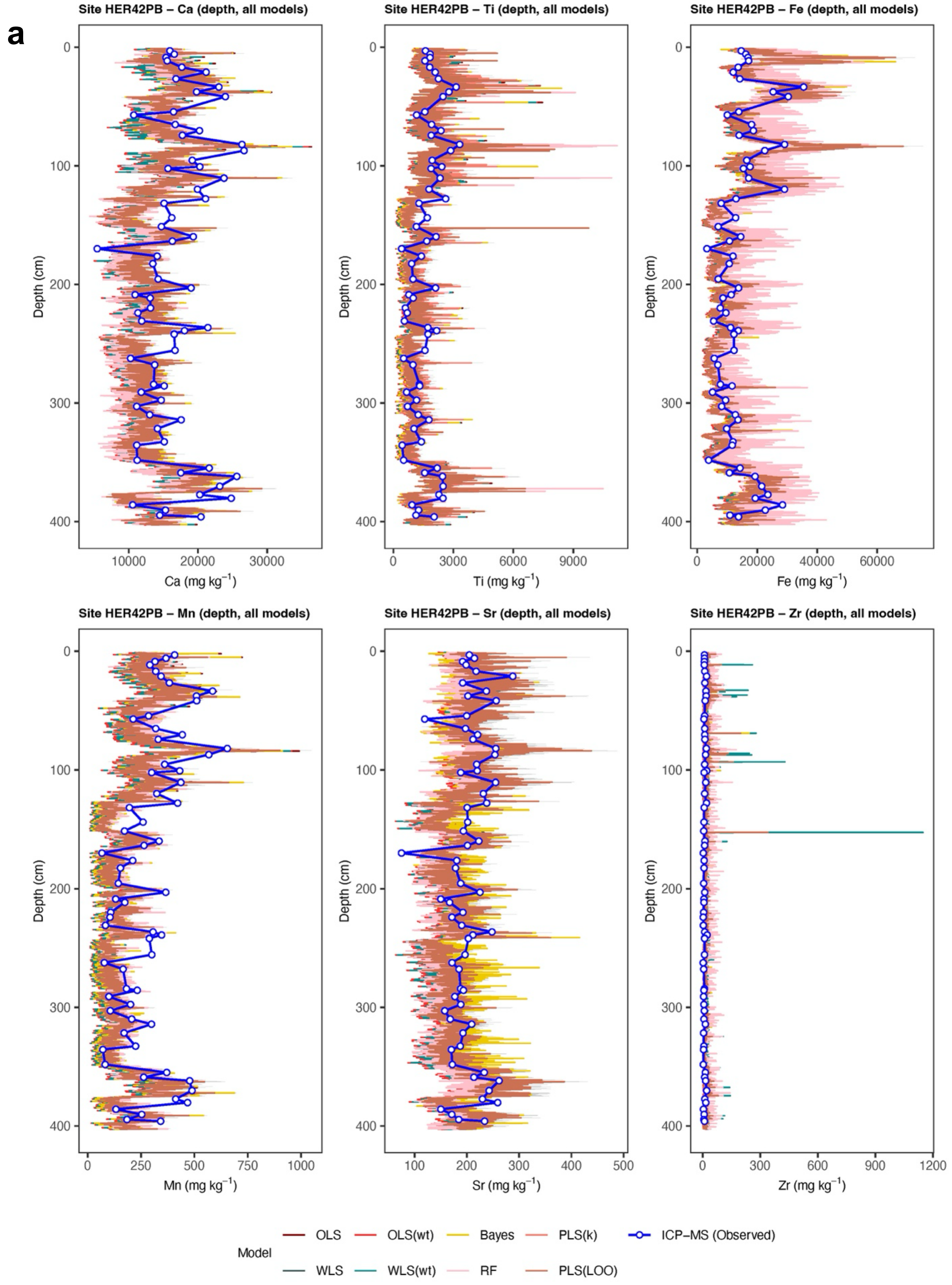

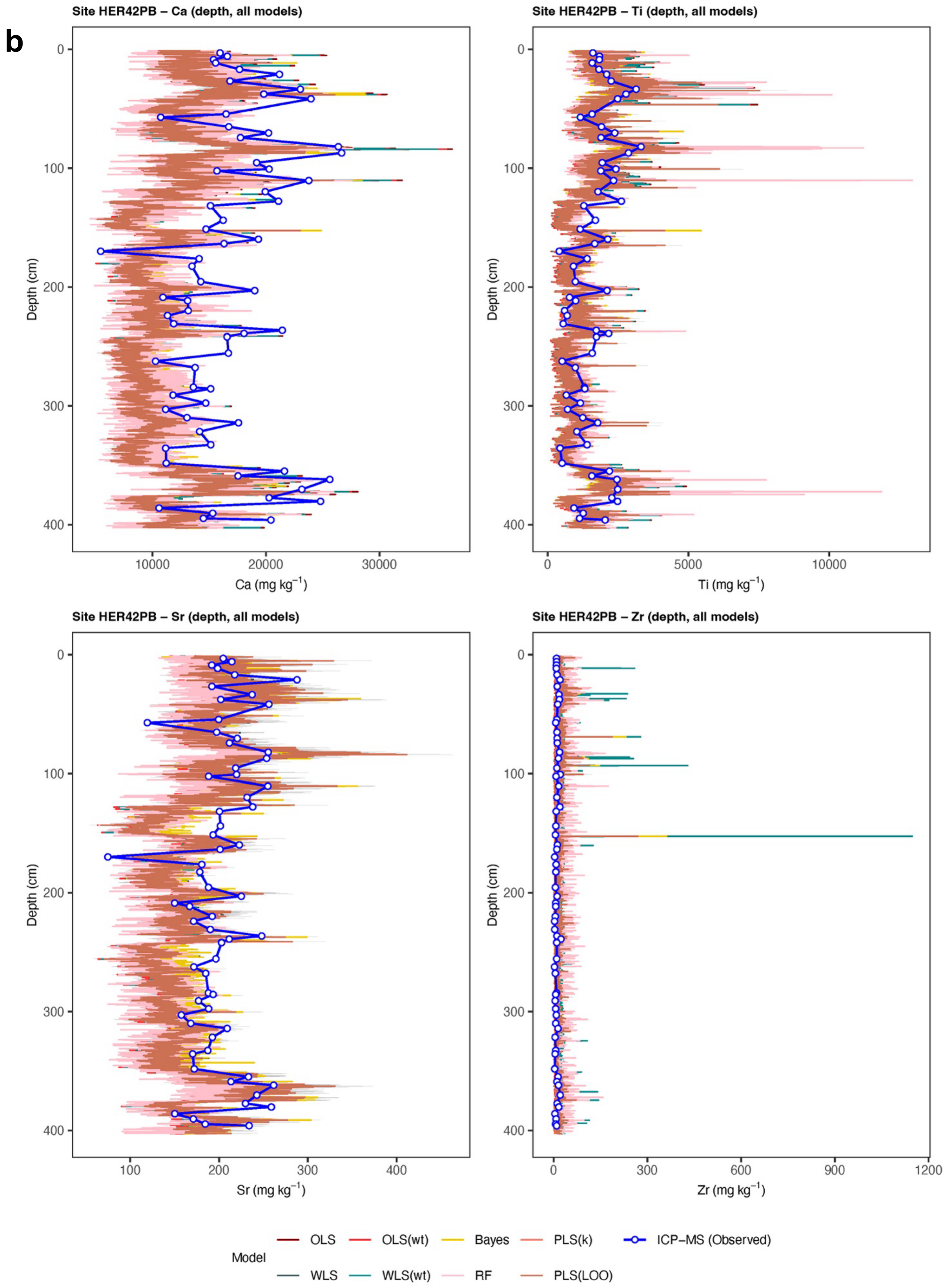
b
Site HER42PB – Ca (depth, all models)
Site HER42PB – Ti (depth, all models)
Site HER42PB – Sr (depth, all models)
Site HER42PB – Zr (depth, all models)
Depth (cm)
Ca (mg kg−1)
Ti (mg kg−1)
Sr (mg kg−1)
Zr (mg kg−1)
Model
OLS
OLS(wt)
Bayes
PLS(k)
ICP−MS (Observed)
WLS
WLS(wt)
RF
PLS(LOO)

Supplementary Figure S15 Peatland record KER1 predicted downcore predicted concentration plots generated by **(a)** 6-element (Ca, Ti, Fe, Mn, Sr, Zr) and for **(b)** 4-element (Ca, Ti, Sr, Zr) univariate (OLS, WLS) and multivariate (Bayes glm, RF, PLS-LOO, PLS-kfold) calibration model runs compared with measured ICP-MS data (blue points/lines).

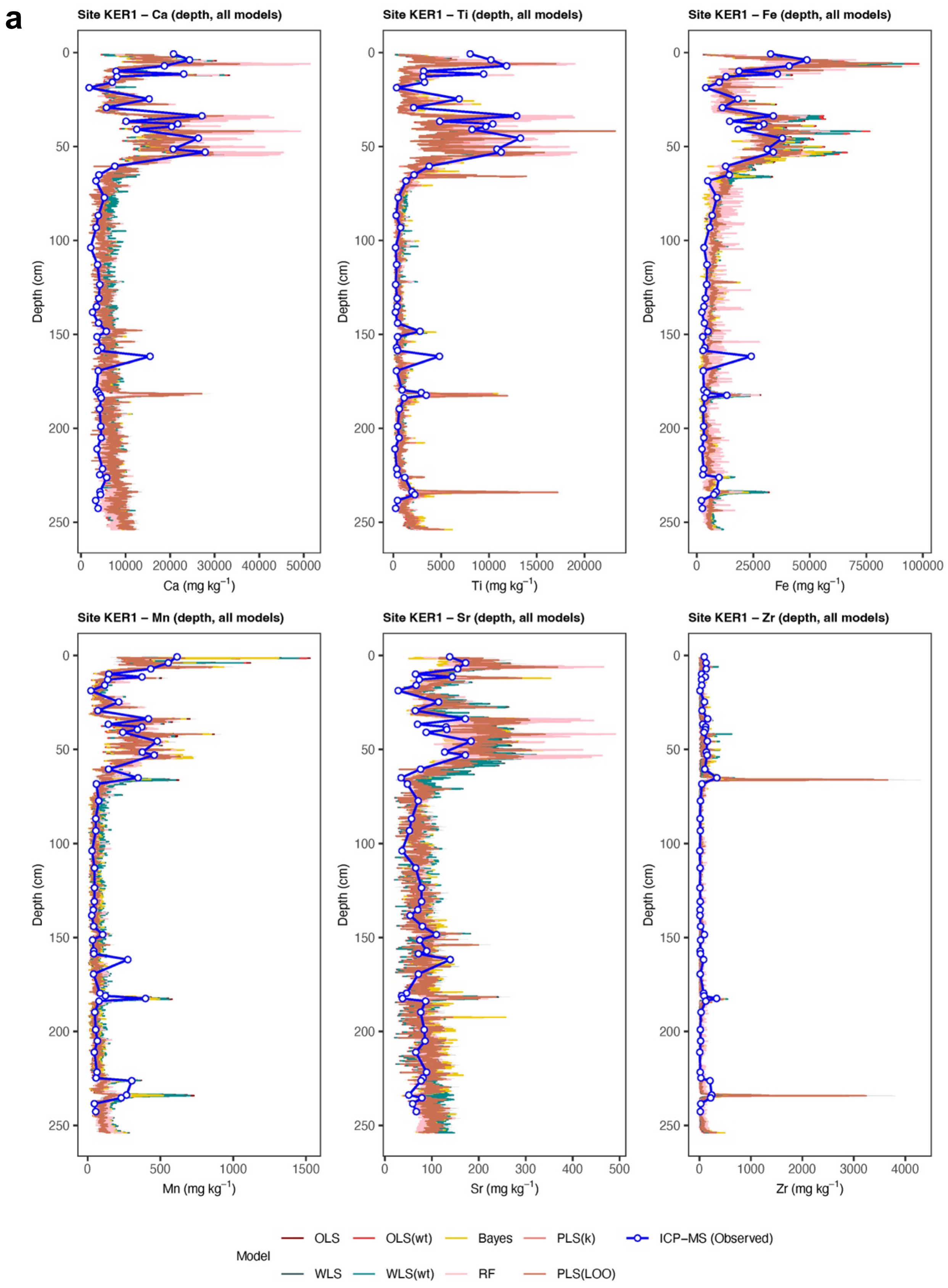

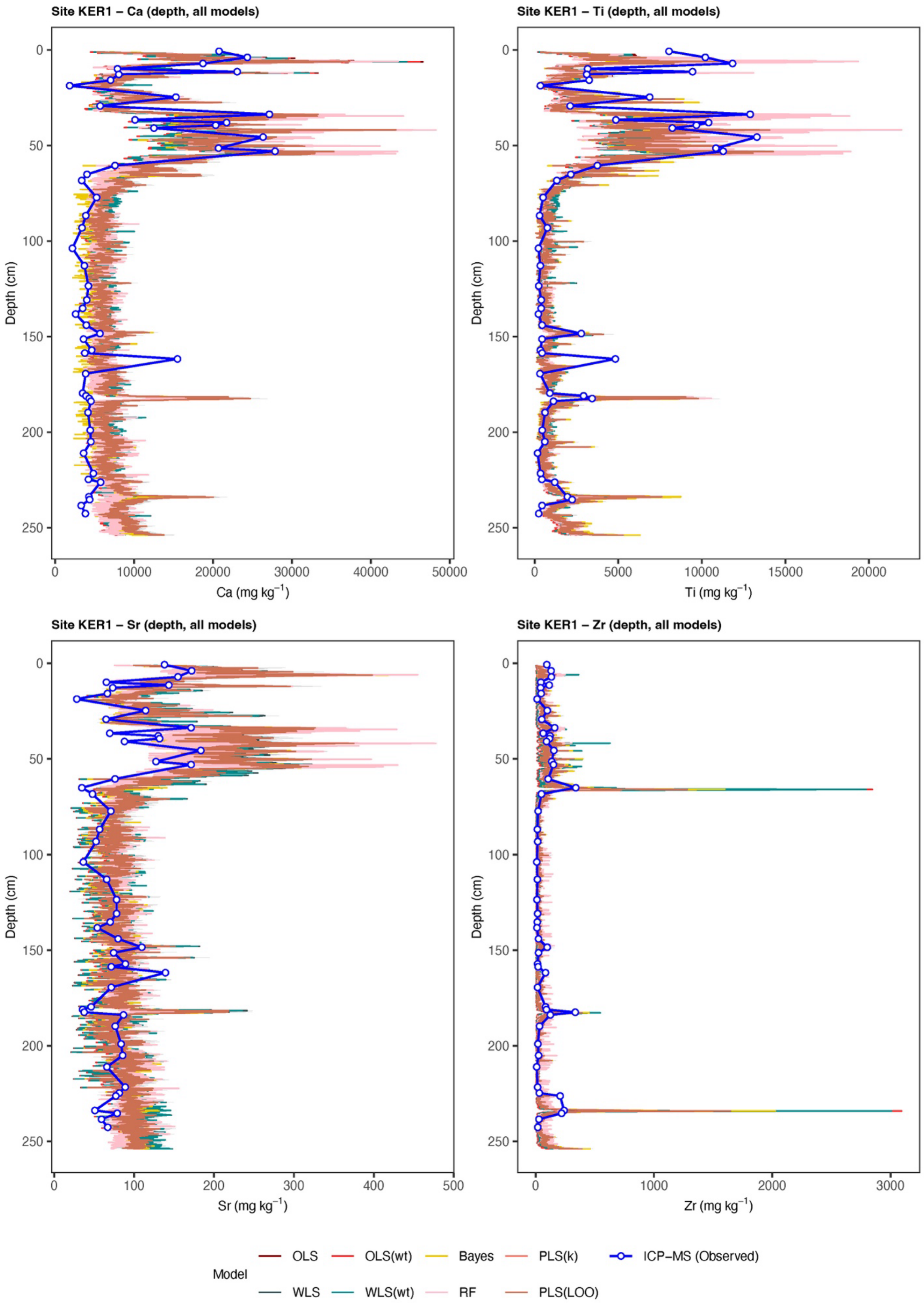

Site KER1 – Ca (depth, all models)
Site KER1 – Ti (depth, all models)
Site KER1 – Sr (depth, all models)
Site KER1 – Zr (depth, all models)
Depth (cm)
Ca (mg kg−1)
Ti (mg kg−1)
Sr (mg kg−1)
Zr (mg kg−1)
Model
OLS
OLS(wt)
Bayes
PLS(k)
ICP−MS (Observed)
WLS
WLS(wt)
RF
PLS(LOO)

Supplementary Figure S16 Peatland record KER3 predicted downcore predicted concentration plots generated by **(a)** 6-element (Ca, Ti, Fe, Mn, Sr, Zr) and for **(b)** 4-element (Ca, Ti, Sr, Zr) univariate (OLS, WLS) and multivariate (Bayes glm, RF, PLS-LOO, PLS-kfold) calibration model runs compared with measured ICP-MS data (blue points/lines).

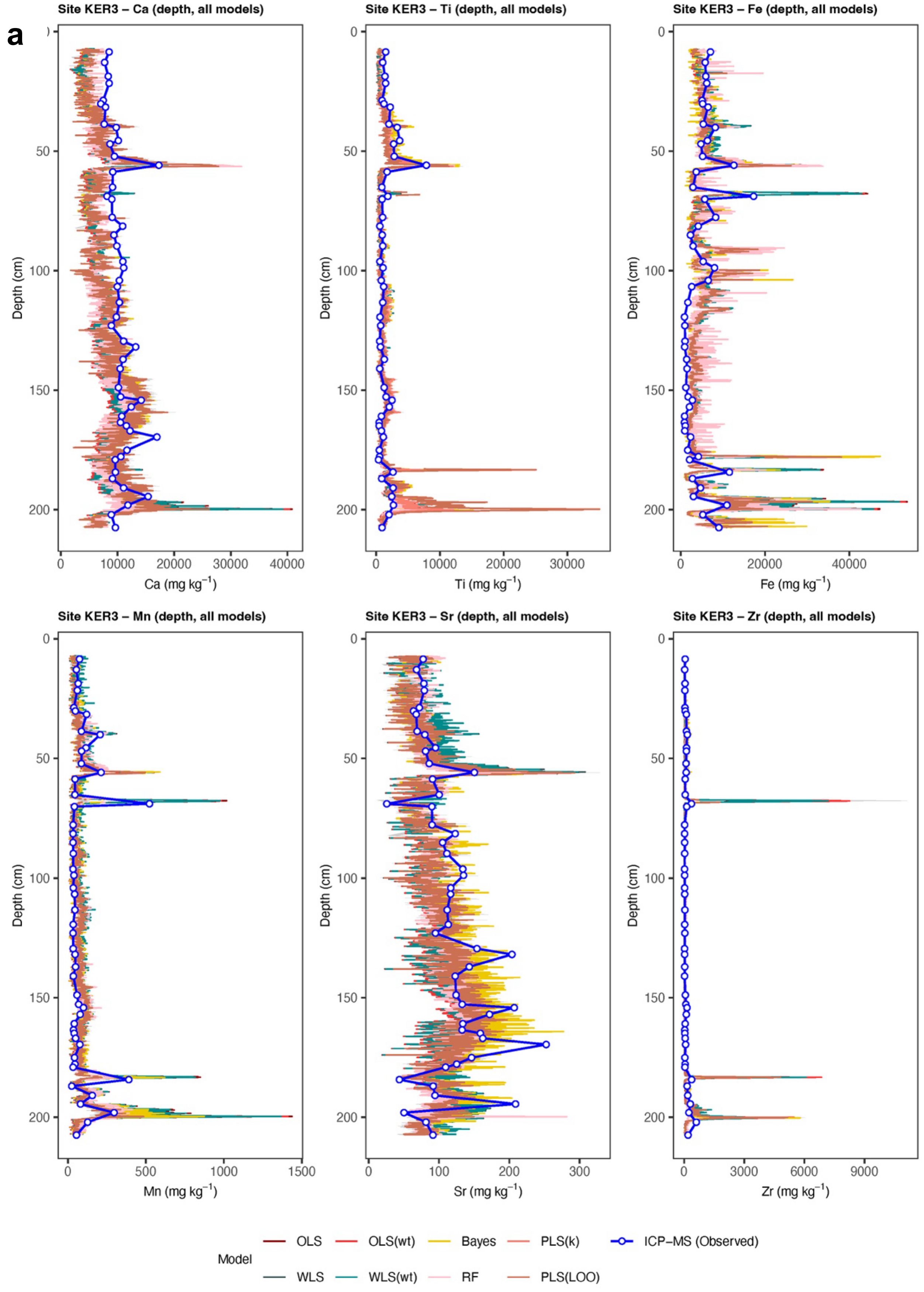

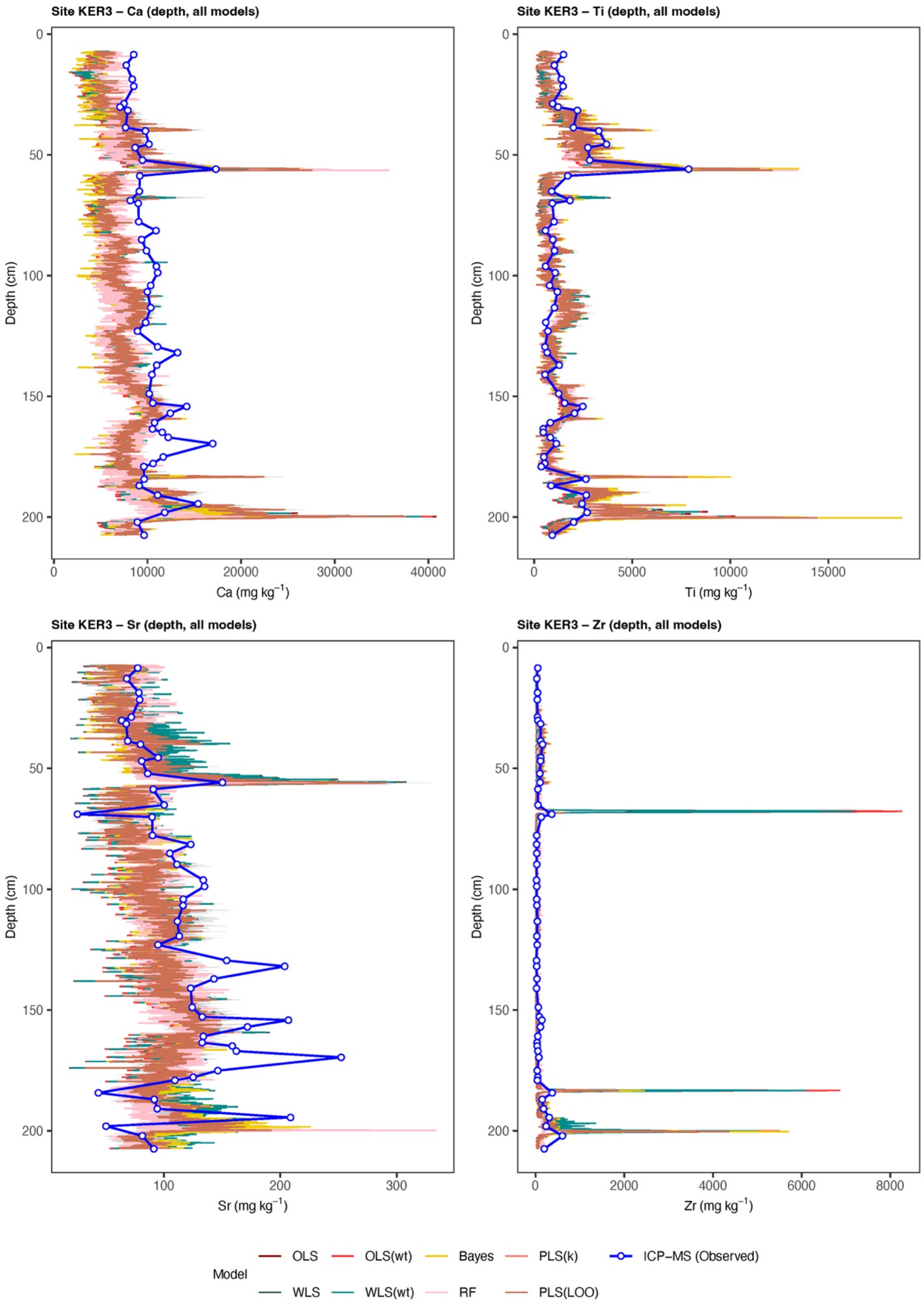

Site KER3 – Ca (depth, all models)
Site KER3 – Ti (depth, all models)
Site KER3 – Sr (depth, all models)
Site KER3 – Zr (depth, all models)
Depth (cm)
Ca (mg kg−1)
Ti (mg kg−1)
Sr (mg kg−1)
Zr (mg kg−1)
Model
OLS
OLS(wt)
Bayes
PLS(k)
ICP−MS (Observed)
WLS
WLS(wt)
RF
PLS(LOO)

Supplementary Figure S17 Peatland record PB1 predicted downcore predicted concentration plots generated by **(a)** 6-element (Ca, Ti, Fe, Mn, Sr, Zr) and for **(b)** 4-element (Ca, Ti, Sr, Zr) univariate (OLS, WLS) and multivariate (Bayes glm, RF, PLS-LOO, PLS-kfold) calibration model runs compared with measured ICP-MS data (blue points/lines).

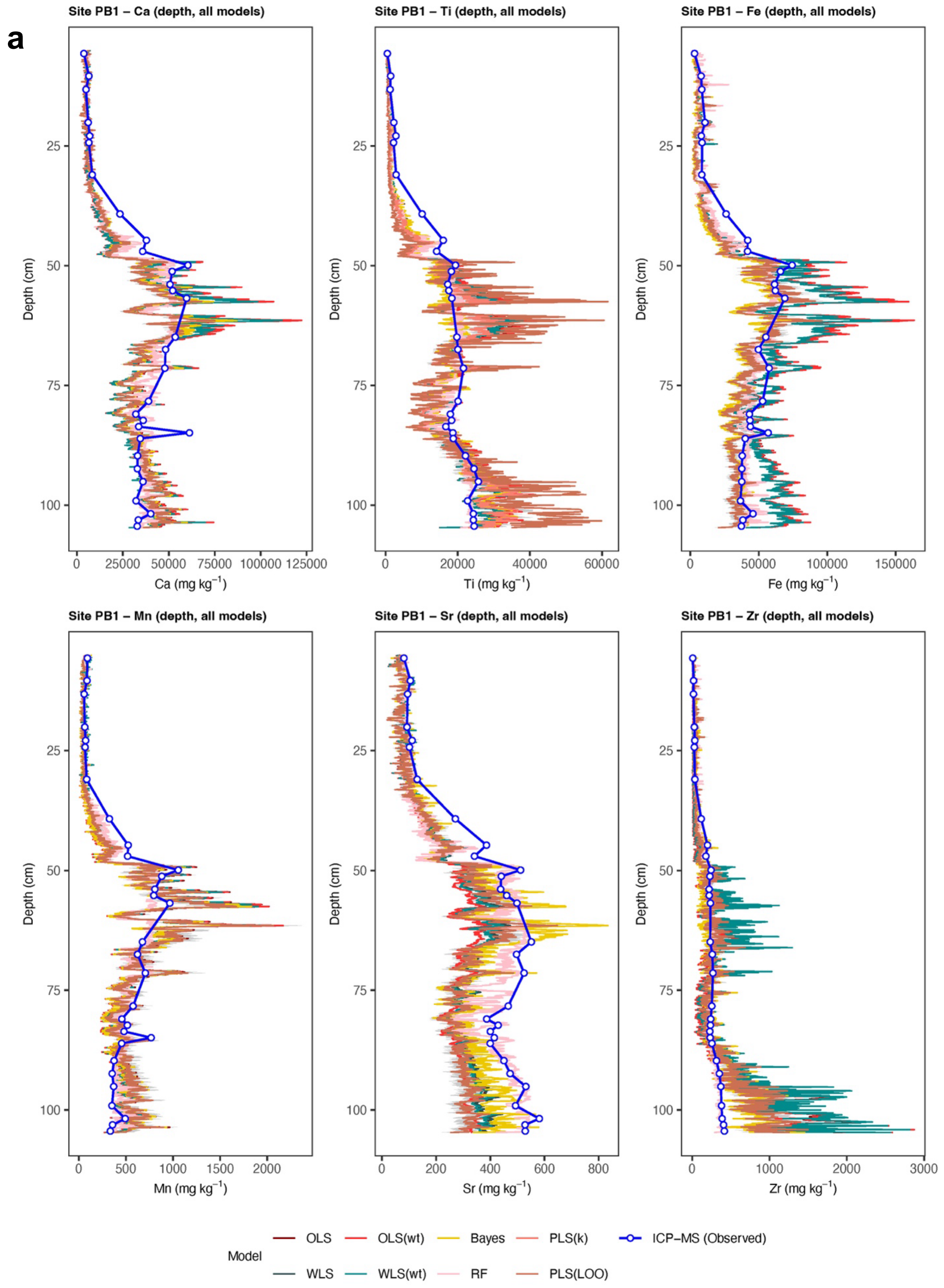

b

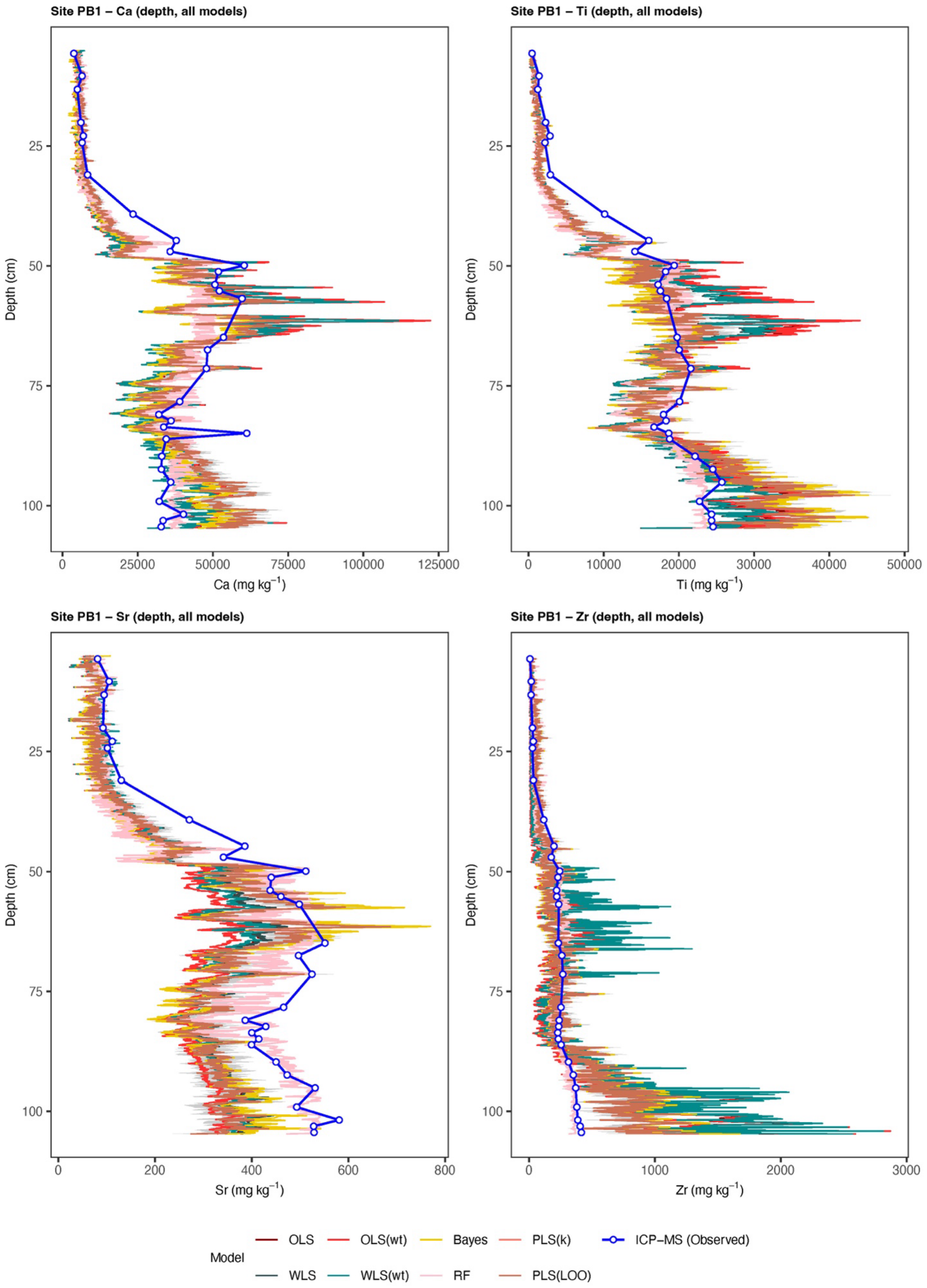

Site PB1 – Ca (depth, all models)
Site PB1 – Ti (depth, all models)
Site PB1 – Sr (depth, all models)
Site PB1 – Zr (depth, all models)
Depth (cm)
Ca (mg kg^{-1})
Ti (mg kg^{-1})
Sr (mg kg^{-1})
Zr (mg kg^{-1})
Model
OLS
OLS(wt)
Bayes
PLS(k)
ICP–MS (Observed)
WLS
WLS(wt)
RF
PLS(LOO)

Supplementary Figure S18 (overpage). (a) Predicted downcore concentrations of Ti with depth produced by the 4-element (Ca, Ti, Sr, Zr) optimised PLS calibration model, the univariate WLS calibration model, and the measured ICP-MS data for peatland record HER42PB. Predicted 95% confidence intervals are shaded grey in all plots.; **(b)** Predicted downcore concentrations of Ti with depth produced by the 4-element (Ca, Ti, Sr, Zr) optimised PLS calibration model applied to the log and clr datasets, and the measured ICP-MS data for peatland record HER42PB; **(c)** Predicted downcore concentrations of Zr with depth produced by the 4-element (Ca, Ti, Sr, Zr) optimised PLS calibration model, the univariate WLS calibration model, and the measured ICP-MS data for peatland record HER42PB; **(d)** As (a) but for site BI10; **(e)** As (b) but for site BI10; **(f)** As (c) but for site BI10; **(g)** As (a) but site KER1; **(h)** As (b) but for site KER1; **(i)** As (c) but for site KER1; **(j)** As (a) but site KER3; **(k)** As (b) but for site KER3; **(l)** As (c) but for site KER3; **(m)** As (a) but for site PB1; **(n)** As (b) but for site PB1; **(o)** As (c) but for site PB1. Data was analysed using the pls and compositions packages in R[5,6].

a-f

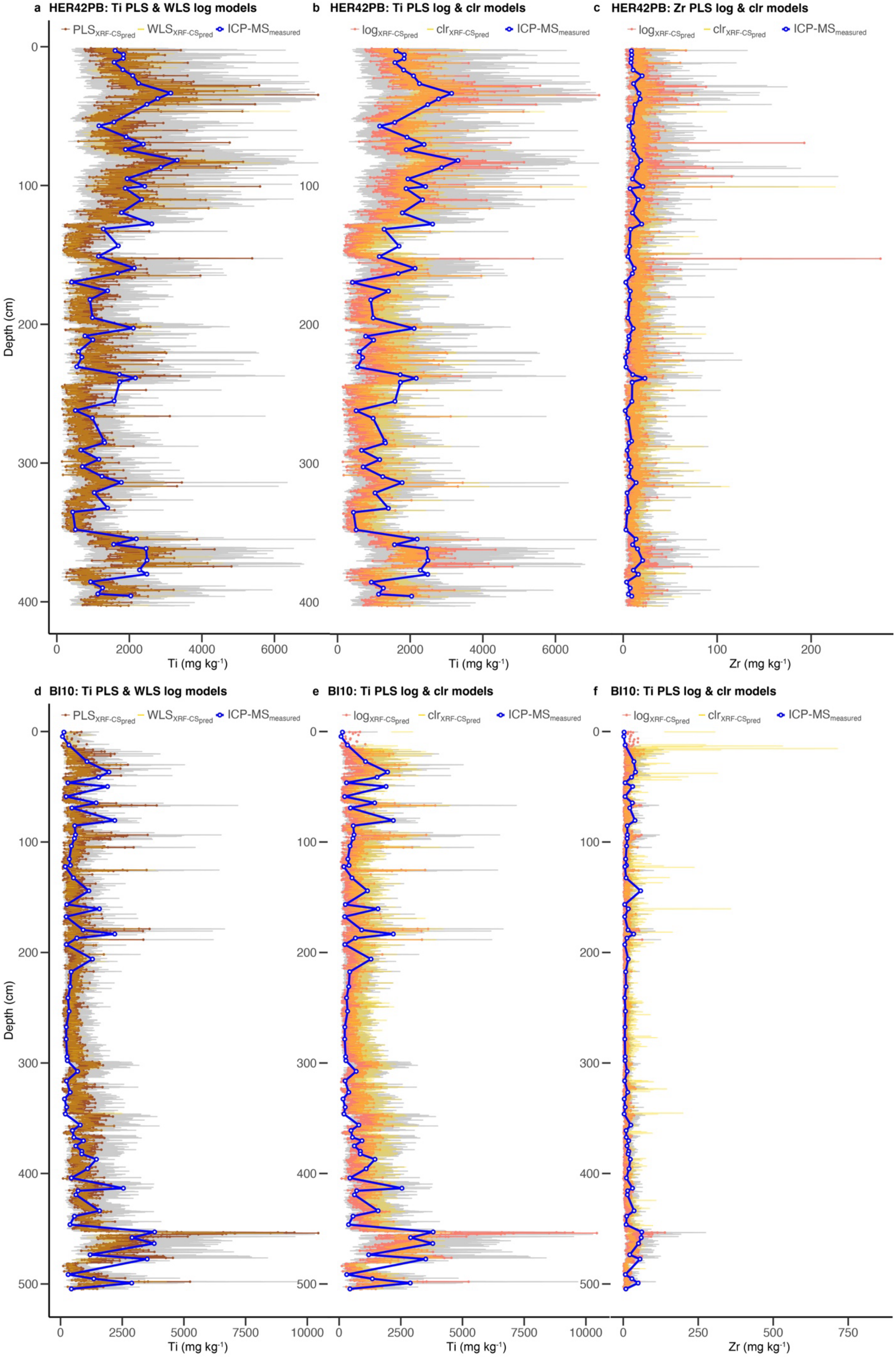

a HER42PB: Ti PLS & WLS log models
b HER42PB: Ti PLS log & clr models
c HER42PB: Zr PLS log & clr models
PLS$_{XRF-CSpred}$
WLS$_{XRF-CSpred}$
ICP-MS$_{measured}$
log$_{XRF-CSpred}$
clr$_{XRF-CSpred}$
Depth (cm)
Ti (mg kg^{-1})
Zr (mg kg^{-1})
d BI10: Ti PLS & WLS log models
e BI10: Ti PLS log & clr models
f BI10: Ti PLS log & clr models

g-l

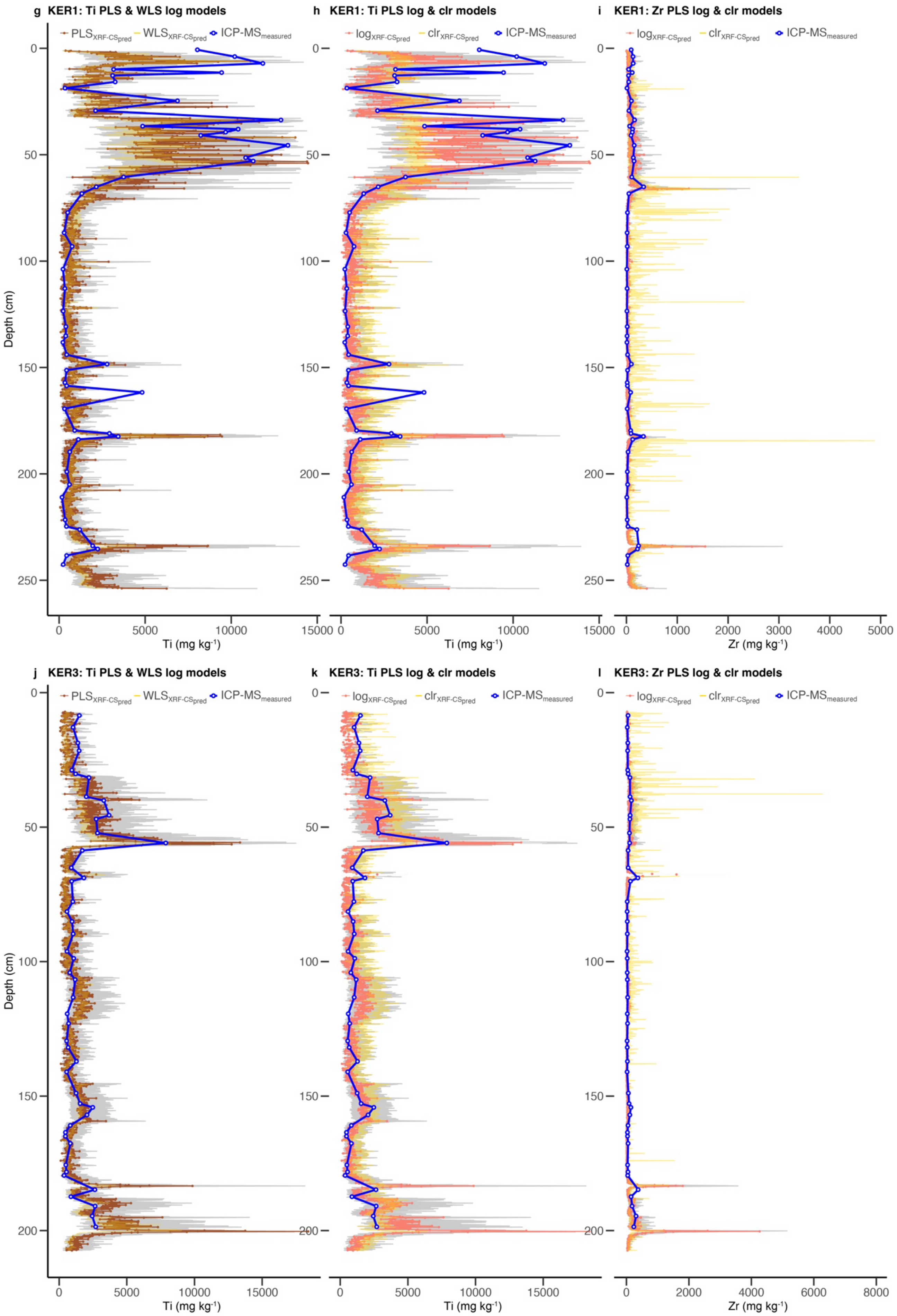
g KER1: Ti PLS & WLS log models
h KER1: Ti PLS log & clr models
i KER1: Zr PLS log & clr models
j KER3: Ti PLS & WLS log models
k KER3: Ti PLS log & clr models
l KER3: Zr PLS log & clr models
PLSXRF-CSpred
WLSXRF-CSpred
ICP-MSmeasured
logXRF-CSpred
clrXRF-CSpred
Depth (cm)
Ti (mg kg-1)
Zr (mg kg-1)

m-o

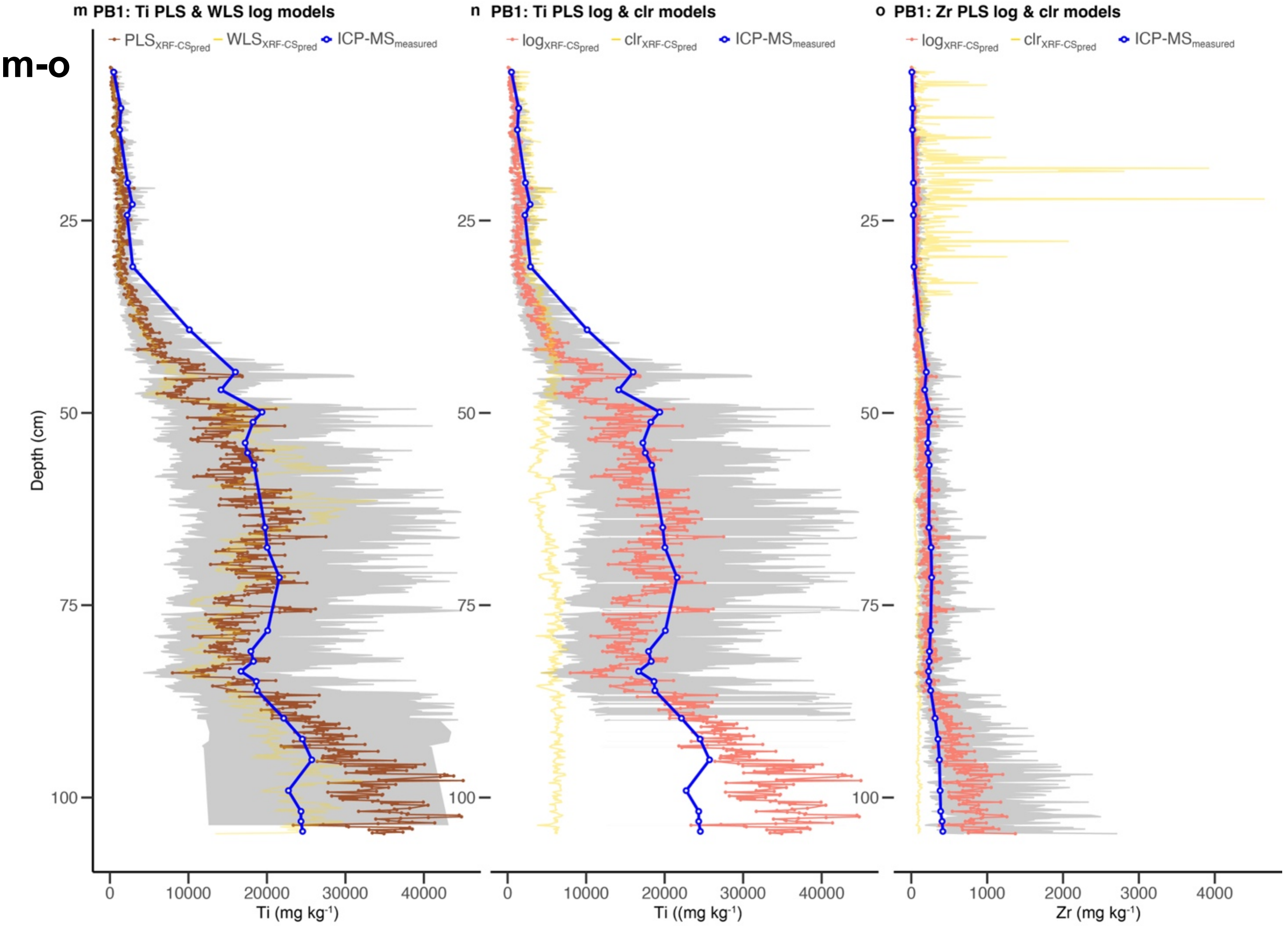

m PB1: Ti PLS & WLS log models
PLSXRF-CSpred
WLSXRF-CSpred
ICP-MSmeasured
Depth (cm)
25
50
75
100
0
10000
20000
30000
40000
Ti (mg kg-1)
n PB1: Ti PLS log & clr models
logXRF-CSpred
clrXRF-CSpred
ICP-MSmeasured
Ti ((mg kg-1)
o PB1: Zr PLS log & clr models
logXRF-CSpred
clrXRF-CSpred
ICP-MSmeasured
0
1000
2000
3000
4000
Zr (mg kg-1)

Supplementary Figure S19. Preliminary age depth models for: **(a)** BI10, **(b)** HER42PB, **(c)** KER1, **(d)** KER3, and **(e)** PB1 peat records produced in rbacon v. 3.3.1[16]. Chronological and dust flux data will be presented and discussed further in a forthcoming paper.

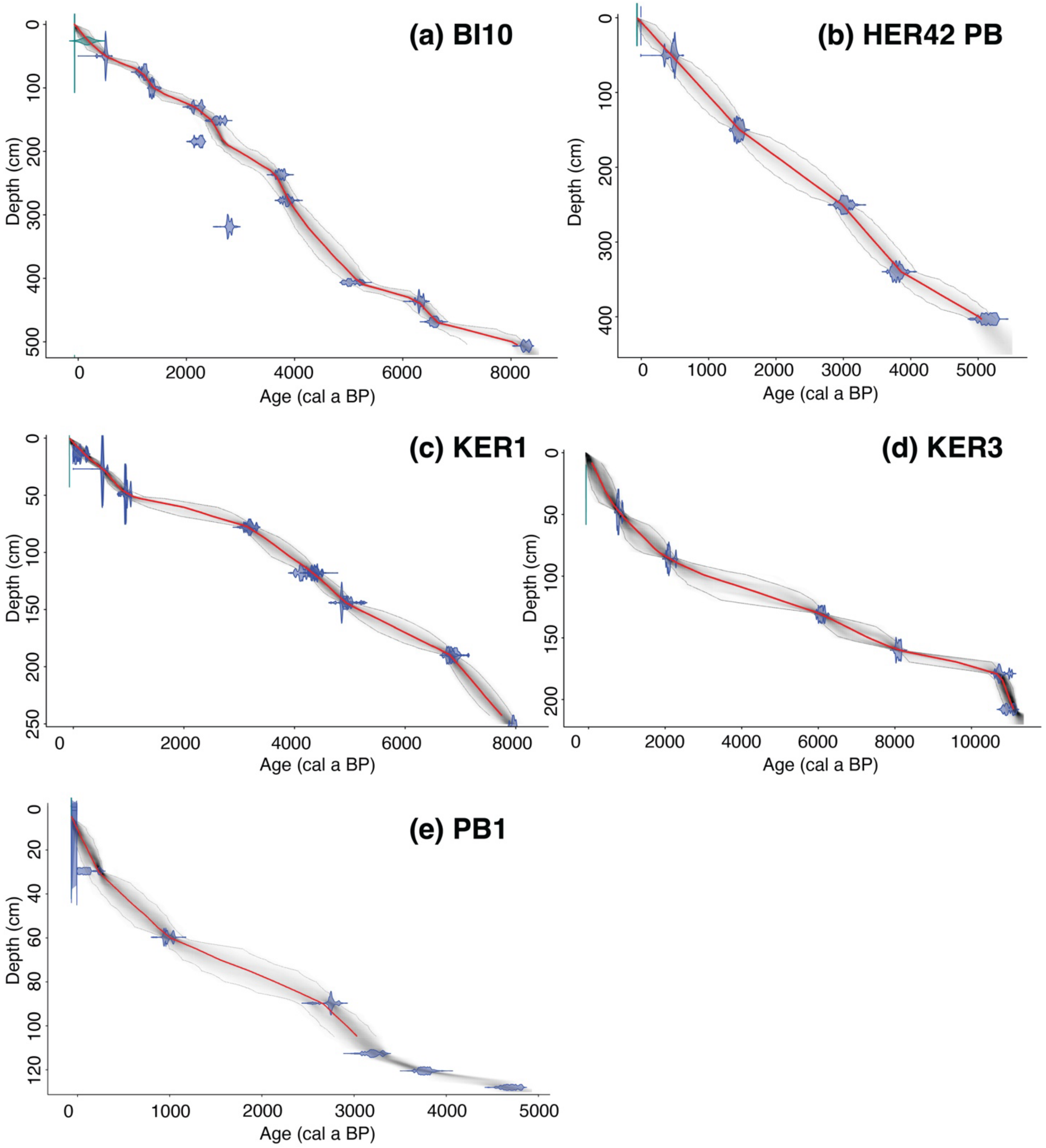

Supplementary Table S1 Accuracy and reproducibility for ICP-MS measurements based on Certified Reference Materials (CRM).

Standard, statistic	K	Ca	Ti	Mn	Fe	Co	Ni	Cu	Zn	Rb	Sr	Zr
**NIST1547a (n=7)**	24300	15600	-	98.0	218	0.07	0.69	3.70	17.9	19.7	53.0	-
Mean (x̄)	19268	13725	-	88.7	229	0.07	1.06	3.43	19.2	16.9	53.8	-
Stdev (σ)	3974	3521	-	21.6	86.0	0.03	0.38	0.78	4.40	3.02	12.8	-
Accuracy (%)	21	12	-	10	5	2	54	7	7	14	2	-
Reproducibility (%)	79	74	-	76	62	64	64	77	77	82	76	-
**NIST1515 (n=6)**	16100	15260	16.5	54.0	83.0	0.09	0.91	5.64	12.5	10.2	25.0	-
Mean (x̄)	12860	13390	17.3	51.4	54.4	0.09	1.40	5.57	13.7	8.73	24.4	-
Stdev (σ)	3074	1705	10.4	6.26	56.8	0.01	0.63	0.97	2.92	0.93	3.41	-
Accuracy (%)	20	12	5	5	34	2	54	1	9	14	2	-
Reproducibility (%)	78	87	84	89	80	87	80	90	79	94	87	-
**GBW-07603 (n=7)**	9200	16800	95.0	61.0	1070	0.41	1.70	6.60	55.0	4.50	246	-
Mean (x̄)	8105	16085	103	65.2	976	0.43	1.99	6.04	58.7	3.85	244	-
Stdev (σ)	699	3177	25.8	7.40	110	0.06	1.05	0.66	11.8	0.41	58.3	-
Accuracy (%)	12	4	8	7	9	5	17	9	7	14	1	-
Reproducibility (%)	91	80	75	89	89	86	47	89	80	89	76	-
**IPE-176 (n=5)**	13000	4160	707	113	6410	2.36	-	10.0	40.2	21.0		-
Mean (x̄)	11482	4310	542	101	5460	1.97	-	8.18	38.4	25.0		-
Stdev (σ)	3260	2681	200	27.1	1542	0.57	-	1.99	10.75	7.19		-
Accuracy (%)	12	4	23	11	15	17	-	18	4	19		-
Reproducibility (%)	72	38	63	73	72	71	-	76	72	71		-
**NJV 94-1 (n=5)**	-	10200	-	36.0	-	-	-	2.00	9.00	-	-	-
Mean (x̄)	-	7772	-	28.5	-	-	-	1.41	10.5	-	-	-
Stdev (σ)	-	705	-	1.20	-	-	-	0.10	8.71	-	-	-
Accuracy (%)	-	24	-	21	-	-	-	29	17	-	-	-
Reproducibility (%)	-	91	-	96	-	-	-	93	17	-	-	-
**Across-CRM average**												
Accuracy (%)	18	13	14	12	18	7	54	14	9	16	2	-
Reproducibility (%)	80	74	74	84	76	77	64	85	65	84	80	-

Supplementary Table S2. Summary statistics (mean, median, min., max., 25th and 75th quartiles) for six key elements, scatter parameters (inc. and coh.), dry mass (DM), Total cps and MSE (Mean Square Error fit of measured to modelled spectra) for the matched XRF-CS - ICP-MS dataset. Data shown horizontally are mean counts per second (cps), standard deviations (SD) as cps and percentages (%), and standard errors (SE) percentages for matched ICP-MS min.-max. subsample depth ranges, derived originally from n=14,513 XRF-CS spectra).

	K (cps)	K SD	K SD %	K SE %	Ca (cps)	Ca SD	Ca SD %	Ca SE %
**Mean**	44	20	65	3.9	376	74	19	1.1
**Median**	28	13	54	3.3	260	40	16	1.0
**Min.**	2	3	15	0.9	42	12	6	0.4
**Max.**	612	116	234	14	2910	612	108	6.5
**Q25th**	13	9	36	2.2	194	29	13	0.8
**Q75th**	55	21	86	5.2	372	64	22	1.3

	Ti (cps)	Ti SD	Ti SD %	Ti SE %	Mn (cps)	Mn SD	Mn SD %	Mn SE %
**Mean**	392	86	39	2.4	81	27	41	2.5
**Median**	93	29	35	2.1	52	21	40	2.4
**Min.**	8	6	8	0.5	13	8	9	0.5
**Max.**	5080	1151	138	8.3	720	411	125	7.6
**Q25th**	52	20	25	1.5	36	17	28	1.7
**Q75th**	199	71	48	2.9	104	30	52	3.1

	Fe (cps)	Fe SD	Fe SD %	Fe SE %	Rb (cps)	Rb SD	Rb SD %	Rb SE %
**Mean**	10275	1833	20	1.2	67	55	112	6.7
**Median**	5634	1024	17	1.0	50	52	107	6.5
**Min.**	601	55	4	0.3	3	5	11	0.7
**Max.**	54932	9924	121	7.3	447	167	282	17
**Q25th**	2562	412	11	0.7	32	41	74	4.5
**Q75th**	15624	2789	24	1.4	87	65	142	8.6

	Sr (cps)	Sr SD	Sr SD %	Sr SE %	Inc	Inc SD	Inc SD %	Inc SE %
**Mean**	584	120	37	2.2	35303	1233	4	0.2
**Median**	400	103	25	1.5	37219	946	3	0.2
**Min.**	2	7	5	0.3	12692	230	1	0.0
**Max.**	2774	448	346	20.9	45281	6266	20	1.2
**Q25th**	219	80	17	1.0	31055	593	2	0.1
**Q75th**	754	137	43	2.6	41850	1492	5	0.3

	Coh	Coh SD	Coh SD %	Coh SE %	% DM	% DM err.	Total cps	MSE
**Mean**	5736	194	4	0.2	22.75	2.27	56782	1.35
**Median**	5899	175	3	0.2	19.69	1.97	55432	1.34
**Min.**	3692	91	1	0.1	5.70	0.57	38366	1.24
**Max.**	6783	573	20	0.6	70.39	7.04	90008	1.52
**Q25th**	5381	143	2	0.1	15.13	1.51	52467	1.31
**Q75th**	6234	214	5	0.2	27.18	2.72	59818	1.38

Supplementary Table S3 Repeat measurement error assessment for XRF-CS of a peat matrix similar to those presented in this study.

**Marion Island MI1 peat record**			**XRF-CS counts per second (cps)**															
Section	n	Position (mm)	Ca	Ca SD	Ti	Ti SD	Mn	Mn SD	Fe	Fe SD	Sr	Sr SD	Zr	Zr SD	inc	inc SD	coh	coh SD
MI1A	1	36	60	200	20	300	0	0	4080	800	100	2000	250	700	70300	5000	10100	1000
MI1A	2	37	48	80	20	100	0	0	4420	200	100	400	130	800	70000	4000	9960	700
MI1A	3	38	70	300	30	200	0	0	4230	800	30	400	300	1000	69400	5000	10000	2000
MI1A	4	39	64	20	20	300	0	0	3900	1000	10	200	290	900	68690	700	10020	600
MI1A	5	40	60	100	6	80	0	0	3200	1000	5	70	280	500	68300	1000	10300	1000
MI1A	6	41	90	200	12	90	0	0	2442	70	80	300	100	1000	68300	4000	10100	1000
MI1A	7	42	80	200	10	100	0	0	2590	600	180	300	180	400	70300	1000	10280	200
MI1A	8	43	90	200	19	70	3	40	3890	300	60	800	180	300	69400	3000	10350	600
MI1A	9	44	90	100	20	100	0	0	6560	500	70	1000	30	200	69000	6000	10100	1000
MI1A	10	45	105	60	30	100	0	0	6600	1000	0	0	106	20	67800	2000	9800	2000
MI1A	11	46	92	60	30	400	0	0	9800	1000	140	100	90	500	68000	6000	9860	700
MI1A	12	47	100	200	60	200	0	0	13690	500	20	300	180	600	67400	4000	9870	300
Rest of cores 1A-1J omitted - data available online: https://github.com/stever60/ACE_peat_calibration/tree/main/2024_DeVleeschouwer/ACE_Errors/Data/Output/Repeat_err/Marion																		
MI1J	363	1157	1940	80	9800	100	41	4	31300	300	1980	70	1030	60	37000	300	7840	4
MI1J	364	1158	1688	2	9400	100	30	20	30410	30	1640	80	890	80	36500	500	7600	100
MI1J	365	1159	1945.5	7	9160	20	40	10	28900	100	2070	30	900	200	34900	200	7200	200
MI1J	366	1160	2290	20	9800	300	60	9	28880	70	2080	40	844	8	32350	80	7070	10
MI1J	367	1161	2680	20	8760	50	102	2	29240	20	2200	100	990	40	29100	300	6900	100
MI1J	368	1162	2510	30	7360	50	70	10	24200	200	2340	40	900	100	26224	9	6600	100
MI1J	369	1163	3143	7	8700	100	167.5	7	30210	4	2390	40	1020	60	25300	200	6400	100
MI1J	370	1164	2400	20	7100	200	120	10	25220	20	2300	100	940	70	26300	200	6400	100
MI1J	371	1165	2900	100	8290	90	110	6	32200	500	2440	10	960	40	27000	100	6700	200
MI1J	372	1166	2950	10	8100	100	140	20	33181	8	2580	80	980	60	29000	600	7200	100
MI1J	373	1167	2600	100	7810	10	100	30	29600	200	2400	100	860	30	30900	300	7180	30
MI1J	374	1168	2010	40	7410	80	80	30	23800	200	2150	20	860	40	32200	700	7200	200
		**Mean cps**	**238**		**812**		**6**		**7156**		**239**		**173**		**67574**		**9776**	
		**Mean cps +/- SD**	**439**		**1704**		**18**		**7148**		**447**		**236**		**9592**		**763**	
		**Mean cps +/- SE (n=2)**	**310**		**1205**		**12.6**		**5054**		**316**		**167**		**6782**		**539**	
		**Mean cps +/- %SE (n=2)**	**131**		**148**		**207**		**70.6**		**132**		**96.6**		**10.04**		**5.52**	
		**Mean SD**		**28**		**39**		**4**		**174**		**99**		**96**		**651**		**249**
		**Mean SE**		**1.4**		**2.0**		**0.2**		**9.0**		**5.1**		**5.0**		**33.6**		**12.9**
		**Mean % SD (n=374)**		**11.8**		**4.8**		**64.7**		**2.4**		**41.6**		**55.5**		**0.96**		**2.54**
		**Mean %SE (n=374)**		**0.6**		**0.2**		**3.3**		**0.1**		**2.2**		**2.9**		**0.05**		**0.13**
		**Min. (n=374)**	**48**	**0**	**6**	**0**	**0**	**0**	**540**	**0**	**0**	**0**	**0**	**0**	**25300**	**4**	**6400**	**1**
		**Max. (n=374)**	**3143**	**300**	**9800**	**500**	**167.5**	**40**	**46200**	**7000**	**2580**	**2000**	**1030**	**2000**	**75500**	**8000**	**10700**	**5000**

Supplementary Table S4 Summary accumulation rate, water content, dry mass, organic Carbon, dry density and Dry Mass Accumulation Rate (DMAR) statistics for subsamples in this study. (a) For the whole ACE ~1 cm interval subsample dataset (n = 1149), and for each of the five peatland sites (b) BI10, (c) HER42PB, (d) KER1, (e) KER3, (f) PB1) individually.

(a) Site	Parameter	Units	n	Mean	1σ	Median	Trim Mean	Median Abs. Dev.	Min.	Max.	Range	Skewness	Kurtosis	Standard Error	Lower quartile (Q0.25)	Upper Quartile (Q0.75)
ACE	Acc. rate	cm yr^{-1}	1144	0.07	0.07	0.06	0.07	0.04	0.01	1.28	1.27	10.34	170.22	0.002	0.04	0.09
ACE	Acc. rate error	cm yr^{-1}	1143	0.01	0.14	0.01	0.01	0.01	-3.17	2.37	5.55	-5.52	289.19	0.004	0	0.01
ACE	Water Content	%	1149	79.1	10.12	82.35	80.98	5.72	21.99	94.75	72.76	-1.98	4.46	0.299	76.56	85.53
ACE	Water Content error	%	1149	3.95	0.51	4.11	4.05	0.29	1.1	4.8	3.7	-1.98	4.44	0.015	3.83	4.27
ACE	Dry mass	%	1149	20.9	10.12	17.65	19.02	5.72	5.25	78.01	72.76	1.98	4.46	0.299	14.47	23.44
ACE	Dry mass error	%	1149	1.05	0.51	0.88	0.95	0.28	0.26	3.9	3.64	1.98	4.46	0.015	0.72	1.17
ACE	C org pc	%	1055	37.79	12.75	39.53	38.72	11.42	1.18	58.65	57.47	-0.56	-0.38	0.393	30.3	45.52
ACE	C org pc error	%	1055	3.47	1.15	3.85	3.6	1.02	0.15	5.07	4.92	-0.87	-0.33	0.036	2.77	4.36
ACE	Dry density	g cm^{-3}	1149	0.31	0.19	0.24	0.27	0.11	0.02	1.58	1.56	2.07	5.65	0.006	0.18	0.38
ACE	Dry density error	g cm^{-3}	1149	0.52	0.44	0.4	0.46	0.31	0	4.86	4.85	2.34	11.88	0.013	0.22	0.69
ACE	DMAR	g cm^{-2} yr^{-1}	1144	0.019	0.017	0.017	0.017	0.011	0.001	0.326	0.325	6.551	90.695	0.001	0.01	0.025
ACE	DMAR error	g cm^{-2} yr^{-1}	1143	0.038	0.043	0.029	0.034	0.03	-0.405	0.362	0.767	-0.43	24.585	0.001	0.012	0.056

(b) Site	Parameter	Units	n	Mean	1σ	Median	Trim Mean	Median Abs. Dev.	Min.	Max.	Range	Skewness	Kurtosis	Standard Error	Lower quartile (Q0.25)	Upper Quartile (Q0.75)
BI10	Strat. Depth top	cm	421	249.9	145.6	247.5	249.3	186.7	0	505.3	505.3	0	-1.2	7.095	123.9	375.8
BI10	Strat. Depth bottom	cm	421	250.9	145.6	248.7	250.3	186.8	1.1	507	505.9	0	-1.2	7.096	124.9	376.9
BI10	Depth	cm	421	250.4	145.6	248.1	249.8	186.7	0.6	506.2	505.6	0	-1.2	7.096	124.4	376.4
BI10	Acc. rate	cm yr^{-1}	420	0.08	0.04	0.09	0.08	0.04	0.02	0.17	0.15	0.33	-0.59	0.002	0.05	0.11
BI10	Acc. rate error	cm yr^{-1}	420	0.02	0.13	0.01	0.01	0.00	-0.79	2.37	3.16	13.96	259	0.006	0.00	0.01
BI10	Water Content	%	421	81.01	5.91	82.29	81.81	4.24	35.37	90.18	54.81	-2.95	14.79	0.288	78.8	84.62
BI10	Water Content error	%	421	4.05	0.3	4.11	4.09	0.22	1.77	4.51	2.75	-2.94	14.7	0.014	3.94	4.24
BI10	Dry mass	%	421	18.99	5.91	17.71	18.19	4.24	9.82	64.63	54.81	2.95	14.79	0.288	15.38	21.2
BI10	Dry mass error	%	421	0.95	0.3	0.89	0.91	0.21	0.49	3.23	2.74	2.95	14.76	0.014	0.77	1.06
BI10	C org pc	%	421	38.04	6.55	39.47	39.15	3.74	1.18	52.34	51.16	-2.35	7.42	0.319	36.75	41.87
BI10	C org pc error	%	421	3.8	0.65	3.95	3.92	0.37	0.15	4.71	4.56	-2.38	7.47	0.032	3.68	4.19
BI10	Dry density	g cm^{-3}	421	0.22	0.08	0.21	0.21	0.05	0.09	1	0.92	3.98	29.02	0.004	0.18	0.25
BI10	Dry density error	g cm^{-3}	421	0.65	0.54	0.51	0.59	0.47	0.01	4.86	4.85	2.25	10.78	0.026	0.28	0.98
BI10	DMAR	g cm^{-2} yr^{-1}	420	0.018	0.009	0.018	0.017	0.01	0.004	0.074	0.07	0.984	3.064	0.000	0.011	0.024
BI10	DMAR error	g cm^{-2} yr^{-1}	420	0.053	0.043	0.045	0.048	0.04	-0.067	0.349	0.415	1.818	7.09	0.002	0.022	0.076

(c) Site	Parameter	Units	n	Mean	1σ	Median	Trim Mean	Median Abs. Dev.	Min.	Max.	Range	Skew-ness	Kurtosis	Standard Error	Lower quartile (Q0.25)	Upper Quartile (Q0.75)
HER42PB	Strat. Depth top	cm	305	203.4	118.5	204	203.2	151.8	0	409.6	409.6	0	-1.2	6.783	101.6	305.1
HER42PB	Strat. Depth bottom	cm	305	204.6	118.5	205.2	204.3	151.4	1	410.3	409.3	0	-1.2	6.784	103.1	306.3
HER42PB	Depth	cm	305	204	118.5	204.6	203.8	151.5	0.5	410	409.5	0	-1.2	6.784	102.4	305.7
HER42PB	Acc. rate	cm yr^{-1}	304	0.09	0.05	0.09	0.08	0.02	0.05	0.41	0.37	5.01	28.27	0.003	0.07	0.1
HER42PB	Acc. rate error	cm yr^{-1}	303	0.02	0.08	0.01	0.01	0.01	-0.65	0.89	1.54	2.99	73.75	0.004	0.01	0.02
HER42PB	Water Content	%	305	77.74	8.42	79.98	78.8	7.67	29.61	89.24	59.63	-1.38	3.18	0.482	73.13	84.45
HER42PB	Water Content error	%	305	3.89	0.42	3.99	3.94	0.38	1.48	4.48	3	-1.38	3.15	0.024	3.65	4.22
HER42PB	Dry mass	%	305	22.26	8.42	20.02	21.2	7.67	10.76	70.39	59.63	1.38	3.18	0.482	15.55	26.87
HER42PB	Dry mass error	%	305	1.11	0.42	1	1.06	0.39	0.54	3.52	2.98	1.39	3.19	0.024	0.78	1.34
HER42PB	C org pc	%	305	27.91	10.68	27.2	27.8	13.42	5.43	54.31	48.88	0.1	-1.1	0.612	18.72	36.87
HER42PB	C org pc error	%	305	2.41	0.92	2.35	2.4	1.16	0.48	4.68	4.2	0.1	-1.1	0.053	1.62	3.18
HER42PB	Dry density	g cm^{-3}	305	0.31	0.15	0.27	0.29	0.13	0.14	1.01	0.88	1.42	2.3	0.008	0.2	0.38
HER42PB	Dry density error	g cm^{-3}	305	0.53	0.37	0.44	0.47	0.21	0.03	2.04	2.02	1.9	4.06	0.021	0.32	0.61
HER42PB	DMAR	g cm^{-2} yr^{-1}	304	0.029	0.026	0.023	0.025	0.011	0.007	0.326	0.319	6.256	60.359	0.001	0.017	0.032
HER42PB	DMAR error	g cm^{-2} yr^{-1}	303	0.048	0.036	0.045	0.047	0.021	-0.229	0.362	0.591	0.073	31.542	0.002	0.031	0.06

Site	Parameter	Units	n	Mean	1σ	Median	Trim Mean	Median Abs. Dev.	Min.	Max.	Range	Skewness	Kurtosis	Standard Error	Lower quartile (Q0.25)	Upper Quartile (Q0.75)
KER1	Strat. Depth top	cm	171	126.7	74	127.4	126.6	95.8	0	253.5	253.5	0	-1.2	5.662	62.1	189.8
KER1	Strat. Depth bottom	cm	171	128	74	128.6	127.9	95.5	1.4	254.5	253.1	0	-1.2	5.659	63.5	191.1
KER1	Depth	cm	171	127.4	74	128	127.3	95.6	0.7	254	253.3	0	-1.2	5.66	62.8	190.5
KER1	Acc. rate	cm yr^{-1}	170	0.04	0.02	0.04	0.04	0.02	0.01	0.07	0.07	-0.24	-1.14	0.001	0.02	0.06
KER1	Acc. rate error	cm yr^{-1}	170	0	0.06	0	0	0	-0.71	0.34	1.04	-7.64	99.44	0.005	0	0
KER1	Water Content	%	171	80.67	10.88	85.67	82.91	4.03	41.4	93.1	51.7	-1.71	2.18	0.832	77.95	87.72
KER1	Water Content error	%	171	4.04	0.55	4.29	4.15	0.2	2.07	4.72	2.65	-1.7	2.17	0.042	3.9	4.39
KER1	Dry mass	%	171	19.33	10.88	14.33	17.09	4.03	6.9	58.6	51.7	1.71	2.18	0.832	12.28	22.05
KER1	Dry mass error	%	171	0.97	0.54	0.72	0.85	0.21	0.35	2.93	2.58	1.71	2.18	0.042	0.61	1.1
KER1	C org pc	%	171	44.34	16.2	53.67	47.31	2.24	4.47	58.65	54.18	-1.31	0.12	1.239	37.45	54.65
KER1	C org pc error	%	171	3.84	1.4	4.64	4.09	0.19	0.4	5.07	4.67	-1.31	0.12	0.107	3.25	4.73
KER1	Dry density	g cm^{-3}	171	0.49	0.2	0.43	0.45	0.09	0.2	1.58	1.37	2.62	8.07	0.015	0.38	0.5
KER1	Dry density error	g cm^{-3}	171	0.51	0.3	0.49	0.48	0.32	0.02	1.74	1.72	0.92	1.45	0.023	0.28	0.71
KER1	DMAR	g cm^{-2} yr^{-1}	170	0.02	0.013	0.016	0.017	0.008	0.005	0.086	0.08	2.347	7.202	0.001	0.011	0.023
KER1	DMAR error	g cm^{-2} yr^{-1}	170	0.023	0.043	0.018	0.022	0.014	-0.405	0.246	0.651	-4.61	60.982	0.003	0.011	0.037

Site	Parameter	Units	n	Mean	1σ	Median	Trim Mean	Median Abs. Dev.	Min.	Max.	Range	Skewness	Kurtosis	Standard Error	Lower quartile (Q0.25)	Upper Quartile (Q0.75)
KER3	Strat. Depth top	cm	158	105.6	59.7	105.5	105.8	74.9	0	208.3	208.3	0	-1.2	4.748	55.6	156.1
KER3	Strat. Depth bottom	cm	158	106.7	59.6	106.6	106.9	74.9	1.2	210	208.8	0	-1.2	4.745	56.9	157.2
KER3	Depth	cm	158	106.2	59.7	106.1	106.3	74.9	0.6	209.2	208.6	0	-1.2	4.746	56.2	156.7
KER3	Acc. rate	$cm\ yr^{-1}$	157	0.03	0.02	0.02	0.03	0.02	0.01	0.08	0.08	0.57	-1.16	0.002	0.01	0.05
KER3	Acc. rate error	$cm\ yr^{-1}$	157	0.01	0.1	0	0.01	0	-1.08	0.35	1.43	-8.97	104.96	0.008	0	0.01
KER3	Water Content	%	158	82.79	7.46	84.97	84.13	3.2	38.22	94.3	56.08	-3.69	17.47	0.594	81.07	86.69
KER3	Water Content error	%	158	4.14	0.37	4.25	4.21	0.17	1.91	4.8	2.89	-3.65	17.3	0.03	4.06	4.33
KER3	Dry mass	%	158	17.21	7.46	15.04	15.87	3.2	5.7	61.78	56.08	3.69	17.47	0.594	13.31	18.93
KER3	Dry mass error	%	158	0.86	0.37	0.75	0.79	0.16	0.29	3.09	2.8	3.68	17.47	0.03	0.67	0.95
KER3	C org pc	%	158	49.11	10.33	52.61	51.46	3.43	4.53	57.8	53.27	-2.57	6.54	0.822	49.1	54.65
KER3	C org pc error	%	158	4.25	0.89	4.55	4.45	0.3	0.4	5	4.6	-2.56	6.53	0.071	4.25	4.73
KER3	Dry density	$g\ cm^{-3}$	158	0.2	0.12	0.18	0.18	0.05	0.07	1.08	1.02	4.54	25.2	0.01	0.15	0.22
KER3	Dry density error	$g\ cm^{-3}$	158	0.23	0.26	0.17	0.18	0.13	0.01	1.99	1.98	4.05	20.67	0.021	0.09	0.28
KER3	DMAR	$g\ cm^{-2}\ yr^{-1}$	157	0.008	0.011	0.004	0.006	0.003	0.001	0.078	0.077	3.867	18.233	0.001	0.002	0.009
KER3	DMAR error	$g\ cm^{-2}\ yr^{-1}$	157	0.01	0.025	0.004	0.007	0.003	-0.155	0.144	0.299	0.534	21.205	0.002	0.003	0.009

Site	Parameter	Units	n	Mean	1σ	Median	Trim Mean	Median Abs. Dev.	Min.	Max.	Range	Skewness	Kurtosis	Standard Error	Lower quartile (Q0.25)	Upper Quartile (Q0.75)
PB1	Strat. Depth top	cm	94	64.5	36.7	64.9	64.6	46.6	0	127.5	127.5	0	-1.2	3.788	33.4	95.6
PB1	Strat. Depth bottom	cm	94	65.7	36.7	66.1	65.8	46.5	1	130	129	0	-1.2	3.783	34.6	96.7
PB1	Depth	cm	94	65.1	36.7	65.6	65.2	46.5	0.5	128.8	128.3	0	-1.2	3.786	34.1	96.1
PB1	Acc. rate	$cm\ yr^{-1}$	93	0.07	0.18	0.04	0.04	0.03	0.01	1.28	1.27	6.24	38.44	0.018	0.02	0.04
PB1	Acc. rate error	$cm\ yr^{-1}$	93	0.00	0.37	0.00	0.01	0.01	-3.17	1.25	4.42	-6.54	59.27	0.038	0	0.02
PB1	Water Content	%	94	65.84	18.35	61.13	66.06	19.59	21.99	94.75	72.76	0.05	-1.17	1.893	50.72	87.33
PB1	Water Content error	%	94	3.29	0.92	3.06	3.31	0.98	1.1	4.71	3.61	0.05	-1.17	0.095	2.54	4.34
PB1	Dry mass	%	94	34.16	18.35	38.87	33.94	19.59	5.25	78.01	72.76	-0.05	-1.17	1.893	12.68	49.28
PB1	Dry mass error	%	94	1.71	0.92	1.95	1.7	0.98	0.26	3.9	3.64	-0.05	-1.17	0.095	0.63	2.47
PB1	Dry density	$g\ cm^{-3}$	94	0.49	0.3	0.52	0.47	0.38	0.02	1.07	1.04	0.09	-1.27	0.031	0.15	0.7
PB1	Dry density error	$g\ cm^{-3}$	94	0.38	0.32	0.34	0.34	0.42	0	1.16	1.16	0.57	-0.7	0.033	0.05	0.59
PB1	DMAR	$g\ cm^{-2}\ yr^{-1}$	93	0.016	0.013	0.012	0.014	0.008	0.004	0.104	0.1	3.75	21.213	0.001	0.008	0.021
PB1	DMAR error	$g\ cm^{-2}\ yr^{-1}$	93	0.011	0.041	0.012	0.014	0.009	-0.359	0.111	0.47	-7.528	66.351	0.004	0.007	0.02

Supplementary Table S5(a). Summary of key performance indicators for 6 elements (Ca, Fe, Mn, Sr, Ti, Zr) across eight models tested. Multivariate models are shaded grey, with PLS models are shaded dark grey. $RMSEP_{CV}$ is cross validated RMSEP, $RMSEP_b$ is bootstrapped RMSEP (see Supplementary Methods for definitions and details).

**a) Performance**		log-space			Components		Concentration-space (mg kg^{-1})						
**6 El.**	Model	$R^2_{SS\text{-}cv}$	RMSE	RMSEP	AIC	BIC	RMSE	$RMSEP_{cv}$	$RMSEP_b$	$RMSEP_b$ 1σ	Mean obs.	Mean Pred.	Mean 95% CI width
Ca	Bayes	0.718	0.3776	0.3913	254.46	283.19	4920	5140	5275	627	13400	11347	18369
	PLS_LOO	0.715	0.3802	0.3910	-	-	5060	5183	5386	672	13400	11256	18369
	PLS_kfold	0.715	0.3802	0.3910	-	-	5060	5183	5386	672	13400	11256	18369
	RF	0.736	0.3655	0.3712	-	-	4460	4628	4819	448	13400	11055	17229
	OLS_wt	0.601	0.4496	-	359.87	370.64	5621	-	5697	563	13400	10492	18036
	OLS	0.605	0.4470	0.4509	335.01	345.78	5505	5658	5697	563	13400	10921	22039
	WLS_wt	0.605	0.4474	-	321.19	331.96	5447	-	5697	563	13400	10873	114937
	WLS	0.605	0.4474	-	321.22	331.99	5446	-	5697	563	13400	10872	118586
Fe	OLS_wt	0.705	0.6197	-	522.98	533.75	19165	-	19032	3839	21585	15757	26306
	Bayes	0.783	0.5319	0.5534	438.15	466.88	13055	13834	14100	3299	21585	20039	49771
	PLS_LOO	0.770	0.5476	0.5589	-	-	12699	13221	13237	3040	21585	19737	50988
	PLS_kfold	0.770	0.5476	0.5589	-	-	12699	13221	13237	3040	21585	19737	50988
	RF	0.805	0.5040	0.5167	-	-	18096	18403	18739	5978	21585	19334	44723
	OLS	0.712	0.6124	0.6192	503.73	514.50	19046	19195	19032	3839	21585	16490	50728
	WLS_wt	0.711	0.6129	-	491.44	502.21	19121	-	19032	3839	21585	16225	181109
	WLS	0.711	0.6129	-	491.39	502.16	19119	-	19032	3839	21585	16231	190029
Mn	PLS_LOO	0.652	0.5679	0.5838	-	-	106	111	115	17	198	154	417
	PLS_kfold	0.652	0.5679	0.5838	-	-	106	111	115	17	198	154	417
	RF	0.695	0.5317	0.5387	-	-	100	103	105	10	198	158	392
	Bayes	0.675	0.5488	0.5633	454.93	483.66	106	111	120	20	198	154	400
	OLS	0.642	0.5761	0.5785	470.98	481.75	112	114	115	12	198	147	415
	OLS_wt	0.622	0.5926	-	464.26	475.04	115	-	115	12	198	132	940
	WLS_wt	0.642	0.5764	-	452.11	462.88	110	-	115	12	198	146	1673
	WLS	0.642	0.5766	-	449.38	460.16	109	-	115	12	198	145	1631
Sr	RF	0.730	0.3217	0.3277	-	-	43	43	46	4	164	143	192
	Bayes	0.719	0.3280	0.3462	179.05	207.77	50	51	52	6	164	151	207
	PLS_LOO	0.681	0.3493	0.3596	-	-	61	62	63	10	164	150	221
	PLS_kfold	0.681	0.3493	0.3596	-	-	61	62	63	10	164	150	221
	OLS	0.606	0.3884	0.3916	259.70	270.47	60	60	61	5	164	143	243
	WLS	0.606	0.3885	-	259.48	270.25	59	-	61	5	164	143	2054
	WLS_wt	0.605	0.3888	-	262.01	272.78	62	-	61	5	164	142	1720
	OLS_wt	0.588	0.3970	-	330.65	341.42	69	-	61	5	164	132	1069088
Ti	Bayes	0.769	0.5914	0.6237	495.05	523.78	1783	2175	2160	679	3392	2137	6141
	PLS_LOO	0.754	0.6104	0.6419	-	-	2029	2537	2442	721	3392	2134	6414
	RF	0.757	0.6069	0.6090	-	-	1564	1585	1693	222	3392	2185	6513
	OLS	0.730	0.6396	0.6444	527.01	537.78	2036	2124	2183	419	3392	2121	6994
	OLS_wt	0.719	0.6529	-	558.91	569.68	2174	-	2183	419	3392	2011	7272
	PLS_kfold	0.723	0.6482	0.6623	-	-	3631	4038	4120	1500	3392	2282	7490
	WLS_wt	0.729	0.6405	-	488.00	498.77	1893	-	2183	419	3392	2056	22898
	WLS	0.729	0.6408	-	487.49	498.26	1883	-	2183	419	3392	2048	23897
Zr	OLS	0.608	0.8223	0.8294	661.70	672.47	148	171	171	94	65	52	261
	WLS_wt	0.608	0.8226	-	661.82	672.59	163	-	171	94	65	54	385
	Bayes	0.724	0.6910	0.7329	578.46	607.19	78	96	100	48	65	46	166
	RF	0.759	0.6451	0.6569	-	-	45	45	48	11	65	43	139
	PLS_LOO	0.697	0.7230	0.7630	-	-	93	111	115	58	65	49	192
	PLS_kfold	0.697	0.7230	0.7630	-	-	93	111	115	58	65	49	192
	WLS	0.608	0.8225	-	661.73	672.50	159	-	171	94	65	54	577
	OLS_wt	0.401	1.0169	-	788.73	799.50	154	-	171	94	65	42	34663157

Supplementary Table S5 (b). Summary of SNR prediction and robustness statistics and model classification results for 6 key elements (Ca, Fe, Mn, Sr, Ti, Zr) across eight models tested (S = Signal-dominated predictions, N = Noise-dominated predictions) see Supplementary Methods for definitions and details).

**b) Prediction SNR, Ranking & Classification**					SNR Assessment							Model Classification	
**6 El.**	Model	Robust Rank Global	Robust Rank Per Site	R^2 RMSE Rank	SNR	SNR CI	SNR smooth	SNR CI sc	SNR smooth sc	$R^2_{SS\text{-}cv}$ sc	Robust Score Global	Confidence Class (Signal or Noise)	Stability class
Ca	Bayes	1	6	2	2.306	0.416	4.174	0.891	0.953	0.869	0.903	High (S)	Stable
	PLS_LOO	2	4	3	2.224	0.402	4.103	0.854	0.841	0.840	0.846	High (S)	Stable
	PLS_kfold	3	5	4	2.224	0.402	4.103	0.854	0.841	0.840	0.846	High (S)	Stable
	RF	4	1	1	2.479	0.459	3.570	1.000	0.000	1.000	0.700	Moderate	Acceptable
	OLS_wt	5	2	8	1.867	0.449	4.199	0.975	0.992	0.000	0.687	Moderate	Acceptable
	OLS	6	3	5	1.984	0.367	4.202	0.767	0.997	0.034	0.616	Moderate	Acceptable
	WLS_wt	7	7	6	1.996	0.067	4.204	0.006	1.000	0.029	0.311	Low	Marginal
	WLS	8	8	7	1.996	0.064	4.204	0.000	1.000	0.029	0.309	Low	Marginal
Fe	OLS_wt	1	4	8	0.822	0.675	5.421	1.000	0.953	0.000	0.686	Moderate	Acceptable
	Bayes	2	3	2	1.535	0.424	2.935	0.572	0.351	0.778	0.568	Low	Acceptable
	PLS_LOO	3	1	3	1.554	0.452	2.878	0.620	0.338	0.647	0.544	Low	Acceptable
	PLS_kfold	4	2	4	1.554	0.452	2.878	0.620	0.338	0.647	0.544	Low	Acceptable
	RF	5	5	1	1.068	0.383	1.482	0.504	0.000	1.000	0.502	Low	Acceptable
	OLS	6	6	5	0.866	0.345	5.550	0.438	0.984	0.069	0.491	Low	Marginal
	WLS_wt	7	7	7	0.849	0.092	5.615	0.007	1.000	0.064	0.322	Low	Marginal
	WLS	8	8	6	0.849	0.088	5.614	0.000	1.000	0.064	0.319	Low	Marginal
Mn	PLS_LOO	1	4	3	1.444	0.355	3.374	0.906	1.000	0.419	0.788	High (S)	Stable
	PLS_kfold	2	5	4	1.444	0.355	3.374	0.906	1.000	0.419	0.788	High (S)	Stable
	RF	3	3	1	1.582	0.340	2.915	0.853	0.358	1.000	0.749	Moderate	Acceptable
	Bayes	4	1	2	1.453	0.380	2.706	0.990	0.067	0.730	0.635	Moderate	Acceptable
	OLS	5	2	5	1.316	0.383	2.685	1.000	0.037	0.281	0.496	Low	Marginal
	OLS_wt	6	6	8	1.144	0.163	2.721	0.245	0.088	0.000	0.125	Low (N)	Unstable
	WLS_wt	7	7	6	1.328	0.091	2.667	0.000	0.012	0.277	0.087	Low (N)	Unstable
	WLS	8	8	7	1.332	0.092	2.658	0.003	0.000	0.273	0.083	Low (N)	Unstable
Sr	RF	1	2	1	3.300	0.417	3.195	1.000	1.000	1.000	1.000	High (S)	Stable
	Bayes	2	5	2	3.029	0.362	2.533	0.869	0.000	0.924	0.625	Moderate	Acceptable
	PLS_LOO	3	3	3	2.473	0.307	2.648	0.736	0.174	0.658	0.544	Low	Acceptable
	PLS_kfold	4	4	4	2.473	0.307	2.648	0.736	0.174	0.658	0.544	Low	Acceptable
	OLS	5	1	5	2.396	0.256	3.044	0.614	0.771	0.124	0.514	Low	Acceptable
	WLS	6	7	6	2.415	0.031	3.054	0.073	0.788	0.124	0.303	Low	Marginal
	WLS_wt	7	6	7	2.312	0.035	2.997	0.083	0.701	0.119	0.279	Low (N)	Unstable
	OLS_wt	8	8	8	1.919	0.000	2.965	0.000	0.652	0.000	0.196	Low (N)	Unstable
Ti	Bayes	1	1	1	1.198	0.659	5.251	0.871	0.522	1.000	0.805	High (S)	Stable
	PLS_LOO	2	4	3	1.051	0.642	5.770	0.840	0.710	0.702	0.760	High (S)	Stable
	RF	3	2	2	1.397	0.620	5.367	0.801	0.564	0.758	0.717	Moderate	Acceptable
	OLS	4	6	4	1.042	0.644	6.507	0.844	0.977	0.225	0.698	Moderate	Acceptable
	OLS_wt	5	5	8	0.925	0.635	6.570	0.829	1.000	0.000	0.632	Moderate	Acceptable
	PLS_kfold	6	3	7	0.628	0.731	3.812	1.000	0.000	0.080	0.424	Low	Marginal
	WLS_wt	7	7	5	1.086	0.181	6.452	0.017	0.957	0.211	0.357	Low	Marginal
	WLS	8	8	6	1.088	0.171	6.442	0.000	0.954	0.205	0.348	Low	Marginal
Zr	OLS	1	5	5	0.350	0.832	2.534	0.812	1.000	0.579	0.799	High (S)	Stable
	WLS_wt	2	6	7	0.333	0.616	2.520	0.602	0.983	0.579	0.709	Moderate	Acceptable
	Bayes	3	4	2	0.587	0.792	1.945	0.773	0.296	0.901	0.668	Moderate	Acceptable
	RF	4	3	1	0.950	0.452	2.222	0.442	0.626	1.000	0.665	Moderate	Acceptable
	PLS_LOO	5	1	3	0.530	1.024	1.697	1.000	0.000	0.827	0.648	Moderate	Acceptable
	PLS_kfold	6	2	4	0.530	1.024	1.697	1.000	0.000	0.827	0.648	Moderate	Acceptable
	WLS	7	7	6	0.337	0.402	2.524	0.393	0.987	0.579	0.627	Moderate	Acceptable
	OLS_wt	8	8	8	0.274	0.000	2.424	0.000	0.869	0.000	0.261	Low (N)	Unstable

Supplementary Table S5(c). Summary of key performance indicators for the 4 element Ti and Zr optimised calibration runs (Ca, Sr, Ti, Zr) across eight models tested. Multivariate models are shaded grey, with PLS models are shaded dark grey. $RMSEP_{CV}$ is cross validated RMSEP, $RMSEP_b$ is bootstrapped RMSEP (see Supplementary Methods for definitions and details).

**c) Performance**		log-space			Components		Concentration-space (mg kg^{-1})						
**4 El.**	Model	$R^2_{SS\text{-}cv}$	RMSE	RMSEP	AIC	BIC	RMSE	$RMSEP_{cv}$	$RMSEP_b$	$RMSEP_b$ 1σ	Mean obs.	Mean Pred.	Mean 95% CI width
Ca	Bayes	0.637	0.4288	0.4378	318.69	340.23	5546	5677	5957	821	13400	11115	20961
	RF	0.678	0.4039	0.4053	-	-	4923	5049	5228	455	13400	10904	19127
	PLS_LOO	0.605	0.4471	0.4504	-	-	5922	6054	6206	773	13400	10800	21448
	PLS_kfold	0.605	0.4471	0.4504	-	-	5922	6054	6206	773	13400	10800	21448
	OLS_wt	0.601	0.4496	-	359.87	370.64	5621	-	5697	563	13400	10492	18036
	OLS	0.605	0.4470	0.4509	335.01	345.78	5505	5658	5697	563	13400	10921	22039
	WLS_wt	0.605	0.4474	-	321.19	331.96	5447	-	5697	563	13400	10873	114937
	WLS	0.605	0.4474	-	321.22	331.99	5446	-	5697	563	13400	10872	118586
Sr	Bayes	0.635	0.3737	0.3851	244.97	266.52	57	59	59	7	164	147	235
	RF	0.688	0.3454	0.3468	-	-	44	45	48	4	164	141	206
	PLS_LOO	0.620	0.3817	0.3874	-	-	59	60	61	7	164	145	238
	PLS_kfold	0.620	0.3817	0.3874	-	-	59	60	61	7	164	145	238
	OLS	0.606	0.3884	0.3916	259.70	270.47	60	60	61	5	164	143	243
	WLS	0.606	0.3885	-	259.48	270.25	59	-	61	5	164	143	2054
	WLS_wt	0.605	0.3888	-	262.01	272.78	62	-	61	5	164	142	1720
	OLS_wt	0.588	0.3970	-	330.65	341.42	69	-	61	5	164	132	1069088
Ti	PLS_LOO	0.756	0.6077	0.6203	-	-	1920	2043	2203	705	3392	2139	6388
	PLS_kfold	0.756	0.6077	0.6203	-	-	1920	2043	2203	705	3392	2139	6388
	OLS	0.730	0.6396	0.6444	527.01	537.78	2036	2124	2183	419	3392	2121	6994
	Bayes	0.758	0.6056	0.6179	503.77	525.31	2133	2262	2412	784	3392	2140	6362
	OLS_wt	0.719	0.6529	-	558.91	569.68	2174	-	2183	419	3392	2011	7272
	RF	0.738	0.6301	0.6247	-	-	1633	1644	1725	214	3392	2147	6757
	WLS_wt	0.729	0.6405	-	488.00	498.77	1893	-	2183	419	3392	2056	22898
	WLS	0.729	0.6408	-	487.49	498.26	1883	-	2183	419	3392	2048	23897
Zr	OLS	0.608	0.8223	0.8294	661.70	672.47	148	171	171	94	65	52	261
	WLS_wt	0.608	0.8226	-	661.82	672.59	163	-	171	94	65	54	385
	PLS_LOO	0.720	0.6949	0.7115	-	-	78	83	89	35	65	44	161
	PLS_kfold	0.720	0.6949	0.7115	-	-	78	83	89	35	65	44	161
	RF	0.743	0.6659	0.6754	-	-	46	48	50	11	65	42	142
	WLS	0.608	0.8225	-	661.73	672.50	159	-	171	94	65	54	577
	Bayes	0.722	0.6930	0.7072	575.99	597.54	84	90	94	38	65	45	165
	OLS_wt	0.401	1.0169	-	788.73	799.50	154	-	171	94	65	42	34663157

Supplementary Table S5(d). Summary of SNR prediction and robustness statistics and model classification results for the 4 element Ti and Zr optimised calibration runs (Ca, Sr, Ti, Zr) across eight models tested (S = Signal-dominated predictions, N = Noise-dominated predictions) see Supplementary Methods for definitions and details; * = best / final models chosen to optimise Ti and Zr predictions).

**d) Prediction SNR, Ranking & Classification**					SNR Assessment							Model Classification	
**4 El.**	Model	Robust Rank Global	Robust Rank Per Site	R^2 RMSE Rank	SNR	SNR CI	SNR smooth	SNR CI sc	SNR smooth sc	$R^2_{SS\text{-}cv}$ sc	Robust Score Global	Confidence Class (Signal or Noise)	Stability class
Ca	Bayes	1	4	2	2.004	0.349	4.323	0.741	0.694	0.469	0.645	Moderate	Acceptable
	RF	2	3	1	2.215	0.391	2.724	0.849	0.000	1.000	0.640	Moderate	Acceptable
	PLS_LOO	3	1	4	1.824	0.375	5.028	0.806	1.000	0.057	0.639	Moderate	Acceptable
	PLS_kfold	4	2	5	1.824	0.375	5.028	0.806	1.000	0.057	0.639	Moderate	Acceptable
	OLS_wt	5	5	8	1.867	0.449	4.199	1.000	0.640	0.000	0.592	Low	Acceptable
	OLS	6	6	3	1.984	0.367	4.202	0.787	0.641	0.059	0.525	Low	Acceptable
	WLS_wt	7	7	6	1.996	0.067	4.204	0.006	0.642	0.051	0.210	Low (N)	Unstable
	WLS	8	8	7	1.996	0.064	4.204	0.000	0.642	0.051	0.208	Low (N)	Unstable
Sr	Bayes	1	1	2	2.600	0.294	3.313	0.777	0.858	0.469	0.709	Moderate	Acceptable
	RF	2	5	1	3.182	0.378	2.669	1.000	0.000	1.000	0.700	Moderate	Acceptable
	PLS_LOO	3	3	3	2.463	0.284	3.420	0.749	1.000	0.311	0.693	Moderate	Acceptable
	PLS_kfold	4	4	4	2.463	0.284	3.420	0.749	1.000	0.311	0.693	Moderate	Acceptable
	OLS	5	2	5	2.396	0.256	3.044	0.677	0.499	0.175	0.473	Low	Marginal
	WLS	6	7	6	2.415	0.031	3.054	0.081	0.514	0.175	0.239	Low (N)	Unstable
	WLS_wt	7	6	7	2.312	0.035	2.997	0.091	0.437	0.168	0.218	Low (N)	Unstable
	OLS_wt	8	8	8	1.919	0.000	2.965	0.000	0.394	0.000	0.118	Low (N)	Unstable
Ti	PLS_LOO*	1	2	2	1.114	0.678	5.761	0.986	0.529	0.959	0.841	High (S)	Stable
	PLS_kfold*	2	3	3	1.114	0.678	5.761	0.986	0.529	0.959	0.841	High (S)	Stable
	OLS	3	6	5	1.042	0.644	6.507	0.919	0.964	0.289	0.744	Moderate	Acceptable
	Bayes	4	1	1	1.004	0.685	4.899	1.000	0.026	1.000	0.708	Moderate	Acceptable
	OLS_wt	5	5	8	0.925	0.635	6.570	0.903	1.000	0.000	0.661	Moderate	Acceptable
	RF	6	4	4	1.315	0.584	4.854	0.804	0.000	0.492	0.469	Low	Marginal
	WLS_wt	7	7	6	1.086	0.181	6.452	0.019	0.931	0.271	0.368	Low	Marginal
	WLS	8	8	7	1.088	0.171	6.442	0.000	0.925	0.264	0.357	Low	Marginal
Zr	OLS	1	2	5	0.350	0.832	2.534	1.000	1.000	0.606	0.882	High (S)	Stable
	WLS_wt	2	6	7	0.333	0.616	2.520	0.741	0.976	0.605	0.771	High (S)	Stable
	PLS_LOO*	3	4	3	0.567	0.713	2.116	0.857	0.283	0.933	0.708	Moderate	Acceptable
	PLS_kfold*	4	5	4	0.567	0.713	2.116	0.857	0.283	0.933	0.708	Moderate	Acceptable
	RF	5	1	1	0.914	0.431	2.274	0.518	0.553	1.000	0.673	Moderate	Acceptable
	WLS	6	7	6	0.337	0.402	2.524	0.484	0.982	0.605	0.670	Moderate	Acceptable
	Bayes	7	3	2	0.538	0.778	1.951	0.935	0.000	0.938	0.655	Moderate	Acceptable
	OLS_wt	8	8	8	0.274	0.000	2.424	0.000	0.811	0.000	0.243	Low (N)	Unstable

Supplementary Table S6 Calibration model validation and diagnostic tests for 4-elements (Ca, Sr, Ti, Zr) across calibration model runs (Ti and Zr optimised components, based on jackknifing results). Shapiro–Wilks Test is used to assess whether the dataset is normally distributed with values ~1 and $p > 0.05$ likely to be normal. Values <1 and $p < 0.05$ deviate from normality. AIC (Akaike Information Criterion) and BIC (Bayesian Information Criterion) were used to compare model goodness of fit with model complexity in preliminary univariate and Bayesian model runs only. Lower AIC and BIC values are better and AIC preferable if a prediction-focussed model; BIC is more favourable for Bayesian models. Breusch-Pagan tests and AIC and BIC were indicative only and not used for ranking model performance as they are not calculable for all models. He = Heteroscedasticity, Ho = Homoscedasticity, i.e., the residuals are distributed with equal variance.

El	Model	MV	Sharp Wilks	p-value	Normal Dist.	Breusch-Pagan	p-value	hetero- / homo-scedasticity	k-fold CV10 RMSE	AIC	BIC
Ti	OLS		0.9862	0.011127	-	13.96	0.00019	He	0.6444	527.01	537.78
	WLS		0.9873	0.018361	-	1552	0.00000	He	-	487.49	498.26
	OLS_wt		0.9842	0.004619	-	6.91	0.00859	He	-	558.91	569.68
	WLS_wt		0.9872	0.017264	-	1333	0.00000	He	-	488.00	498.77
	Bayes	M	0.9808	0.001143	-	12.97	0.01144	Ho	0.6179	503.77	525.31
	RF	M	0.9737	0.000076	-	-	-	-	0.6247	-	-
	PLS_LOO	M	0.9811	0.001271	-	-	-	-	0.6203	-	-
	PLS_kfold	M	0.9811	0.001271	-	-	-	-	0.6203	-	-
Ca	OLS		0.9820	0.001873	-	7.61	0.00580	He	0.4509	335.01	345.78
	WLS		0.9802	0.000875	-	2323	0.00000	He	-	321.22	331.99
	OLS_wt		0.9833	0.003155	-	13.29	0.00027	He	-	359.87	370.64
	WLS_wt		0.9803	0.000900	-	2244	0.00000	He	-	321.19	331.96
	Bayes	M	0.9832	0.003094	-	6.10	0.19173	Ho	0.4378	318.69	340.23
	RF	M	0.9795	0.000676	-	-	-	-	0.4053	-	-
	PLS_LOO	M	0.9769	0.000245	-	-	-	-	0.4504	-	-
	PLS_kfold	M	0.9769	0.000245	-	-	-	-	0.4504	-	-
Sr	OLS		0.9306	0.000000	-	0.10	0.75393	Ho	0.3916	259.70	270.47
	WLS		0.9307	0.000000	-	3121	0.00000	He	-	259.48	270.25
	OLS_wt		0.9316	0.000000	-	29186	0.00000	He	-	330.65	341.42
	WLS_wt		0.9307	0.000000	-	2696	0.00000	He	-	262.01	272.78
	Bayes	M	0.9360	0.000000	-	12.27	0.01548	Ho	0.3851	244.97	266.52
	RF	M	0.9298	0.000000	-	-	-	-	0.3468	-	-
	PLS_LOO	M	0.9333	0.000000	-	-	-	-	0.3874	-	-
	PLS_kfold	M	0.9333	0.000000	-	-	-	-	0.3874	-	-
Zr	OLS		0.9897	0.054411	N	0.04	0.85119	Ho	0.8294	661.70	672.47
	WLS		0.9896	0.053293	N	253.04	0.00000	He	-	661.73	672.50
	OLS_wt		0.9882	0.027180	-	17737	0.00000	He	-	788.73	799.50
	WLS_wt		0.9896	0.052602	N	44.19	0.00000	He	-	661.82	672.59
	Bayes	M	0.9923	0.176863	N	13.96	0.00744	He	0.7072	575.99	597.54
	RF	M	0.9933	0.274908	N	-	-	-	0.6754	-	-
	PLS_LOO	M	0.9913	0.111549	N	-	-	-	0.7115	-	-
	PLS_kfold	M	0.9913	0.111549	N	-	-	-	0.7115	-	-

Supplementary Table S7 Summary prediction model statistics derived from the cross validated (CV, k-fold with jackknifing) for the final PLS regression training model (4 elements: Ca, Ti, Sr, Zr). RMSEP and CI have been converted from calibration space into predicted concentrations in mg kg^{-1}; final XRF-CS composite depth-matched dataset ($n = 268$; $p<0.001$)

Element	R^2 SS CV-kfold-jk (100)	RMSEP CV-kfold-jk (100)	R^2 SS pred-LM	RMSEP single cv pred	-RMSEP CI lower	+RMSEP CI upper
Ti	0.758	0.618	0.874	2136	-1106	4297
Ca	0.637	0.436	0.751	5548	-6559	9663
Sr	0.635	0.380	0.764	57	-66	135
Zr	0.722	0.706	0.607	85	-23	85

Supplementary Table S8 Summary ICP-MS subsample depth, thickness and interval statistics for the whole ACE dataset and the five individual sites.

Site	n	Mean Subsample Thickness (cm)	SD Subsample Thickness (cm)	Mean Interval (cm)	Mean Interval error (cm)	SD Interval (cm)	SE Interval (cm)	Min. Interval (cm)	Max. Interval (cm)
ACE	268	1.2	0.1	5.5	0.8	3.3	0.2	0.3	16.5
BI10	68	1.0	0.1	7.5	0.7	3.7	0.4	1.1	15.9
HER42PB	66	1.2	0.1	6.0	0.9	3.3	0.4	0.3	16.5
KER1	52	1.3	0.1	4.7	0.9	2.9	0.4	1.4	10.7
KER3	51	1.1	0.1	4.0	0.8	1.9	0.3	1.2	7.9
PB1	31	1.2	0.1	3.3	0.8	2.2	0.4	1.2	8.2

Supplementary Table S9 Summary ICP-MS subsample age, accumulation rate and interval statistics for the whole ACE dataset and the five individual sites.

Site	n	Mean Interval (years)	Mean Interval error (years)	SD Interval (years)	SE Interval (years)	Mean accum. rate (cm yr^{-1})	SD accum. rate (cm yr^{-1})	Mean dry mass accum. rate (g cm^{-2} yr^{-1})	SD dry mass accum. rate (g cm^{-2} yr^{-1})
ACE	268	133	53	130	8	0.0634	0.0486	0.0216	0.0258
BI10	68	122	35	106	13	0.0826	0.0395	0.0204	0.0126
HER42PB	66	77	25	44	5	0.0947	0.0665	0.0351	0.0432
KER1	52	153	55	141	20	0.0409	0.0160	0.0222	0.0164
KER3	51	220	122	185	26	0.0326	0.0243	0.0081	0.0120
PB1	31	102	34	91	16	0.0429	0.0246	0.0168	0.0102

Supplementary Table S10. The following R packages were used in this study in R version 4.4.2 (2024-10-31) -- "Pile of Leaves" and RStudio Version 2024.12.1+563 (2024.12.1+563) (Mac versions). We acknowledge the use of ChatGPT Plus (OpenAI, Versions GPT-4o, GPT -5.1, GPT -5.2) for assistance with generating functions and additional code based on, or expanded from, code originally written by the authors, and the use of function embedded in the R packages available on CRAN listed. Implementations of the functions and code, logged in a .txt file verified, have been error-checked and debugged by corresponding author SR. Manual R code for the Ti- and Zr-optimised PLS calibration model, with validation tests and plots included, has been packaged into a series of functions in this file: ACE_PLS_log_inc_v2. Input data and all outputs can be found in this folder. Manual R code for the preliminary calibration exercise, including all validation tests, bootstrapping, prediction data and output plots has been packaged into a series of functions in this file: Calibration_function_Multivariate_v5.R – further details below and in the description at the start of the file. These calibration function R files have

dependencies on existing packages such as *itrax.R*[17] (by Tom Bishop), compositions, pls, arm, Tidyverse and other packages loaded at the start and listed in Supplementary Table S10. All input data and code relating to the calibration models can be found in this folder. All datasets imported into R and code used for analysing and plotting data in this study can be found in this Github folder: https://github.com/stever60/ACE_peat_calibration.

Package Version	Weblink	Summary
arm v. 1.14-4	https://github.com/suyusung/arm/issues/	Bayesian glm regression model
bestNormalize v1.9.1	https://github.com/petersonR/bestNormalize	Data wrangling/transformation
boot v. 1.3-32	https://cloud.r-project.org/package=boot	Bootstrapping functions
broom v1.0.7	https://github.com/tidymodels/broom	Summarizes statistical objects in tidyverse
car v. 3.1-3	Long link	Functions for Applied Regression
caret v. 7.0-1	https://github.com/topepo/caret/	Functions for training, plot classification and regression
chemometrics v. 1.4. 4	http://cstat.tuwien.ac.at/filz/	Multivariate Statistical Analysis
cluster v. 2.1.8	https://svn.r-project.org/R-packages/trunk/cluster/	Various methods for cluster analysis
colorspace v. 2.1-1	doi:10.18637/jss.v096.i01	Assessing colour blind friendliness of plots
compositions 2.0-8	http://www.stat.boogaart.de/compositions/	Tools geochemical compositional analysis
cowplot v. 1.1.3	https://github.com/wilkelab/cowplot/issues	Creating publication figures in ggplot2
dendextend v. 1.19.0	https://doi.org/10.1093/bioinformatics/btv428	Useful functions dendrograms, clustering
directlabels v. 2024.1.21	https://github.com/tdhock/directlabels	Labelling data in ggplot2 plots
dplyr v. 2.5.0	https://dplyr.tidyverse.org	Tidyverse required code
dynamicTreeCut v. 1.63-1	Long link	Detecting significant clusters in dendrograms
ellipse v. 0.5.0	Long Link	Adding ellipse-like confidences to plots
errors v. 0.4.3	Long Link	Measurement errors, uncertainty propagation
factoextra v. 1.0.7	http://www.sthda.com/english/rpkgs/factoextra	Visualization of multivariate analyses
forecast v. 8.23.0	https://pkg.robjhyndman.com/forecast/	Time series forecasts and smoothing
future v. 1.68	https://future.futureverse.org	Future API for sequential, parallel processing of R code
ggdendro v. 0.2.0	https://andrie.github.io/ggdendro/	Making dendrograms in ggplot2
ggplot2 v. 4.0.1	https://ggplot2.tidyverse.org	Core plotting in Tidyverse R suite
ggpmisc v. 0.6.1	https://docs.r4photobiology.info/ggpmisc/	Adding P-values, R2 etc to ggplot2 plots
ggpubr v. 0.6.2	https://rpkgs.datanovia.com/ggpubr/	Making publication ready plots in ggplot2
ggrepel v. 0.9.6	https://ggrepel.slowkow.com/	Avoids overlaps in labels in ggplot2
ggsci v. 3.2.0	https://nanx.me/ggsci/	Colour palettes for scientific journals
Hmisc v. 5.2-2	https://hbiostat.org/R/Hmisc/	Various functions for data analysis
itraxR v.1.12.2	https://github.com/tombishop1/itraxR/	Itrax sediment core data parsing & analysis
lmtest v. 0.9-40	https://cran.r-project.org/web/packages/lmtest/index.html	Tests and diagnostics for regression models
magrittr v. 2.0.3	https://magrittr.tidyverse.org	Chaining commands in Tidyverse
performance v. 0.15.3	https://doi.org/10.1098/rsif.2017.0213	Assessing model quality beyond base R stats
PeriodicTable v. 0.1.2	https://cran.r-project.org/web/packages/PeriodicTable/index.html	List of chemical elements and properties
pls v. 2.8.5	https://github.com/khliland/pls	Multivariate regression methods inc. PLSR
psych v. 2.5.6	https://personality-project.org/r/psych/	Functions for multivariate data analysis
randomForest 4.7-1.2	https://doi.org/10.1023/A:1010933404324	Breiman/Cutlers Random Forests for Classification & Regression
rbacon v. 3.3.1	https://cran.r-project.org/web/packages/rbacon/index.html	Bayesian age-depth modelling in R
RColorBrewer v. 1.1-3	https://cran.r-project.org/web/packages/RColorBrewer/index.html	Various CBF palettes for plotting
readr v. 2.1.5	https://readr.tidyverse.org	Tidyverse core package for importing data
rioja v. 1.0-7	https://github.com/nsj3/rioja	Quaternary science data analysis
sjmisc v. 2.8.10	https://strengejacke.github.io/sjmisc/	Data transformation functions
tidypaleo v. 0.1.3	https://paleolimbot.github.io/tidypaleo/	Palaeo data management and plotting
tidyverse v. 2.0.0	https://tidyverse.tidyverse.org	Set of packages - alternative to Base R coding
vegan v. 2.6-10	https://github.com/vegandevs/vegan	Ecological ordination and diversity analysis
viridis v. 0.6.5	https://sjmgarnier.github.io/viridis/	Colour blind friendly mapping & scaling
wesanderson v. 0.3.7	https://github.com/karthik/wesanderson	Colour blind friendly colour schemes

For the calibration exercise, code and functions for individual regression were written to undertake the univariate and multivariate calibration workflow (Supplementary Figure 1) for multiple geochemical elements, with log transformed ICP-MS calibration data and log transformed XRF datasets as inputs. The functions were updated to include Random Forest (RF) using Out-of-Bag (OOB) regression – a method for estimating a random forest model's performance without using a separate test set - using the *randomForest.R* package[2]. This works by each tree in the forest evaluating the data points that were not included in its own training sample (i.e., the "out-of-bag", or OOB, data). By averaging these predictions across all trees and their respective OOB samples, the OOB error provides an unbiased estimate of the RF model's error for each element.

For each element, the *Calibration_function_Multivariate_v5.R* file automatically fits and tests eight calibration models (OLS, WLS, weighted OLS, weighted WLS, a Bayesian Generalised Linear Model (Bayes glm), Random Forest (RF), PLS with LOO, and PLS with k-fold). It evaluates model performance using R^2, RMSE, 10-fold and bootstrapped RMSEP, AIC/BIC (where applicable), and runs diagnostic tests for normality, outliers, and

heteroscedasticity. For each of the input elements (which are defined separately by the user), it produces a series of predicted vs. observed, residual assessment and other diagnostic test plots, as well as residual and influence summary diagnostics as txt or csv files. It produces initial model performance metric rankings based on R^2 (highest is best), then RMSE (lowest is best) and then RMSEP (lowest is best). The next stage of the function generates concentration space predictions with 95% confidence intervals for a new XRF dataset, defined and inputted as a *.csv* file by the user. This produces multi-element comparison plots for depth and age for each site for all 8 models. Finally, it compiles summary tables of key performance and prediction metrics in log and concentration space, saves prediction data for each element as a *.csv* file, and organises all outputs into folders.

Supplementary References

1. Mevik, B.-H. & Wehrens, R. The pls Package: Principal Component and Partial Least Squares Regression in R. *J. Stat. Soft.* **18**, 1–23 (2007).
2. Breiman, L. Random Forests. *Machine Learning* **45**, 5–32 (2001).
3. Gelman, A. & Hill, J. *Data Analysis Using Regression and Multilevel/Hierarchical Models*. (Cambridge University Press, Cambridge, 2006). doi:10.1017/CBO9780511790942.
4. Mann, M. E. & Lees, J. M. Robust estimation of background noise and signal detection in climatic time series. *Climatic Change* **33**, 409–445 (1996).
5. Telford, R. J. & Birks, H. J. B. A novel method for assessing the statistical significance of quantitative reconstructions inferred from biotic assemblages. *Quaternary Science Reviews* **30**, 1272–1278 (2011).
6. Juggins, S. & Birks, H. J. B. Quantitative Environmental Reconstructions from Biological Data. in *Tracking Environmental Change Using Lake Sediments: Data Handling and Numerical Techniques* (eds. Birks, H. J. B., Lotter, A. F., Juggins, S. & Smol, J. P.) 431–494 (Springer Netherlands, Dordrecht, 2012). doi:10.1007/978-94-007-2745-8_14.
7. Sun, P., Holden, P. B. & Birks, H. J. B. Can machine-learning algorithms improve upon classical palaeoenvironmental reconstruction models? *Clim. Past* **20**, 2373–2398 (2024).
8. Pearson, E. J. *et al.* Development of new global lake brGDGT-temperature calibrations: advances, applications, challenges, and recommendations. *Quaternary Science Reviews* **369**, 109615 (2025).
9. Bertrand, S. *et al.* Inorganic geochemistry of lake sediments: A review of analytical techniques and guidelines for data interpretation. *Earth-Science Reviews* **249**, 104639 (2024).
10. Tjallingii, R., Röhl, U., Kölling, M. & Bickert, T. Influence of the water content on X-ray fluorescence core-scanning measurements in soft marine sediments. *Geochemistry, Geophysics, Geosystems* **8**, (2007).
11. van den Boogaart, K., Tolosana-Delgado, R. & Bren, M. compositions: Compositional Data Analysis. R package version 2.0-8. https://CRAN.R-project.org/package=compositions (2024).
12. Lüdecke, D., Ben-Shachar, M., Patil, I., Waggoner, P. & Makowski, D. Performance: An R package for assessment, comparison and testing of statistical models. *J. Open Source Softw.* **6**, 3139 (2021).
13. Boyle, J. F., Chiverrell, R. C. & Schillereff, D. Approaches to Water Content Correction and Calibration for µXRF Core Scanning: Comparing X-ray Scattering with Simple Regression of Elemental Concentrations. in *Micro-XRF Studies of Sediment Cores* (eds. Croudace, I. W. & Rothwell, R. G.) 373–390 (Springer Netherlands, Dordrecht, 2015).
14. Klingenfuß, C., Roßkopf, N., Walter, J., Heller, C. & Zeitz, J. Soil organic matter to soil organic carbon ratios of peatland soil substrates. *Geoderma* **235–236**, 410–417 (2014).
15. Chawchai, S., Kylander, M. E., Chabangborn, A., Löwemark, L. & Wohlfarth, B. Testing commonly used X-ray fluorescence core scanning-based proxies for organic-rich lake sediments and peat. *Boreas* **45**, 180–189 (2016).
16. Blaauw, M. & Christen, J. A. Flexible paleoclimate age-depth models using an autoregressive gamma process. *Bayesian Analysis* **6**, 457–474 (2011).
17. Bishop, T. itraxR: Itrax Data Analysis Tools. https://CRAN.R-project.org/package=itraxR (2021).
